# Chemical synthesis of the EPF-family of plant cysteine-rich proteins and late-stage dye attachment by chemoselective amide-forming ligations†

**DOI:** 10.1039/d2cb00155a

**Published:** 2022-10-19

**Authors:** Nandarapu Kumarswamyreddy, Ayami Nakagawa, Hitoshi Endo, Akie Shimotohno, Keiko U. Torii, Jeffrey W. Bode, Shunsuke Oishi

**Affiliations:** Institute of Transformative Bio-Molecules (WPI-ITbM), Nagoya University Chikusa Nagoya 464-8602 Japan oishi@itbm.nagoya-u.ac.jp; Department of Chemistry, Indian Institute of Technology Tirupati Tirupati 517619 Andhra Pradesh India; Howard Hughes Medical Institute and Department of Molecular Biosciences, The University of Texas at Austin Austin TX 78712 USA; Department of Chemistry and Applied Biosciences, ETH Zürich Zürich 8093 Switzerland

## Abstract

Chemical protein synthesis can provide well-defined modified proteins. Herein, we report the chemical synthesis of plant-derived cysteine-rich secretory proteins and late-stage derivatization of the synthetic proteins. The syntheses were achieved with distinct chemoselective amide bond forming reactions – EPF2 by native chemical ligation (NCL), epidermal patterning factor (EPF) 1 by the α-ketoacid-hydroxylamine (KAHA) ligation, and fluorescent functionalization of their folded variants by potassium acyltrifluoroborate (KAT) ligation. The chemically synthesized EPFs exhibit bioactivity on stomatal development in *Arabidopsis thaliana*. Comprehensive synthesis of EPF derivatives allowed us to identify suitable fluorescent variants for bioimaging of the subcellar localization of EPFs.

## Abstract 2

Comprehensive synthesis and late-stage functionalization strategy of EPF derivatives allowed us to identify suitable fluorescent variants for bioimaging of the subcellar localization of EPFs.

## Graphical abstract

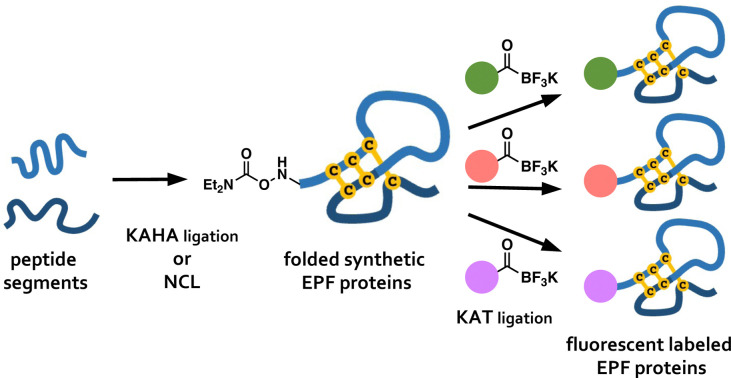

## Introduction

Chemically functionalized proteins are important research tools for elucidating and visualizing biological pathways. Reporter groups, such as fluorescent dyes^1,2^ and radioactive groups,^3,4^ introduced onto protein probes enable the monitoring of their dynamics in complex biological systems. A number of labeling methods were developed to introduce functionalities onto natural residues of proteins,^5–8^ including attachments to lysine,^9–11^ cysteine,^12–15^ tyrosine,^16^ tryptophan,^17^ methionine,^18^ N- or C-termini.^19^ However, these techniques usually result in a heterogeneous mixture of functionalized proteins as most proteins contain multiple reactive natural residues.

Chemical synthesis of proteins, accomplished by chemoselective amide bond forming reactions *i.e.* peptide ligations, provides an alternative approach to structurally uniform protein probes.^20–26^ Precisely controlled structure of chemically synthesized protein probes could exclude artefacts caused by a heterogeneous or structurally-deficient protein probes.^27–29^ Despite the advantage of chemically synthesized protein probes, synthesis is often a tedious and time-consuming process, especially if key reporter groups are introduced at the early stage of synthesis by incorporating modified amino acid monomers at the peptide elongation process. After construction of the peptide chain, peptides are cleaved from the resin, purified by HPLC, ligated to yield proteins, and finally refolded into the biologically active form. Optimization of the reporter groups often requires that nearly the entire synthesis needs to be repeated to give variants of the probe. As the result, optimization of the probe structure for each biological experiment often becomes a bottleneck.

In order to easily decorate folded proteins, we have developed the KAT ligation, a rapid chemoselective amide bond-forming reaction between potassium acyltrifluoroborates (KATs) and *O*-carbamoylhydroxylamines.^4,30–34^ KAT ligation has been applied in site-specific functionalization, such as PEGylation, lipidation, biotinylation, and dye labeling of chemically synthesized peptides and proteins bearing hydroxylamine at low concentrations in aqueous buffers with near equimolar amounts of the ligation partners.^30,32,35–37^ Functionalization of chemically synthesized proteins bearing hydroxylamine using KATs would allow us to access protein probes bearing various reporter groups and select quickly suitable protein probes for chemical biology studies.

We chose the EPF-family of secretory cysteine-rich proteins (CRPs) for our study.^38–40^ CRPs are a ubiquitous family of proteins found in all kingdoms of life and often play crucial roles in intercellular signaling.^41–47^ Particularly in plants, CRPs participate in reproductive processes,^48–50^ tissue and seed development,^51–54^ and plant immune systems.^55–57^ Among the eleven EPF members, at least three of them, EPFL9 (also known as STOMAGEN), EPF2, and EPF1 play a key role in regulating formation of stomata, which control gas exchange by opening and closing of pores on the plant surface.^39,40,58–62^

**Fig. 1:**
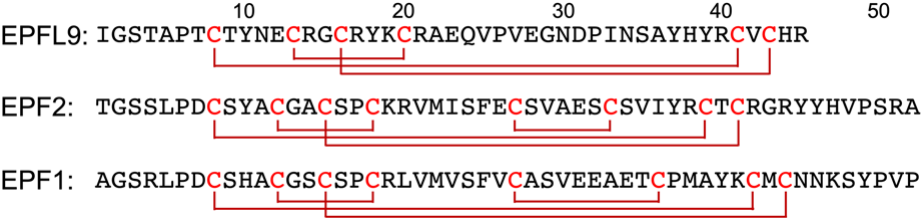
Amino acid sequences of EPFL9, EPF2, and EPF1.

EPFL9, EPF2, and EPF1 contain 45–52 amino acid residues with six or eight cysteine residues within the protein sequence (Fig. 1).^63^ These cysteine residues require disulfide bond formation to impart biological functions into the proteins, and on the other hand cysteine residues cause low-yielding of recombinant expression of EPFs in *E. coli* due to premature disulfide formations and protein aggregation.^64,65^ High purity of synthetic precursors of oxidative folding is advantageous to obtain folded active EPFs. Furthermore, late-stage chemical functionalization of folded EPFs will enable us to develop structurally well-defined protein probes and choose a suitable functionalization for chemical biology studies of EPF proteins.

In this report, we document the chemical synthesis of EPF-family plant CRPs and late-stage functionalization by chemoselective amide bond forming reactions. EPFL9 was synthesized by solid-phase peptide synthesis (SPPS), EPF2 through native chemical ligation (NCL), and EPF1 *via* chemoselective α-ketoacid-hydroxylamine (KAHA) ligation. After folding, the synthetic CRPs were functionalized with various fluorescent dyes by chemoselective KAT ligation onto a hydroxylamine moiety installed at the N-terminus. This late-stage functionalization strategy allowed us to produce and evaluate a varieties of protein probes. The chemically synthesized EPFL9, EPF2, EPF1, and their variants exhibited biological activity in *Arabidopsis* plants.

## Results and discussion

### Synthesis of EPFs by Fmoc SPPS

**Fig. 2:**
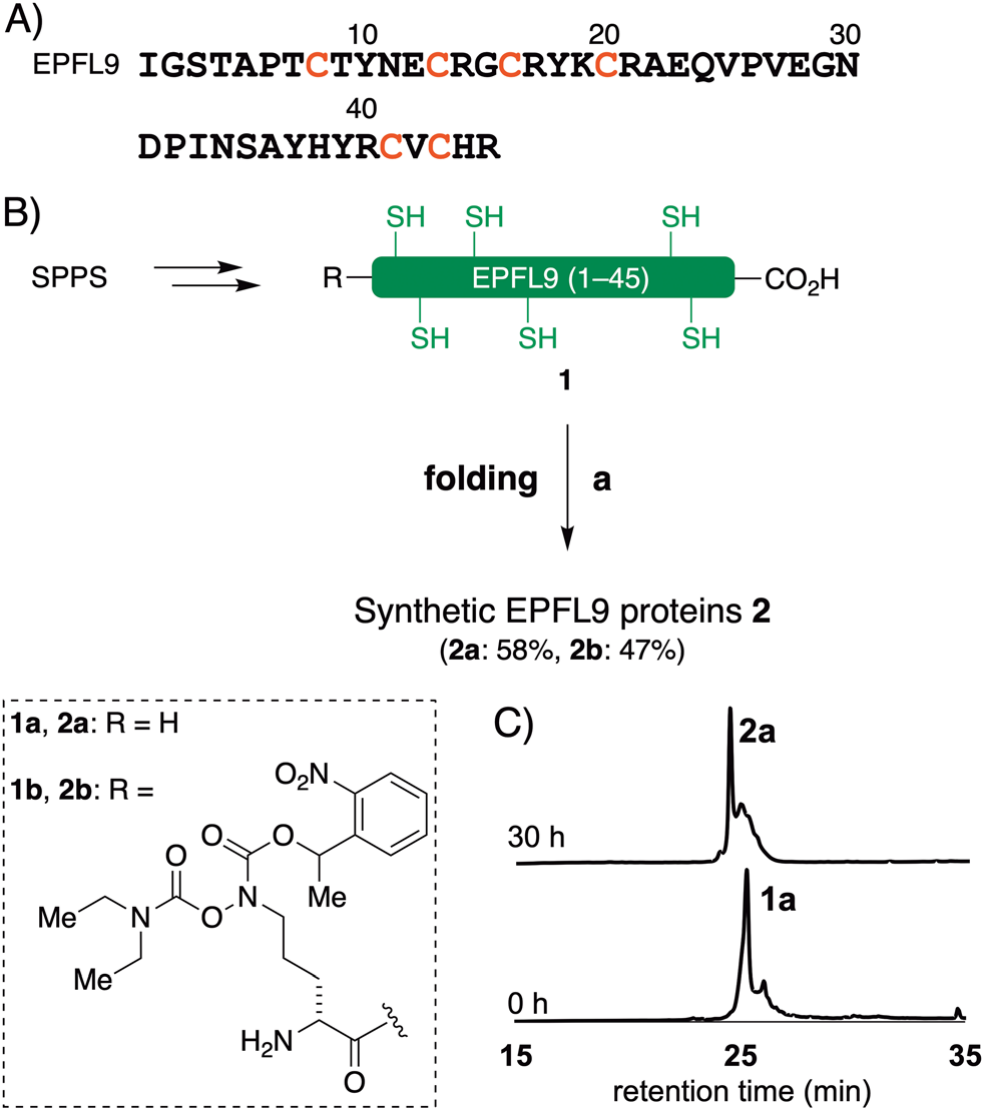
Chemical synthesis of EPFL9. (A) Amino acid sequence of EPFL9. (B) Chemical synthesis of EPFL9. Reagents and conditions: (a) 6 M Gdn·HCl, 0.1 M tris pH 6.8 then add 0.1 M tris 5.0 mM reduced glutathione 2.5 mM oxidized glutathione pH 8.0, 4 °C, 30 h. (C) Analytical HPLC traces (*λ* = 220 nm) of monitoring of folding of 1a.

The reduced EPFL9 1a was successfully synthesized by standard Fmoc SPPS and isolated in 42% yield after RP-HPLC purification (Fig. 2). However, the coupling efficiency of Cys27, Ser28, and Val29 in EPF2, and Phe25, Val26, and Cys27 in EPF1 were very low and the target peptides were not observed after these residues. Therefore, we elected to employ peptide ligation strategy by assembling peptide segments of EPF2 and EPF1 proteins into full length proteins.

### Synthesis of EPF2 protein by NCL

**Fig. 3:**
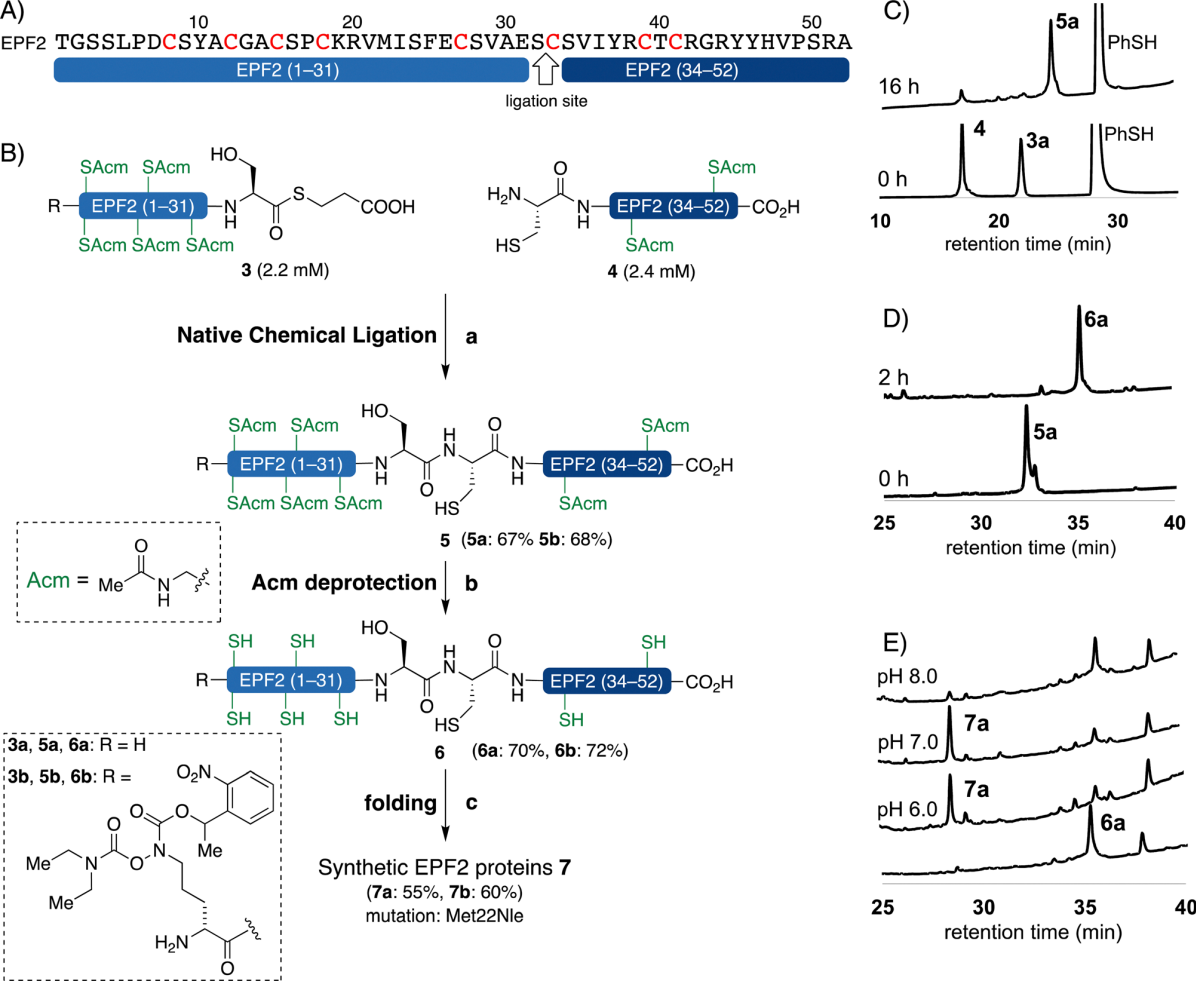
Chemical synthesis of EPF2 by NCL. (A) Amino acid sequence of EPF2 showing the ligation site. (B) Chemical synthesis of EPF2 by NCL, reagents and conditions: (a) 3% (v/v) PhSH, 6 M Gdn·HCl, 0.2 M Na_2_HPO_4_, 100 mM TCEP, 100 mM sodium ascorbate, pH = 7.4, rt, 16 h, 5a: 67% 5b: 68%; (b) 1% AgOAc in 1 : 1 AcOH/H_2_O (w/v/v), 45 °C, 2 h; (c) 6 M Gdn·HCl, 0.1 M tris buffer pH 6.8, then add 0.1 M tris, 5.0 mM reduced glutathione 2.5 mM oxidized glutathione, pH 7.0, 4 °C, 30 h. (C) Analytical HPLC traces (*λ* = 220 nm) of monitoring the ligation between 3a and 4. (D) Analytical HPLC traces (*λ* = 220 nm) of monitoring the Acm deprotection of 5a. (E) Analytical HPLC traces (*λ* = 220 nm) of folding of 6a.

We carefully examined the amino acid sequence of EPF2 and designed a ligation site between Ser32–Cys33 for NCL (Fig. 3).^21–25^ The peptide thioester segment 3a can be prepared from a peptide hydrazide precursor, which can be synthesized by standard Fmoc SPPS.^66^ Methionine residues Met22 was substituted with norleucine (Nle) and all cysteine residues were protected with Acm protecting groups.

The peptide thioester segment 3a was obtained *via* Fmoc SPPS on 0.47 mmol scale using 2-Cl-(Trt)-NHNH_2_ resin followed by oxidation and addition of β-mercaptopropionic acid^66^ (see ESI,† Section S3.1). The peptide segment 4 containing a cysteine residue at the N-terminus was synthesized on a 0.32 mmol scale by standard Fmoc SPPS. After trifluoroacetic acid (TFA) cleavage from the resin, the resulting peptide was purified and obtained 260 mg of segment 4 in 32% yield.

With segments 3a and 4 in hand, we examined NCL between peptide thioester segment 3a (1.0 equiv.) and peptide segment 4 (1.1 equiv.) with optimized reaction conditions (6 M Gdn·HCl, 0.2 M Na_2_HPO_4_, 100 mM TCEP, 100 mM sodium ascorbate, 3% (v/v) thiophenol, pH 7.4) gave the ligated peptide 5a in 67% yield (Fig. 3B). The cysteine Acm group of 5a was deproptected and 6a was purified by preparative RP-HPLC and isolated in 70% yield.

### Synthesis of EPF1 protein by KAHA ligation

The KAHA ligation^67–75^ operates under acidic conditions with water/organic solvent mixture ideal for solubilizing hydrophobic segments and resulting in more soluble peptide esters such as *O*-acyl isopeptide or depsi peptide as a primary ligation product. After rearrangement of the ligated product under the basic conditions, the resulting polypeptide contains a non-canonical amino acid residue homoserine (Hse), when using 5-oxaproline as a ligation handle on the N-terminus of the C-terminal peptide fragment.^67,71^

**Fig. 4:**
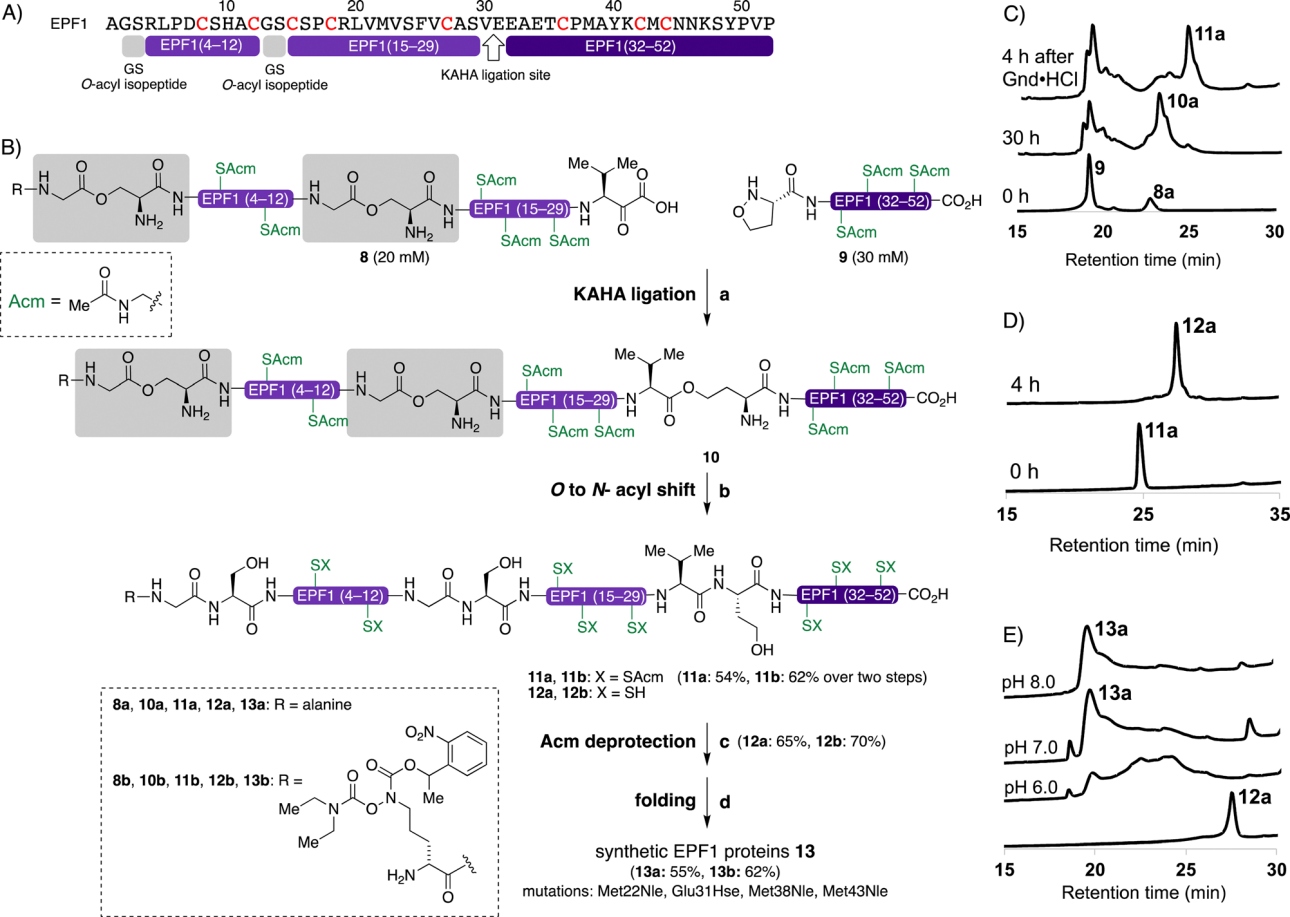
Chemical synthesis of EPF1 by KAHA ligation. (A) Amino acid sequence of EPF1 showing the ligation site and Gly–Ser *O*-acylisopeptides. (B) Chemical synthesis of EPF1 by KAHA ligation, reagents and conditions: (a) 0.1 M oxalic acid 9 : 1 DMSO/H_2_O (v/v), 60 °C, 30 h; (b) 6 M Gdn·HCl, pH 9.6, 4 h, rt; (c) 1% AgOAc in 1 : 1 AcOH/H_2_O (w/v/v), 45 °C, 2 h: (d) 6 M Gdn·HCl, 0.1 M tris buffer pH 6.8 then add 0.1 M tris, 5.0 mM reduced glutathione, 2.5 mM oxidized glutathione, pH 8.0, 4 °C, 30 h. (C) Analytical HPLC traces (*λ* = 220 nm) of monitoring the ligation between 8a and 9, and *O* to *N*-acyl shift. (D) Analytical HPLC traces (*λ* = 220 nm) of monitoring the Acm deprotection of 11a. (E) Analytical HPLC traces (*λ* = 220 nm) of folding of 12a.

We designed the KAHA ligation site between Val30–Glu31 as SPPS was successful up to Ala28 from the C-terminus (Fig. 4). The preparation of peptides bearing C-terminal valine α-ketoacids is well established. The ligation site at this particular position introduces a Hse residue as a Glu31Hse mutation. The three methionine residues Met28, Met38, Met43 were substituted with Nle to avoid oxidation during handling, storage and folding (Fig. 4B). We expected that these changes in the protein sequence would be unlikely to have a strong effect on protein structure and biological activity. We selected acetamidomethyl (Acm) protecting group for all cysteine residues to avoid premature formation of intra- and intermolecular disulfide bond formation during HPLC purification and ligation of the peptide segments.

We performed the synthesis of peptide segment EPF1 (1–30) with an α-ketoacid using standard Fmoc-protected amino acids and protected valine α-ketoacid resin with 0.25 mmol g^−1^. After TFA cleavage from the resin, the resulting peptide was difficult to analyze and purify with RP-HPLC due to poor solubility to aqueous acetonitrile with 0.1% TFA. To overcome the solubility issue, we incorporated two Gly–Ser *O*-isoacyl dipeptide units into the peptide segment. This strategy was developed by Mutter and Kiso groups, and known to enhance the solubility of the resulting synthetic peptide.^76–79^ This Gly–Ser *O*-isoacyl dipeptide strategy fits well with the KAHA ligation, as the esters can be rearranged into peptide bonds under basic conditions together with depsipeptide bond resulted by the KAHA ligation.^80^ We resynthesized segment 8a with two *N*_α_-boc protected Gly–Ser *O*-isoacyl dipeptides at position 2–3 and 13–14 during Fmoc-SPPS with 0.25 mmol g^−1^ on protected valine α-ketoacid resin. We were pleased to observe good solubility of peptide segment 8a containing two *O*-isoacyl dipeptides in aqueous acetonitrile, and the crude peptide segment was purified by RP-HPLC; and 102 mg of pure segment 8a were isolated in 12% yield (see ESI,† Section S4.1).

We synthesized the peptide segment 9 containing an N-terminal 5-oxaproline by Fmoc-SPPS on resin with 0.3 mmol gram^−1^ followed by TFA cleavage from resin. After purification by preparative RP-HPLC we obtained 160 mg of purified peptide segment 9 in 20% yield (see ESI,† Section 4.3).

With pure segments 8a and 9 in hand, we performed KAHA ligation between α-ketoacid segment 8a (1.0 equiv., 20 mM), with 5-oxaproline segment 9 (1.5 equiv., 30 mM) in 9 : 1 (v/v) DMSO/water. The acidic reaction conditions of KAHA ligation and Gly–Ser *O*-isoacyl moieties were beneficial for solubilizing 8a. The ligation product 10a, containing three ester bonds was formed in 30 h (Fig. 4B). We performed *in situ O* to *N*-acyl shift by diluting the ligation mixture with 10-fold volume of 6 M guanidine hydrochloride (Gdn·HCl), adjusting the pH to 9.6, and stirring at room temperature for another 4 h. A significant retention time shift was observed in analytical RP-HPLC and the rearranged peptide 11a was purified by preparative RP-HPLC and 26 mg of 11a obtained in 54% overall yield after two steps. Hydrolyzed products of the ester bonds were not observed.

The cysteine Acm deprotection of 11a was performed by dissolving peptide in 1% AgOAc in 50% aqueous AcOH at 45 °C for 2 h. The resulting reduced peptide 12a was purified RP-HPLC and isolated in 65% yield.

### Oxidative folding of EPFs

With the reduced EPFs 1a, 6a, and 12a in hand, we optimized the folding conditions in two sequential steps. First, the reduced EPFs were dissolved (0.5 mM concentration) in denaturing buffer (6 M Gdn·HCl, 0.1 M tris(hydroxymethyl)amine hydrochloride (Tris·HCl) pH 6.8) at room temperature open to air. After 1 h, the denatured EPFs were diluted 8-fold with folding buffer (60 μM, final concentration). Different pH values of folding buffer and additives were tested. The best results were obtained by using the following refolding buffer: 1 M Gdn·HCl, 5.0 mM reduced glutathione, and 2.5 mM oxidized glutathione, pH 8.0 for EPFL9; pH 7.0 for EPF2; pH 8.0 for EPF1, at 4 °C for 30 h on a shaker. After purification using preparative RP-HPLC we isolated folded EPFL9 protein 2a (58% isolated yield), EPF2 protein 7a (55% isolated yield), and EPF1 protein 13a (55% isolated yield). The identity was confirmed by ESI-HRMS analysis.

### Bioactivity of synthetic EPFs

**Fig. 5:**
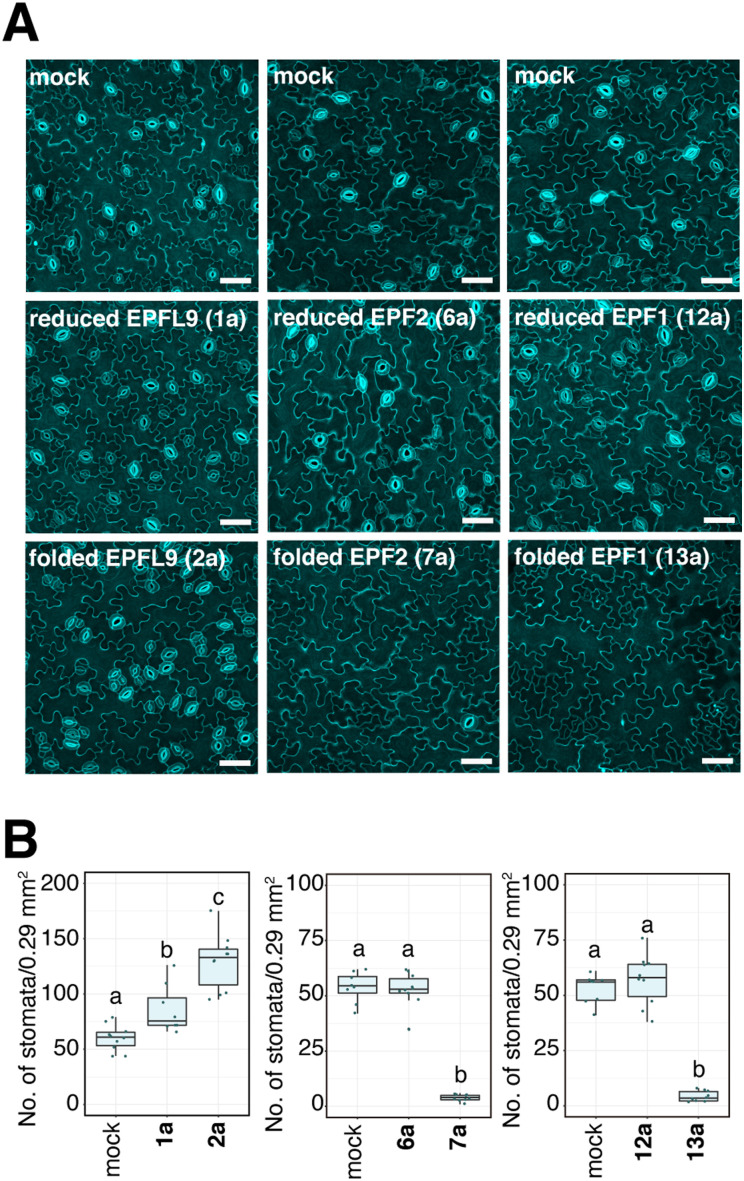
Bioactivity of synthetic EPFs on stomatal formation: (A) representative confocal images of cotyledon abaxial epidermis from the 7-day-old Arabidopsis wild type Col-0 seedlings treated with mock (top), reduced EPFs (middle) or folded EPFs (bottom). Scale bar = 50 μm. (B) Quantitative analysis of the number of stomata shown as a box plot. Dots, individual data points. Median values are shown as lines in the boxplot. ANOVA after Tukey's HSD test was performed for comparison of samples treated with the mock and each peptide. Number of leaves analyzed, *n* = 8, 10, 10, 8, 10, 9, 10, 8, 10 for treatment with mock (for EPFL9), reduced EPFL9 1a, folded EPFL9 2a, mock (for EPF2), reduced EPF2 6a, folded EPF2 7a, mock (for EPF1), reduced EPF1 12a, folded EPF1 13a, respectively. Letters indicate significant difference (*P* < 0.05).

We evaluated the bioactivity of our chemically synthesized EPFs on Arabidopsis wild type plants. It is known that EPFL9 stimulates stomatal development, while EPF2 and EPF1 inhibit it.^58–61^ As shown in Fig. 5, the stomatal density of cotyledons treated with reduced EPFL9 1a and folded EPFL9 2a were 1.4-fold and 2.1-fold higher than those for mock, respectively. When treated with synthetic folded EPF2 7a and folded EPF1 13a, the numbers of stomata in leaves were significantly reduced (7.2% and 8.1% compared with each mock treatment). In contrast, reduced EPF2 6a or EPF1 12a did not affect stomatal numbers (Fig. 5). These results indicate that synthetic EPFs have activity on stomatal development as the activities are comparable with previous reports.^63,81,82^

### Synthesis of EPFs with a KAT ligation handle

In order to introduce reporter groups onto EPF with site-specifically and acquire suitable probes to visualize dynamics of EPF proteins *in vivo*, we incorporated a hydroxylamine at N-terminus of EPFs, which allows introduction of fluorescent dyes with site selective manner to folded proteins.

The reduced EPFL9 1b was synthesized by standard Fmoc SPPS and introduction of ornithine hydroxylamine with photo labile protecting group (Orn HA) HA at N-terminus. We resynthesized the segments 3 and 8 with KAT ligation handle by introducing Orn HA, which we reported previously^31^ at the N-terminus during Fmoc SPPS. The thioester segment 3b and α-ketoacid segment 8b were obtained in 46% and 16% yield, respectively. NCL of thioester segment 3b and 4 gave Acm protected ligation product 5b in 68% yield. The KAHA ligation reaction was performed between 8b and 9 using previously established EPF1 ligation conditions, followed by *O* to *N*-acyl shift. The Acm protected EPF1 11b was isolated in 62% yield (over two steps). The reduced EPF2 6b and EPF1 12b were obtained in 72% and 70% yield, respectively. The reduced proteins 1b, 6b and 12b were folded using previously optimized folding conditions.

### Fluorescent labelling of EPF proteins by KAT ligation

**Fig. 6:**
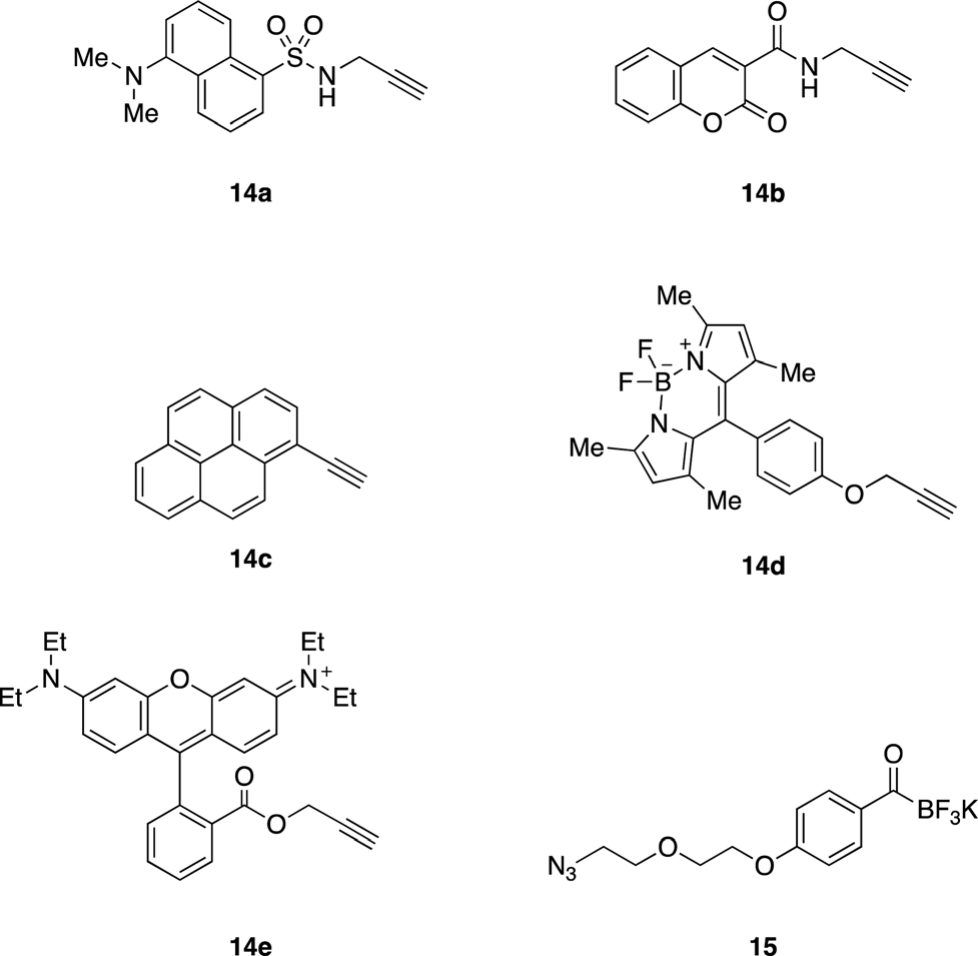
Alkyne containing dyes 14a–e and azido functionalized KAT 15.

We envisioned visualizing the subcellular localization of EPFs by fluorescent labeling of EPFs and fluorescent microscopy.^83–85^ In order to find suitable fluorescent dye, we synthesized KATs 16a–e from alkyne containing dyes 14a–e (Fig. 6) with azido functionalized KAT 15 through azide–alkyne cycloaddition in 55–81% yields (see ESI,† Section S6).

**Scheme 1:**
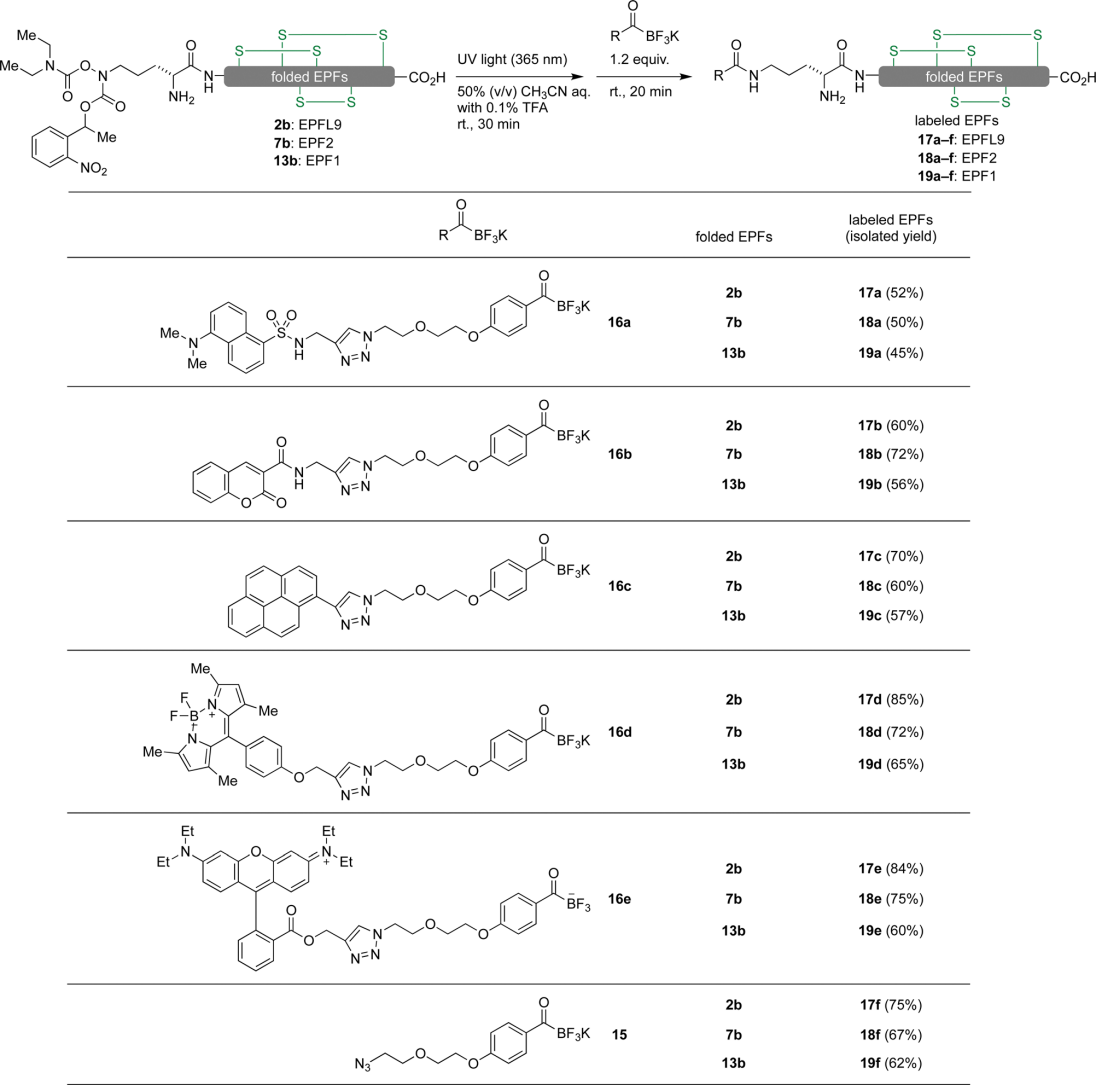
Fluorescent labelling of EPFs by KAT ligation.

Initially, KAT ligation of 2b and dansyl KAT 16a was examined. UV light (365 nm) irradiation to a mixture of 2b (50 μM, 1.0 equiv.) and 16a (75 μM, 1.5 equiv.) in 50% aqueous acetonitrile mixture with 0.1% TFA only gave a trace amount of the desired product in 20 min; the majority of 16a decomposed and became unreactive towards the hydroxylamine. To prevent decomposition of the fluorophore, we first irradiated a solution of 2b with 365 nm UV light for 30 minutes. After the complete removal of photo-labile protecting group analyzed by analytical RP-HPLC, dansyl KAT 16a (1.2 equiv.) was added to the reaction mixture at room temperature. We were pleased to see that the reaction proceeded cleanly without decomposition of 16a after 20 minutes and isolated the dansyl labeled EPFL9 17a in 52% yield (Scheme 1). Using these conditions, we functionalized folded EPF proteins, 2b, 7b, 13b to a variety of dye-labeled folded EPF proteins 17a–f, 18a–f, and 19a–f in 45–85% yields using KATs 16a–e (Scheme 1).

### Bioassay data of dye-labeled EPFL9 for stomatal development in *Arabidopsis* plants

**Fig. 7:**
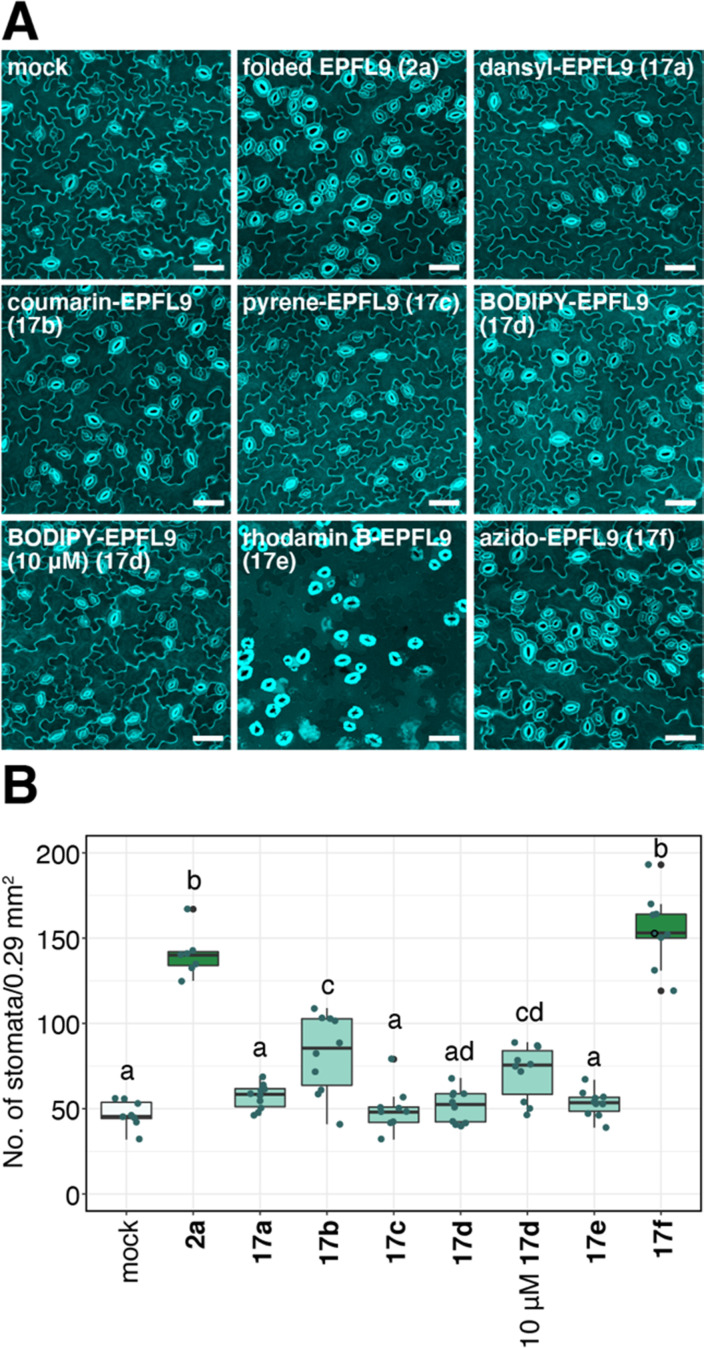
Fluorescent labeled EPFL9 retain various degrees of bioactivity. (A) Representative confocal images of cotyledon abaxial epidermis from the 7-day-old Arabidopsis wild type Col-0 seedlings treated with mock, 5 μM folded EPFL9 2a, 5 μM azido-EPFL9 17f and 5 μM fluorescence-labeled EPFL9s 17a–e. For the BODIPY-EPFL9 17d treatment, the image for 10 μM treatment is also shown. Scale bar = 50 μm. (B) Quantitative analysis of the number of stomata shown as a box plot. Dots, individual data points. Median values are shown as lines in the boxplot. ANOVA after Tukey's HSD test was performed for comparison of samples treated with the mock and each peptide. Number of leaves analyzed, *n* = 8, 7, 9, 10, 10, 10, 10, 9, 10 for treatment with mock, 5 μM folded EPFL9 2a, 5 μM azido-EPFL9 17f, 5 μM coumarin-EPFL9 17b, 5 μM BODIPY-EPFL9 17d, 10 μM BODIPY-EPFL9 17d, 5 μM dansyl-EPFL9 17a, 5 μM pyrene-EPFL9 17c, and 5 μM rhodamine B-EPFL9 17e, respectively. Letters indicate significant difference (*P* < 0.05).

We evaluated the bioactivity of dye-labeled EPFL9 **17**a–e and azide variant 17f. As shown in Fig. 7, 17f, the control for dye-labeled EPFL9 (in which similar reaction procedure was conducted without adding coupling dyes) showed stomata-inducing activity as strong as non-labeled folded EPFL9 2a. Most EPFL9 variants (17a–e) except coumarin-EPFL9 17b showed reduced bioactivity (Fig. 7). When leaves were treated with 10 μM BODIPY-EPFL9 17d, the stomatal number was significantly elevated in comparison to those of the mock-treated control, indicating that BODIPY-EPFL9 17d retains its bioactivity (Fig. 7). Non-conjugated fluorophores alone did not impart any activity on stomatal development (ESI,† Fig. S1). It is likely that coupling of fluorescent dyes might affect EPFL9 structures, which reflect various degrees of their bioactivities.

### BODIPY-EPFL9 is incorporated into *Arabidopsis* leaves

**Fig. 8:**
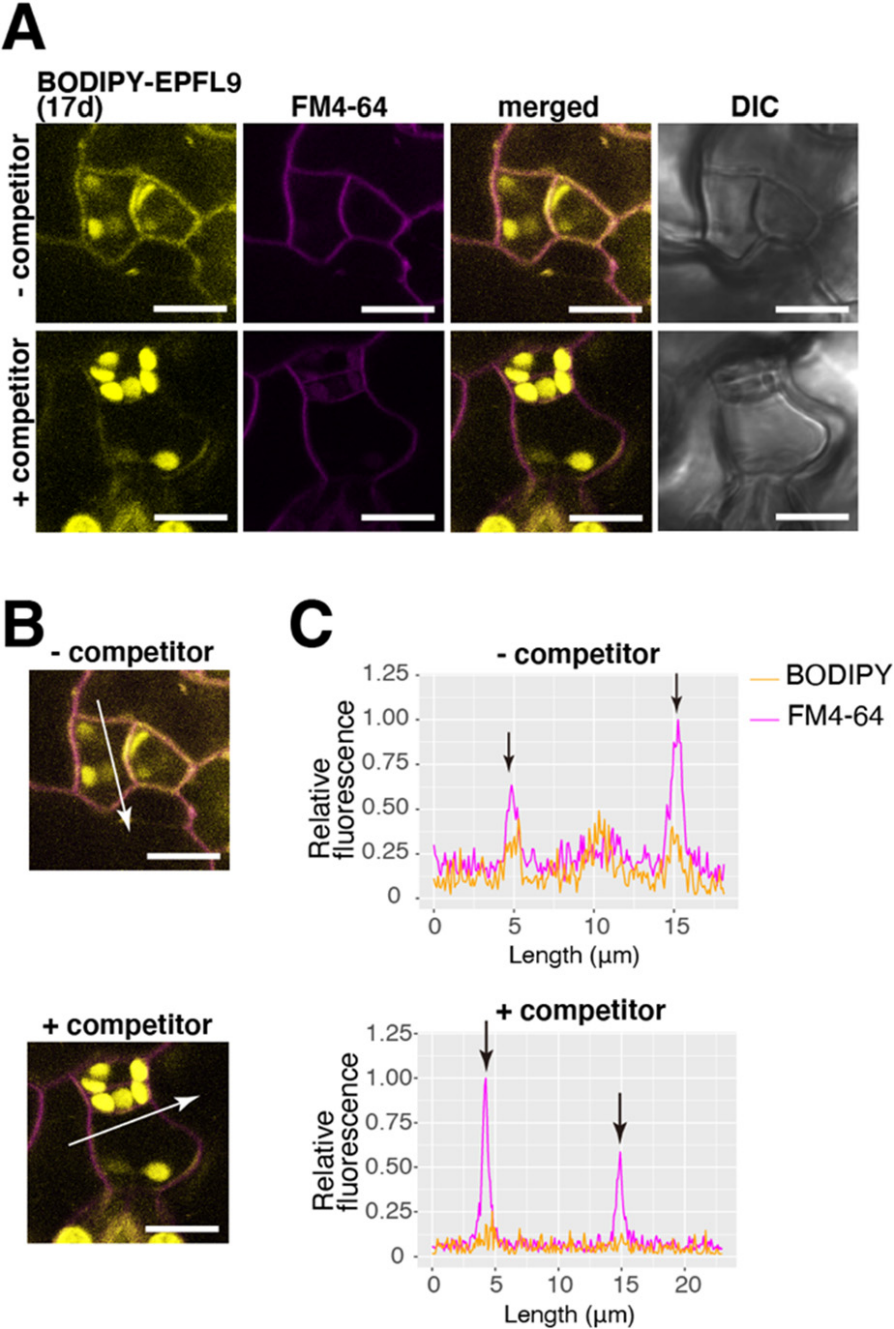
BODIPY-EPFL9 17d is localized at the plasma membrane and competed by EPFL9 2a. (A) Representative confocal images of BODIPY (yellow) and FM4-64 (magenta) in the abaxial epidermis of cotyledons from the 7-day-old Arabidopsis Col-0 seedlings. The seedlings were treated with 0.6 μM FM4-64 and 6 nM BODIPY-EPFL9 17d with or without 6 nM non-labeled folded EPFL9 2a for 10 min. Scale bar = 20 μm. (B) Merged images of BODIPY and FM4-64. Scale bar = 10 μm. (C) Line plots of BODIPY (yellow) and FM4-64 (magenta) fluorescence on 1-pixel-thickness of arrow indicated in (B). Arrows indicate FM4-64 fluorescence staining the plasma membrane.

EPF peptides interact with the receptor-like kinases ERECTA-family proteins localized at the plasma membrane^81^ and initiates stomatal signaling. Since BODIPY-EPFL9 17d retains bioactivity (Fig. 7), we sought to visualize the localization of fluorescence-labeled EPFL9 in the cotyledon epidermis. For this purpose, *Arabidopsis* seedlings were co-treated with BODIPY-EPFL9 17d and FM4-64 (a styryl dye that stains plasma membrane).^86^ Strong BODIPY signals (yellow) were detected in the plasma membrane of stomatal precursors, co-localizing with FM4-64 signals (magenta) (Fig. 8A). Quantification of line slicing showed two BODIPY/FM4-64 peaks corresponding to the plasma membrane, with signal ratio of 0.55 and 0.41, respectively (Fig. 8B and C).

In contrast, when incubating BODIPY-EPFL9 17d and FM4-64 in the presence of non-labeled folded EPFL9 2a as a competitor, no clear peaks for BODIPY signals were detected at the plasma membrane (Fig. 8A). The BODIPY/FM4-64 signal ratios at the plasma membrane were declined to 0.18 and 0.14, much lower than those incubated without non-labeled EPFL9 2a (Fig. 8B and C). These results indicate that BODIPY-EPFL9 17d was incorporated into the *Arabidopsis* epidermis. The observation that BODIPY-EPFL9 17d competes with the non-labeled folded EPFL9 2a suggests the specificity and reversible, transient nature of peptide binding. It is known that EPF2 and EPFL9 competitively bind to the receptor to fine-tune stomatal patterning,^87^ and that the receptor dynamically changes its subcellular localization upon perceiving different EPF/EPFL peptides.^88^ In the future, multiple visualization of ERECTA-family receptors and multiple fluorescence-labeled EPF peptides could help further understanding the behavior of their interactions.

## Conclusions

In summary, EPFs were chemically synthesized by chemoselective amide bond forming reactions – EPF2 by NCL and EPF1 by KAHA ligation. Chemically synthesized EPFs showed the expected bioactivities on stomatal development in *Arabidopsis* plants. Late-stage attachment of fluorescent dyes to folded proteins were achieved by chemoselective amide bond forming reaction, KAT ligation. This efficient synthesis of the protein variants through the folded EPFs bearing hydroxylamine as a key intermediate of derivatization enabled us to identify that BODIPY-labeled EPFL9 17d maintains the protein activity and suitable for bioimaging study. Localization of EPFL9 to the plasma membrane was visualized by fluorescent microscopy.

The choice of fluorescent dyes is important for bioimaging studies. The late-stage functionalization strategy enabled us to prepare a variety of protein and choose a suitable variant for bioimaging.

Having now the fluorescent labeled EPF proteins, we will pursue visualization of signal perception and transduction in plants. We anticipate that this late-stage functionalization strategy of synthetic proteins should be applicable to other CRPs.

## Author contributions

S. O. conceived of the idea. N. K. synthesized the EPF proteins, dye-KATs, and the labeled EPFs. A. N. and H. E. performed the stomatal development assays. A. N. and A. S. carried out the bioimaging studies. N. K., A. N., K. U. T., J. W. B. and S. O. designed the experiments and analyzed the data. K. U. T. and S. O. obtained the funding for this research project. N. K., A. N. and S. O. wrote the manuscript with help from all authors.

## Conflicts of interest

The authors declare no conflict of interest.

## Supplementary Material

CB-003-D2CB00155A-s001
