# Supplementary material for: Chemical synthesis of the EPF-family of plant cysteine-rich proteins and late-stage dye attachment by chemoselective amide-forming ligations
Source: RSC Chem Biol. 2022 Oct 19;3(12):1422–31. doi: 10.1039/d2cb00155a (PMC9709926; doi:10.1039/d2cb00155a)
Supplement: CB-003-D2CB00155A-s001 [file CB-003-D2CB00155A-s001.pdf]

Electronic Supplementary Material (ESI) for RSC Chemical Biology.
This journal is © The Royal Society of Chemistry 2022

Supporting Information

Chemical Synthesis of the EPF-Family of Plant Cysteine-Rich Proteins and Late-Stage Dye Attachment by Chemoselective Amide-Forming Ligations

Nandarapu Kumarswamyreddy[1,2 +], Ayami Nakagawa[1 +], Hitoshi Endo[1], Akie Shimotohno[1], Keiko U. Torii[1,3], Jeffrey W. Bode[1,4], and Shunsuke Oishi[1]*

[1]Institute of Transformative Bio-Molecules (WPI–ITbM), Nagoya University, Chikusa Nagoya 464-8602, Japan.

[2]Department of Chemistry, Indian Institute of Technology Tirupati, Tirupati-517619, Andhra Pradesh, India.

[3]Howard Hughes Medical Institute and Department of Molecular Biosciences, The University of Texas at Austin, Austin, TX 78712 USA

[4]Laboratorium für Organische Chemie, Department of Chemistry and Applied Biosciences, ETH Zürich, Zürich 8093, Switzerland.

[+]These authors contributed equally to this work.

Table of Contents

1. General methods 2
2. Chemical synthesis of EPFL9 proteins 5
3. Chemical synthesis of cysteine protected EPF2 proteins 7
4. Chemical synthesis of cysteine protected EPF1 proteins 12
5. Deprotection of Acm group and protein folding 17
6. Synthesis of potassium acyltrifluoroborates (KATs) compounds 24
7. Protein functionalization by KAT ligation 42
8. Bioassay 55
9. References 57

1. General methods

1.1 Reagents and solvents and abbreviations

Fmoc-amino acids with suitable side-chain protecting groups, HCTU (*O*-(1*H*-6-Chlorobenzotriazol-1-yl)- *N*,*N*,*N*',*N*'-tetramethyluroniumhexafluorophosphate) and HATU(1-[Bis(dimethylamino)methylene]-1H-1,2,3-triazole[4,5-b]pyridinium 3-oxid hexafluorophosphate) were purchased from Merck KGaA (Darmstadt, Germany). HPLC grade CH_3CN from Kanto Chemical Co., Inc. (Tokyo, Japan) and FUJIFILM Wako Pure Chemical Corporation (Osaka, Japan) was used for analytical and preparative HPLC purification. DMF from FUJIFILM Wako Pure Chemical Corporation was directly used without further purification for solid phase peptide synthesis. Protected Fmoc-Val-α-ketoacid,[1] Boc-(*S*)-5-oxaproline,[2] and Fmoc-Orn HA[3] were prepared as previously reported by our group. Gly-Ser isoacyl dipeptide was prepared according to reported procedure.[4] All other chemicals were of the highest-grade commercially available and used as received. Merck KGaA (Darmstadt, Germany), Kanto Chemical Co., Inc. (Tokyo, Japan), FUJIFILM Wako Pure Chemical Corporation (Osaka, Japan), and Tokyo Chemical Industry Co., Ltd. (Tokyo, Japan).

List of the building blocks used for this work:

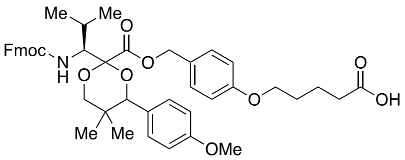

Protected Fmoc-Val-α-ketoacid

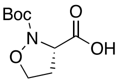

Boc-(*S*)-5-oxaproline
(Boc-Opr)

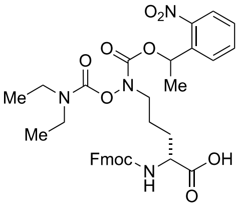

Protected Fmoc-Ornithine hydroxylamine
(Fmoc-Orn HA)

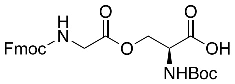

Gly-Ser isoacyl dipeptide

List of abbreviations used:

Acm: acetamidomethyl

Boc-Opr: (*S*)-2-(tert-butoxycarbonyl) isoxazolidine-3-carboxylic acid

Boc: *tert*-butoxycarbonyl

COMU: (1-cyano-2-ethoxy-2-oxo-ethylidenaminooxy)-dimethylaminomorpholinocarbenium hexafluorophosphate

DIC: *N*,*N*'-diisopropylcarbodiimide

DMF: *N*,*N*-dimethylformamide

DMSO: dimethylsulfoxide

DODT: 2,2-(ethylenedioxy)diethanethiol

DTT: dithiothreitol

Fmoc: 9-fluorenylmethyloxycarbonyl

Gdn•HCl: guanidine hydrochloride

GSH: glutathione

GSSG: glutathione disulfide

HATU: *O*-(7-azabenzotriazol-1-yl)-*N,N,N',N'*-tetramethyluronium hexafluorophosphate

HCTU: *O*-(1*H*-6-chlorobenzotriazol-1-yl)-*N,N,N',N'*-tetramethyluronium hexafluorophosphate

HOBt: hydroxybenzotriazole

NMM: *N*-methylmorpholine

NMP: *N*-methyl-2-pyrrolidone

SPPS: solid phase peptide synthesis

TCEP: tris(2-carboxyethyl)phosphine

TFA: trifluoroacetic acid

TIPS: triisopropylsilane

Trt: trityl group

1.2 Peptide synthesis

a) Solid phase peptide synthesis (SPPS)

Peptides were synthesized on a CS Bio 136X synthesizer using Fmoc SPPS chemistry. The following Fmoc amino acids with side-chain protecting groups were used: Fmoc-Ala-OH, Fmoc-Arg(Pbf)-OH, Fmoc-Asn(Trt)-OH, Fmoc-Asp(O*t*Bu)-OH, Fmoc-Cys(Acm)-OH Fmoc-Cys(Trt)-OH, Fmoc-Gln(Trt)-OH, Fmoc-Glu(O*t*Bu)-OH, Fmoc-Gly-OH, Fmoc-His(1-Trt)-OH, Fmoc-Ile-OH, Fmoc-Leu-OH, Fmoc-Lys(Boc)-OH, Fmoc-Phe-OH, Fmoc-Pro-OH, Fmoc-Ser(*t*Bu)-OH, Fmoc-Thr(*t*Bu)-OH, Fmoc-Trp(Boc)-OH, Fmoc-Tyr(*t*Bu)-OH, Fmoc-Val-OH, Fmoc-Nle-OH. SPPS was performed on aminomethyl polystyrene resin or HMPB-ChemMatrix resin or 2-chlorotriltyl polystyrene resin. Manual loading of the first amino acid residue onto the resin and subsequent Fmoc-SPPS followed established standard protocols. A summary of the utilized synthesis protocols: Fmoc-deprotections were performed with 20% piperidine in DMF (8 min ×2). Couplings were performed with Fmoc-amino acid (4.0 equiv relative to resin substitution), HCTU (3.8 equiv) and NMM (8.0 equiv) in DMF for 60 min. If required, the coupling step was repeated (double coupling) and LiCl washes (0.8 M LiCl in DMF) were performed before Fmoc-deprotection and coupling. After coupling, unreacted free amine was capped by treatment with 20% acetic anhydride and 10% NMM in DMF for 10 min. Amino acid residues prone to epimerization such as cysteine were coupled using preformed HOBt esters. In a typical procedure,

Fmoc-Cys(Acm)-OH (4.0 equiv relative to resin loading) was dissolved in DMF, and HOBt (4.0 equiv) and DIC (4.0 equiv) were added. The mixture was added to the resin and allowed to react for 2 h.

b) Manual coupling of special amino acids

Protected Fmoc-Val-α-ketoacid, Boc-(S)-5-oxaproline, Fmoc-Orn HA, and Gly-Ser isoacyl dipeptide were coupled manually. The monomer (1.5 equiv) was dissolved in a minimal amount of DMF (minimal concentration of monomer: 0.1 M), HATU (1.5 equiv) and NMM (3.0 equiv) were added. After a brief period of preactivation (2 min), the solution was added to the resin and allowed to react for 2 h. If required, the coupling was repeated with 1.0 equiv of monomers, 1.0 equiv of HATU, and 2.0 equiv of NMM.

c) Mutations and protecting groups

Norleucine Substitution: All methionine residues (Met) were substituted by norleucine (Nle) residues in the protein sequence, to avoid oxidation while handling, storage, and refolding.

1.3 General HPLC analysis and purification

Peptides and protein segments were analyzed and purified by reverse phase high performance liquid chromatography (RP-HPLC) on Jasco analytical and preparative instruments equipped with dual pumps, a mixer, an in-line degasser, and variable wavelength UV detector (simultaneous monitoring of the eluent at 220 nm, 254 nm, and 301 nm) or on a Gilson preparative instrument fitted with a 10 mL injection loop. If required, the columns were preheated using a column heater or a water bath. The mobile phase for RP-HPLC were Milli-Q water containing 0.1% TFA and HPLC grade CH_3CN containing 0.1% TFA. In the described HPLC analysis and purifications, TFA was always used as solvent modifier.

Analytical RP-HPLC: Analytical HPLC was performed on a Shiseido Capcell Pak MG-II (5 μm, 120 Å pore size, 4.6 mm I.D. × 250 mm), or on a Phenomenex Jupiter C18 (5 μm, 300 Å pore size, 4.6 mm I.D. × 250 mm), or on Phenomenex Jupiter C4 (5 μm, 300 Å pore size, 4.6 mm I.D. × 250 mm) at a flow rate of 1 mL/min.

Preparative RP-HPLC: Preparative HPLC was performed on a Shiseido Capcell pak C18 UG80 (50 mm I.D. × 250 mm), on a Phenomenex Jupiter C18 (5 μm, 300 Å pore size, 30 mm I.D. × 250 mm), on a Phenomenex Jupiter C18 (5 μm, 300 Å pore size, 21.2 mm I.D. × 250 mm), on a

Phenomenex Jupiter C4 (5 µm, 300 Å pore size, 30 mm I.D. × 250 mm), or on a Phenomenex Jupiter C4 (5 µm, 300 Å pore size, 21.2 mm I.D. × 250 mm). The following type of method was used: the column was pre-equilibrated at starting solvent composition for typically 10 min. After injection of the sample, the solvent composition was run to the final solvent composition (e.g., 50% CH_3CN). After the gradient run time, the solvent composition was changed to 95% CH_3CN within 1 min and the column was flushed for 5–7 min. Within 1 min, the solvent composition was changed to 10% CH_3CN and the run ended. For the sake of simplicity, only the gradient time, the starting and end composition of the eluent will be stated at the individual experiments, although all experiments included the full cycle as described above.

1.4 Characterization

MALDI-MS data were obtained on a Bruker Microflex MALTI-TOF spectrometer using 4-hydroxy-α-cyanocinnamic acid as the matrix. High-resolution mass spectra were recorded by the Molecular Structure Center at ITbM, Nagoya University on Thermo Scientific™Exactive™ Plus Orbitratp Mass Spectrometer.

2. Chemical synthesis of EPFL9 proteins

2.1 Synthesis of reduced EPFL9 protein 1a

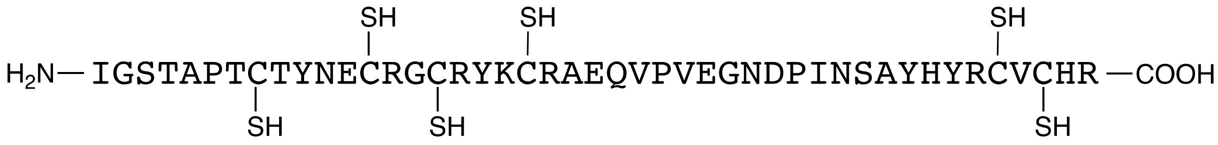

The reduced EPFL9 **1a** was synthesized on the 2-chloro trityl chloride resin preloaded with Fmoc-Arg-OH (0.5 g, 0.39 mmol loading capacity). After automated SPPS, the resin was washed several times with DMF followed by CH_2Cl_2, dried and subjected to cleavage using a mixture of 95:2.5:2.5 TFA:TIPS:H_2O (20 mL/g resin) for 2 h at room temperature. The crude TFA solution was separated from the resin by filtration and the filtrate was concentrated under reduced pressure. The residue was triturated with cooled Et_2O (ca. 25 mL/g resin), centrifuged and the supernatant was removed by decantation. This trituration/washing step was repeated twice (peptide precipitating out). The crude peptide was purified by preparative RP-HPLC using Phenomenex Jupiter® C18 column (5 µm, 300 Å pore size, 30 mm I.D. × 250 mm) with a gradient of 20–70% CH_3CN (with 0.1% TFA) in 30 min with 20 mL/min flow rate. The fractions containing product

were pooled and lyophilized to obtained 358 mg of reduced EPFL9 **1a**. The m/z calculated for **1a** $C_{213}H_{338}N_{69}O_{67}S_6$ $[M+7H]^{7+}$: 732.3350 Da, measured: 732.3338 Da.

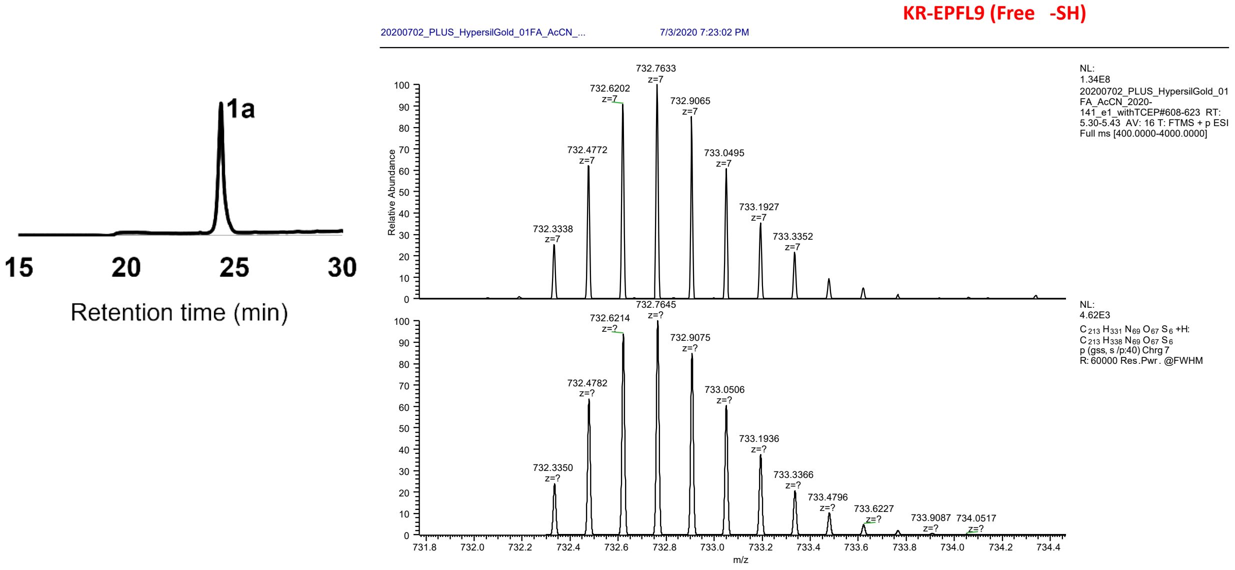

2.2 Synthesis of reduced EPFL9 protein 1b

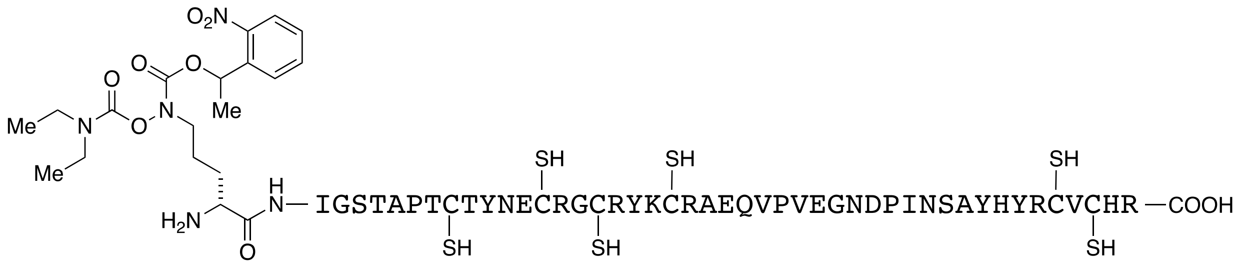

The reduced EPFL9 **1b** was synthesized on the 2-chloro trityl chloride resin preloaded with Fmoc-Arg-OH (0.5 g, 0.39 mmol loading capacity). After automated SPPS, the resin was washed several times with DMF followed by CH_2Cl_2, dried and subjected to cleavage using a mixture of 95:2.5:2.5 TFA:TIPS:H_2O (20 mL/g resin) for 2 h at room temperature. The crude TFA solution was separated from the resin by filtration and the filtrate was concentrated under reduced pressure. The residue was triturated with cooled Et_2O (ca. 25 mL/g resin), centrifuged and the supernatant was removed by decantation. This trituration/washing step was repeated twice (peptide precipitating out). The crude was purified by preparative RP-HPLC using Phenomenex Jupiter® C18 column (5 µm, 300 Å pore size, 30 mm I.D. × 250 mm) with a gradient of 20–70% CH_3CN (with 0.1% TFA) in 30 min with 20 mL/min flow rate. The fractions containing product were pooled and lyophilized to obtained 207 mg of reduced EPFL9 **1b**. The m/z calculated for **1b** $C_{232}H_{364}N_{73}O_{74}S_6$ $[M+7H]^{7+}$: 792.6464 Da, measured: 792.6448 Da.

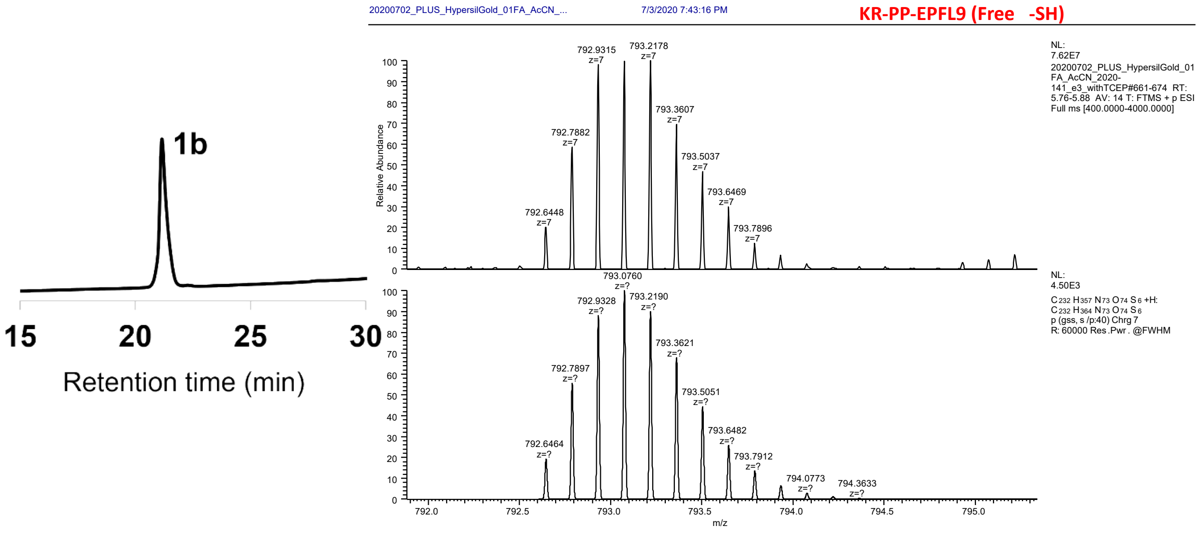

3. Chemical synthesis of cysteine protected EPF2 proteins

3.1 Synthesis of Cys(Acm) protected thioester peptide 3a

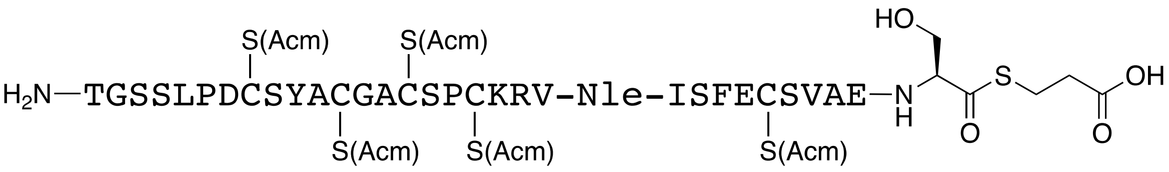

The peptide thioester **3a** was synthesized on 2-chlorotrityl hydrazine polystyrene resin prepared by reported procedure.[5] The Fmoc-Ser(Trt)-OH (1.0 equiv) was coupled manually to the resin using HATU (1.0 equiv) and NMM (2.0 equiv) in anhydrous DMF at room temperature for 2 h and then the loading capacity was identified. After automated Fmoc SPPS, the resin was washed several times with DMF followed by CH_2Cl_2, dried and subjected to cleavage using a mixture of 95:2.5:2.5 TFA:TIPS:H_2O (20 mL/g resin) for 2 h at room temperature. The crude TFA solution was separated from the resin by filtration and the filtrate was concentrated under reduced pressure. The residue was triturated with cooled Et_2O (ca. 25 mL/g resin), centrifuged and the supernatant was removed by decantation. This trituration/washing step was repeated twice. The crude peptide hydrazide was dried and dissolved in aqueous CH_3CN with 0.1% TFA for RP-HPLC purification.

The peptide hydrazide (1.0 equiv) was dissolved in phosphate buffer containing 0.2 M NaH_2PO_4, 6 M Gdn•HCl, pH 3.0. The mixture was cooled to –15 °C (ice + NaCl) and maintained for 20 min. The aqueous $NaNO_2$ (10 equiv) was added dropwise and stirred at –15 °C for another 30 min. The β-mercaptopropionic acid (25 equiv) was added to the reaction mixture at –15 °C and slowly bring the reaction mixture to room temperature. The pH of the reaction mixture was adjusted to 6.8–7.0 and maintained until the reaction was complete. The reaction progress was

monitored by analytical RP-HPLC. The crude peptide thioester **3a** was purified by preparative RP-HPLC using Phenomenex Jupiter® C4 column (5 µm, 300 Å pore size, 30 mm I.D. × 250 mm) with a gradient of 20–70% CH_3CN (with 0.1% TFA) in 30 min with 20 mL/min flow rate. The fractions containing desired product were pooled and lyophilized to give **3a** (62 mg, 60% yield corresponding from 100 mg of hydrazide peptide). The m/z calculated for **3a** $C_{153}H_{248}N_{41}O_{54}S_6$ $[M+3H]^{3+}$: 1238.5409 Da, measured: 1238.5476 Da.

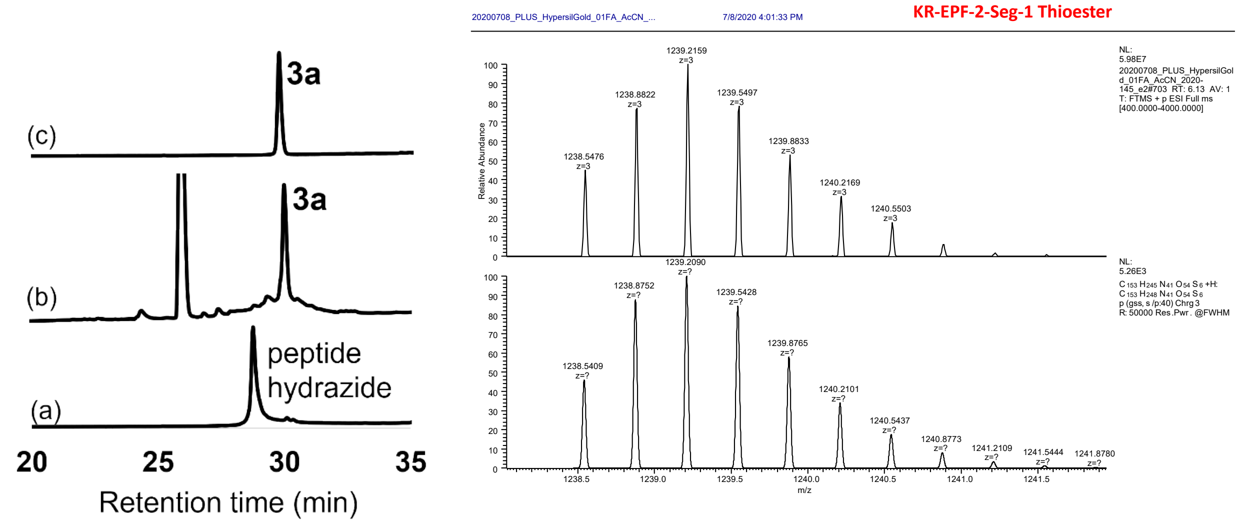

a) Thioester formation at 0 h; b) Thioester formation at 30 min; c) Purified peptide thioester **3a**

3.2 Synthesis of Cys(Acm) protected thioester peptide 3b

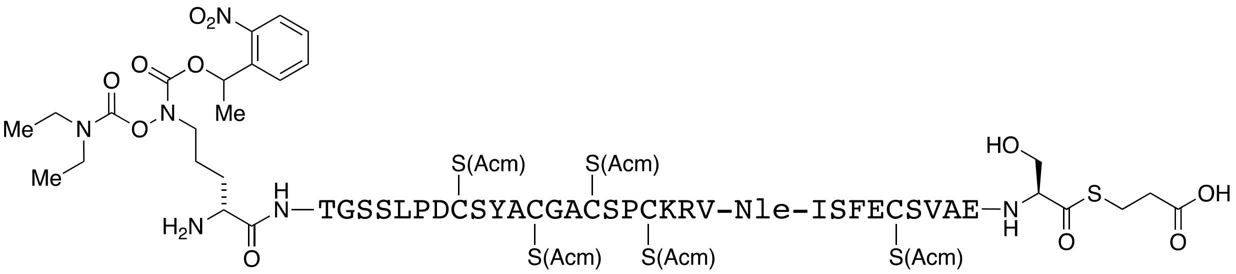

The peptide thioester **3b** was synthesized on 2-chlorotrityl hydrazine polystyrene resin prepared by reported procedure.[4] The Fmoc-Ser(Trt)-OH (1.0 equiv) was coupled manually to the resin using HATU (1.0 equiv) and NMM (2.0 equiv) in anhydrous DMF at room temperature for 2 h and then the loading capacity was identified. After automated Fmoc SPPS, Fmoc-Orn HA (1.1 equiv, 0.33 mmol) was manually coupled using HATU (1.1 equiv 0.33 mmol) and NMM (2.2 equiv, 0.66 mmol) in anhydrous DMF for 2 h at room temperature. The resin was washed several times with DMF followed by CH_2Cl_2, dried and subjected to cleavage using a mixture of 95:2.5:2.5 TFA:TIPS:H_2O (20 mL/g resin) for 2 h at room temperature. The crude TFA solution

was separated from the resin by filtration and the filtrate was concentrated under reduced pressure. The residue was triturated with cooled Et_2O (ca. 25 mL/g resin), centrifuged and the supernatant was removed by decantation. This trituration/washing step was repeated twice. The crude peptide hydrazide was dried and dissolved in aqueous CH_3CN with 0.1% TFA for RP-HPLC purification.

The peptide hydrazide (1.0 equiv) was dissolved in phosphate buffer containing 0.2 M NaH_2PO_4, 6 M Gdn•HCl pH 3.0. The mixture was cooled to –15 °C (ice + NaCl) and maintained for 20 min. The aqueous $NaNO_2$ (10 equiv) was added dropwise and stirred at –15 °C for another 30 min. The β-mercaptopropionic acid (25 equiv) was added to the reaction mixture at –15 °C and slowly bring the reaction mixture to room temperature. The pH of the reaction mixture was adjusted to 6.8–7.0 and maintained until the reaction was complete. The reaction progress was monitored by analytical RP-HPLC. The crude peptide thioester **3b** was purified by preparative RP-HPLC using Phenomenex Jupiter® C4 column (5 µm, 300 Å pore size, 30 mm I.D. × 250 mm) with a gradient of 20–70% CH_3CN (with 0.1% TFA) in 30 min with 20 mL/min flow rate. The fractions containing desired product were pooled and lyophilized to give **3b** (45 mg, 46% yield corresponding from 100 mg of hydrazide peptide). The m/z calculated for **3b** $C_{172}H_{274}N_{45}O_{61}S_6$ $[M+3H]^{3+}$: 1379.2677 Da, measured: 1379.2747 Da.

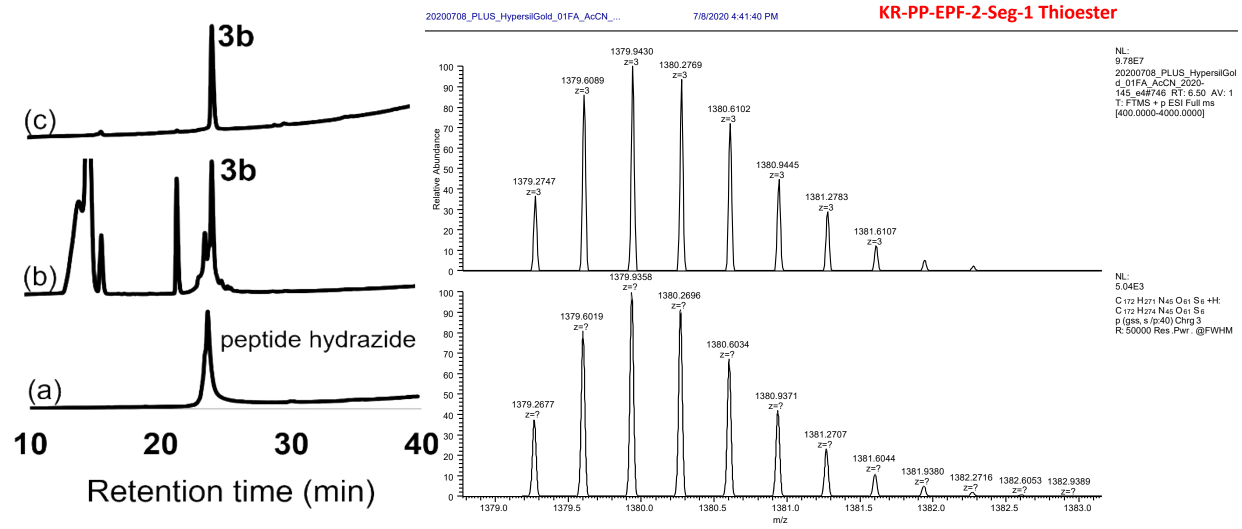

a) Thioester formation at 0 h; b) Thioester formation at 30 min; c) Purified peptide thioester **3b**

3.3 Synthesis of Cys(Acm) protected peptide 4

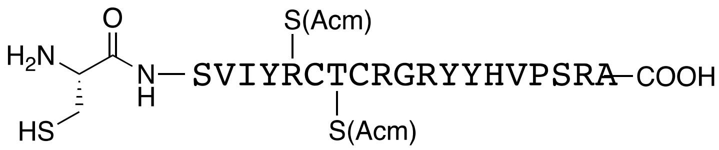

The cysteine peptide **4** was prepared on 2-chloro trityl chloride resin resin preloaded with Fmoc-Ala-OH (0.41 mmol/g loading). After the automated SPPS procedure, the resin was subjected to cleavage using a mixture of 95:2.5:2.5 TFA:TIPS:H_2O (20 mL/g resin) for 2 h at room temperature. The crude TFA solution was separated from the resin by filtration and the filtrate was concentrated under reduced pressure. The residue was triturated with cooled Et_2O (ca. 25 mL/g resin), centrifuged and the supernatant was removed by decantation. This trituration/washing step was repeated twice. The crude peptide was dissolved in aqueous CH_3CN with 0.1% TFA and purified by preparative RP-HPLC using on a Phenomenex Jupiter® C18 column (5 µm, 300 Å pore size, 30 mm I.D. × 250 mm) with a gradient of 20–70% CH_3CN (with 0.1% TFA) in 30 min with 20 mL/min flow rate. The pure product fractions were pooled and lyophilized to give 260 mg of **4** (obtained from 1 g of dried resin after SPPS). The m/z calculated for **4** $C_{108}H_{173}N_{36}O_{29}S_3$ $[M+3H]^{3+}$: 844.7438 Da, measured: 844.7476 Da.

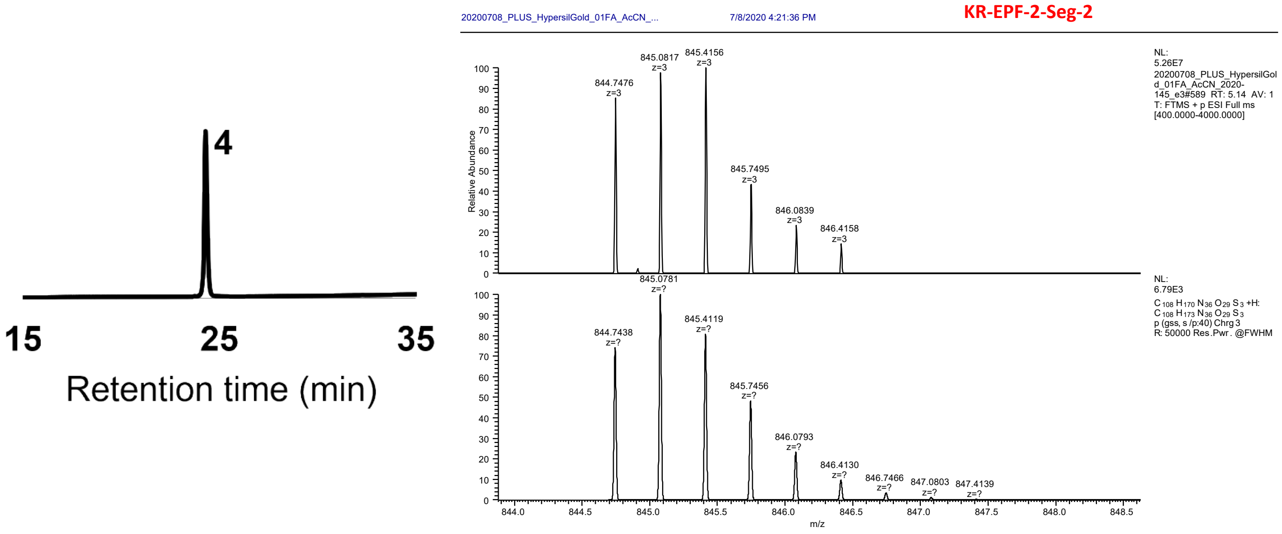

3.4 Synthesis of Cys(Acm) protected EPF2 protein 5a by native chemical ligation

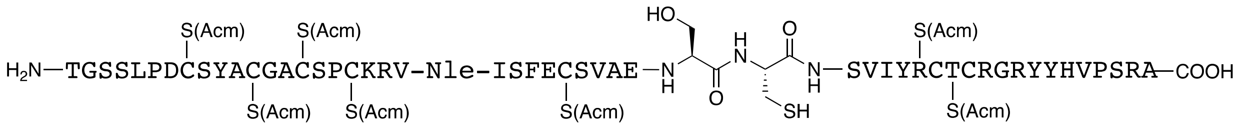

The cysteine peptide **4** (15 mg, 5.9 µM, 1.1 equiv) and the peptide thioester **3a** (20 mg, 5.4 µM, 1.0 equiv) were dissolved in 2.5 mL of ligation buffer containing 6 M Gdn•HCl, 200 mM Na_2HPO_4, 100 mM TCEP, 100 mM sodium ascorbate, 3% (v/v) thiophenol, pH 7.4, and the reaction mixture was stirring at room temperature for 16 h. The progress of the reaction was monitored by analytical RP-HPLC. After completion of the reaction, the crude ligated peptide **5a**

was purified by preparative RP-HPLC using Phenomenex Jupiter® C4 column (5 µm, 300 Å pore size, 30 mm I.D. × 250 mm) with a gradient of 20–80% CH_3CN (with 0.1% TFA) in 30 min with 20 mL/min flow rate. The fractions containing the desired product were pooled and lyophilized to give **5a** (22 mg, 67% yield). The m/z calculated for **5a** $C_{258}H_{414}N_{77}O_{81}S_8$ $[M+5H]^{5+}$: 1228.5676 Da, measured: 1228. 5675 Da.

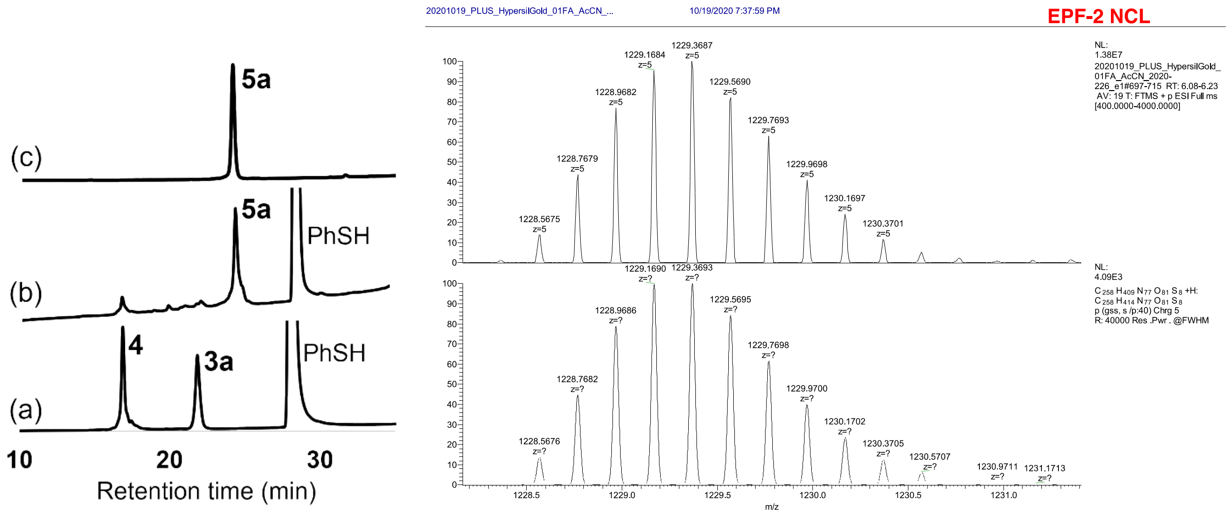

a) NCL at 0 h; b) NCL at 16 h; c) Purified linear protein **5a**

3.5 Synthesis of Cys(Acm) protected EPF2 protein 5b by native chemical ligation

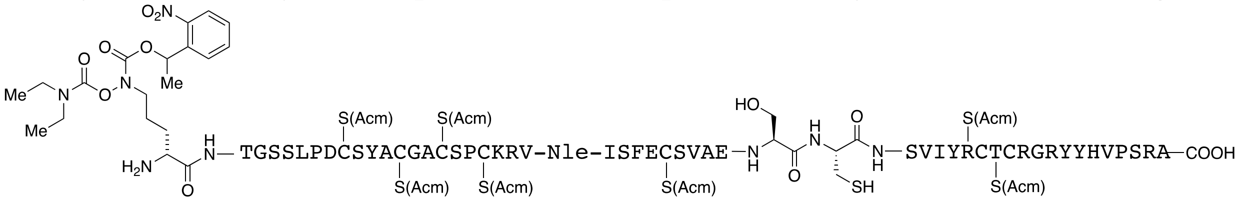

The cysteine peptide **4** (15 mg, 5.9 µM, 1.1 equiv) and the peptide thioester **3b** (20 mg, 4.8 µM, 1.0 equiv) were dissolved in 2.5 mL of ligation buffer containing 6 M Gdn•HCl, 200 mM Na_2HPO_4, 100 mM TCEP, 100 mM sodium ascorbate, pH 7.4, and the reaction mixture was stirring at room temperature. The progress of the reaction was monitored by analytical RP-HPLC. After completion of the reaction, the crude ligated peptide **5b** was purified by preparative RP-HPLC using Phenomenex Jupiter® C4 column (5 µm, 300 Å pore size, 30 mm I.D. × 250 mm) with a gradient of 20–80% CH_3CN (with 0.1% TFA) in 30 min with 20 mL/min flow rate. The fractions containing the desired product were pooled and lyophilized to give **5b** (21.5 mg, 68% yield). The m/z calculated for **5b** $C_{277}H_{439}N_{81}O_{88}S_8$ $[M+4H]^{4+}$: 1641.0028 Da, measured: 1641.0009 Da.

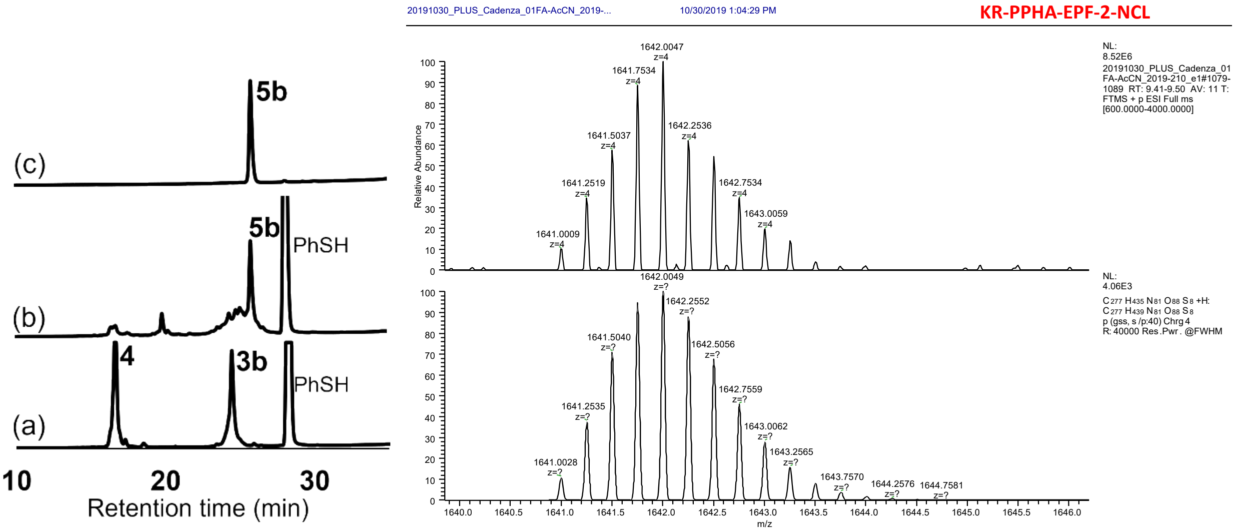

a) NCL at 0 h; b) NCL at 16 h; c) Purified linear protein **5b**

4. Chemical synthesis of cysteine protected EPF1 proteins

4.1 Synthesis of Cys(Acm) protected α-ketoacid peptide 8a

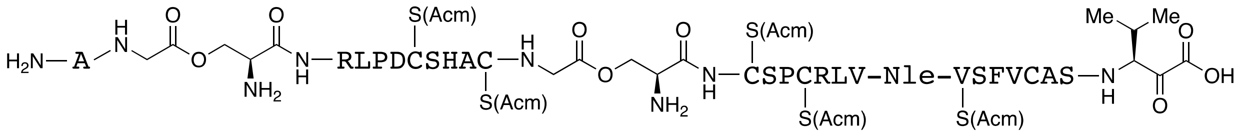

The α-ketoacid peptide **8a** was synthesized on aminomethyl polystyrene resin preloaded with Fmoc-protected-Val-α-ketoacid (0.25 mol/g loading) by automated Fmoc SPPS through peptide synthesizer. Gly-Ser isoacyl dipeptide and amino acid light after the Gly-Ser isoacyl dipeptide (Ala1 and Cys12) were introduced manually in-between the peptide sequence using HOBt (0.3 mmol, 1.0 equiv) and DIC (0.3 mmol, 1.0 equiv) coupling reagents for 2 h at room temperature.

After completion of the SPPS, the resin was dried and placed in a glass vial and mixture of 95:2.5:2.5 TFA:DODT:H_2O (20 mL/g resin) was added, the suspension was shaken at room temperature. After 2 h, the crude TFA solution was separated from resin by filtration and the filtrate was concentrated under reduced pressure. The residue was triturated with cooled Et_2O (ca. 15 mL/g resin), centrifuged and the supernatant was removed by decantation (peptide precipitating out). The trituration/washing step was repeated twice. The crude peptide dissolved in aqueous CH_3CN with 0.1% TFA and purified by preparative RP-HPLC using a Shiseido Capcell pak C18 UG80 (50 mm I.D. × 250 mm) at room temperature with a gradient of 20–80% CH_3CN (with 0.1% TFA) in 30 min, flow rate 40 mL/min and re-purified using a Phenomenex Jupiter® C18 column (5 µm, 300 Å pore size, 30 mm I.D × 250 mm) with a gradient of 20–70%

CH_3CN (with 0.1% TFA) in 30 min with 20 mL/min flow rate. The fractions containing the desired product were pooled and lyophilized to give 102 mg of peptide **8a** (obtained from 1 g of dried resin after SPPS). The m/z calculated for **8a** $C_{141}H_{234}N_{43}O_{45}S_5$ $[M+3H]^{3+}$: 1136.5310 Da, measured: 1136.5355 Da.

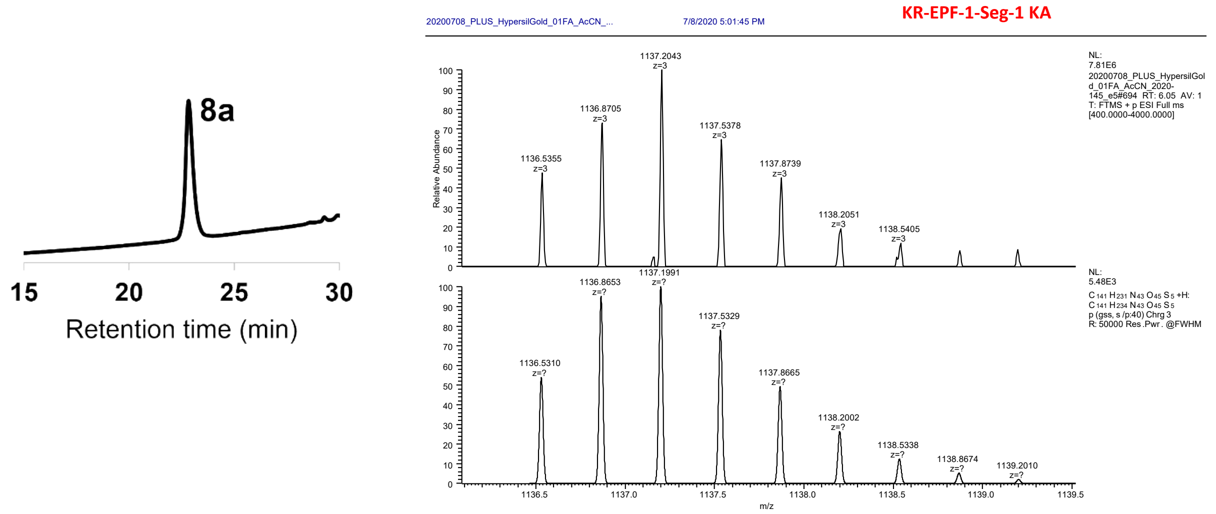

4.2 Synthesis of Cys(Acm) protected α-ketoacid peptide 8b

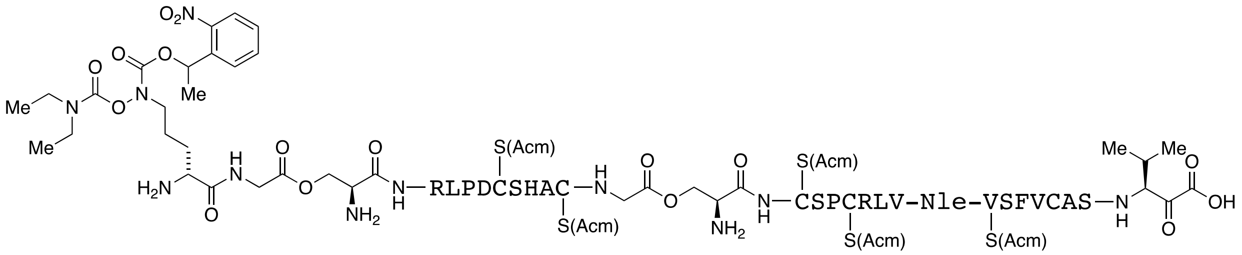

The α-ketoacid peptide **8b** was synthesized on aminomethyl polystyrene resin preloaded with Fmoc-protected-Val-α-ketoacid (0.25 mol/g loading) by automated Fmoc SPPS through peptide synthesizer. Gly-Ser isoacyl dipeptide and amino acid light after the Gly-Ser isoacyl dipeptide (Ala1 and Cys12) were introduced manually in-between the peptide sequence using HOBt (0.3 mmol, 1.0 equiv) and DIC (0.3 mmol, 1.0 equiv) coupling reagents for 2 h at room temperature.

After automated Fmoc SPPS, Fmoc-Orn HA (1.1 equiv, 0.33 mmol) was manually coupled using HATU (1.1 equiv 0.33 mmol) and NMM (2.2 equiv, 0.66 mmol) in anhydrous DMF for 2 h at room temperature. The resin was dried and placed in a glass vial and mixture of 95:2.5:2.5 TFA:DODT:H_2O (20 mL/g resin) was added, the suspension was shaken at room temperature. After 2 h, the crude TFA solution was separated from resin by filtration and the filtrate was

concentrated under reduced pressure. The residue was triturated with cooled Et_2O (ca. 15 mL/g resin), centrifuged and the supernatant was removed by decantation (peptide precipitating out). The trituration/washing step was repeated twice. The crude peptide dissolved in aqueous CH_3CN with 0.1% TFA and purified by preparative RP-HPLC using a Shiseido Capcell pak C18 UG80 (50 mm I.D. × 250 mm) at room temperature with a gradient of 20–80% CH_3CN (with 0.1% TFA) in 30 min, flow rate 40 mL/min and re-purified using a Phenomenex Jupiter® C18 column (5 µm, 300 Å pore size, 30 mm I.D × 250 mm) with a gradient of 20–70% CH_3CN (with 0.1% TFA) in 30 min with 20 mL/min flow rate. The fractions containing the desired product were pooled and lyophilized to give 180 mg of peptide **8b** (obtained from 1 g of dried resin after SPPS). The m/z calculated for **8b** $C_{157}H_{257}N_{46}O_{51}S_5$ $[M+5H]^{5+}$: 752.5501 Da, measured: 752.5533 Da.

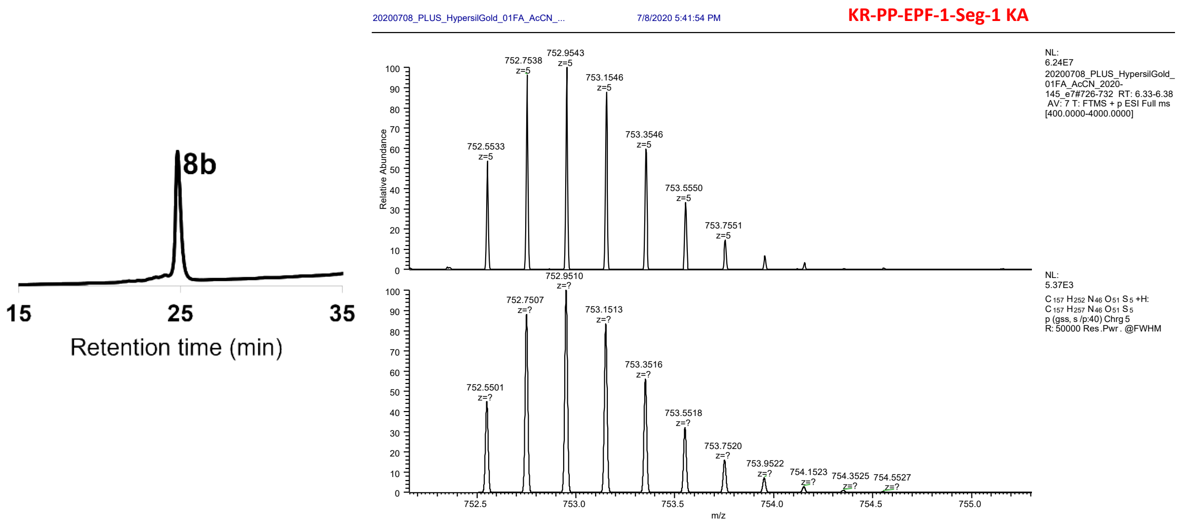

4.3 Synthesis of Cys(Acm) protected 5-oxaproline peptide 9

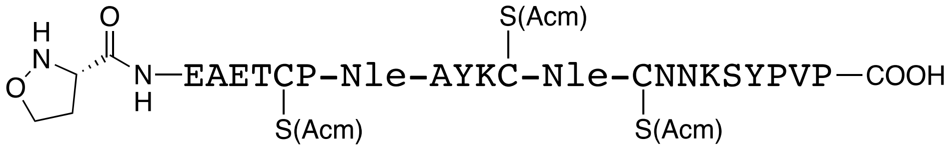

5-Oxaproline peptide **9** was prepared on HMPB-ChemMatrix resin preloaded with Fmoc-Pro-OH (0.30 mmol/g loading). After automated Fmoc SPPS, Boc-Opr (1.5 equiv) was coupled at N-terminus using HATU (1.4 equiv) and NMM (3.0 equiv) in dry DMF for 4 h at room temperature to complete the SPPS. The resin was washed several times with DMF followed by CH_2Cl_2, dried and subjected to cleavage using a mixture of 95:2.5:2.5 TFA:TIPS:H_2O (20 mL/g resin) for 2 h at room temperature. The crude TFA solution was separated from the resin by filtration and the filtrate was concentrated under reduced pressure. The residue was triturated with cooled Et_2O (ca.

25 mL/g resin), centrifuged and the supernatant was removed by decantation. This trituration/washing step was repeated twice (peptide precipitating out). The crude peptide was dissolved in aqueous CH_3CN with 0.1% TFA and purified by RP-HPLC using a Shiseido Capcell pak C18 UG80 (50 mm I.D. × 250 mm) at room temperature with a gradient of 15–55% CH_3CN (with 0.1% TFA) in 20 min, flow rate 40 mL/min and re-purified by a Phenomenex Jupiter® C18 column (5 µm, 300 Å pore size, 30 mm I.D × 250 mm) with a gradient of 20–70% CH_3CN (with 0.1% TFA) in 30 min with 20 mL/min flow rate. The pure product fractions were cooled and lyophilized to obtain 160 mg of desired 5-oxaproline peptide **9** (obtained from 1 g of dried resin after SPPS). The purity and identity of peptide **9** was confirmed using analytical HPLC and ESI-HRMS. The m/z calculated for peptide **9** $C_{115}H_{182}N_{29}O_{37}S_3$ $[M+3H]^{3+}$: 885.7466 Da, measured: 885.7502 Da.

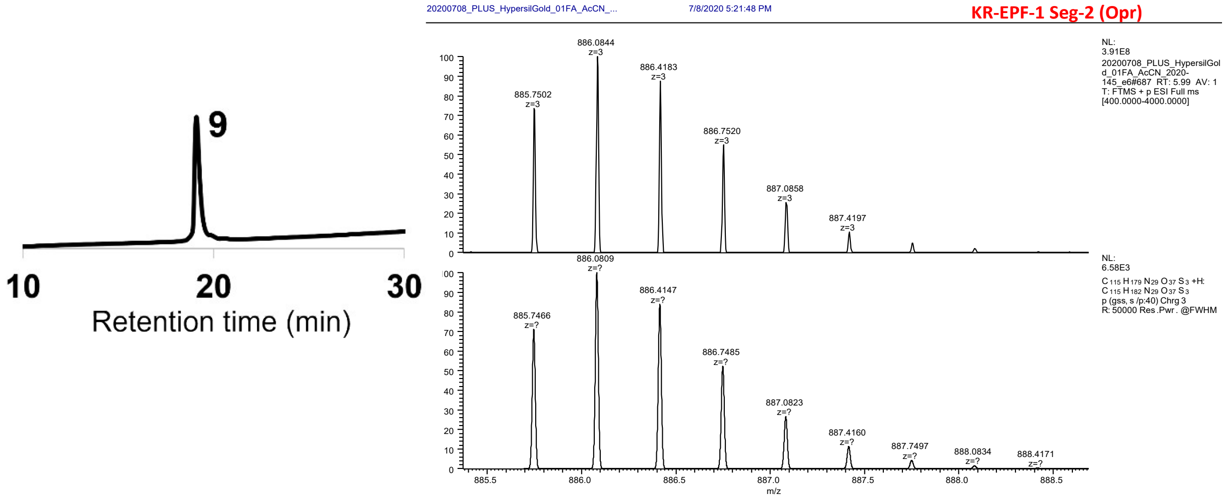

4.4 Synthesis of Cys(Acm) protected EPF1 protein 11a by KAHA ligation

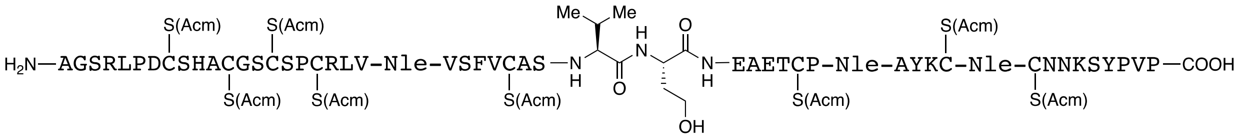

5-Oxaproline peptide **9** (23.4 mg, 8.8 µmol, 1.5 equiv) and α-ketoacid peptide **8a** (20 mg, 5.9 µmol, 1.0 equiv) were weighed into a glass vial and dissolved in a mixture of 9:1 DMSO/H_2O (20 mM peptide concentration of **8a**) with 0.1 M oxalic acid. The mixture was heated to 60 °C for 30 h. After 30 h, the reaction was subjected to *O* to *N* acyl shift by dilution to 10-fold volume with 6.0 M Gdn•HCl solution set to pH 9.6 and mixture was stirred at room temperature for 4 h. The reaction mixture was purified by preparative RP-HPLC using Phenomenex Jupiter® C18 column (5 µm, 300 Å pore size, 30 mm I.D × 250 mm) with a gradient of 20–70% CH_3CN (with

0.1% TFA) in 30 min with 20 mL/min flow rate. The fractions containing the desired product were pooled and lyophilized to give pure **11a (**26 mg, 54% yield). m/z calculated for **11a** $C_{255}H_{416}N_{72}O_{80}S_8$ $[M+6H]^{6+}$: 1003.8072 Da, measured: 1003.8108 Da.

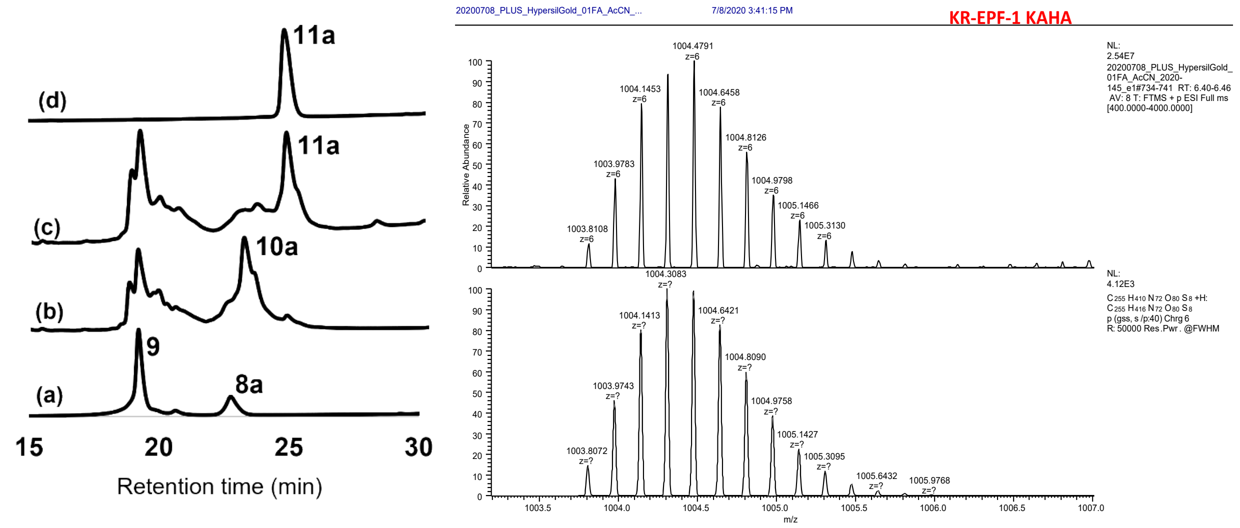

a) KAHA Ligation at 0 h; b) KAHA Ligation at 30 h; c) *O* to *N* acyl shift at 4 h; d) Purified linear protein **11a**

4.5 Synthesis of Cys(Acm) protected EPF1 protein 11b by KAHA ligation

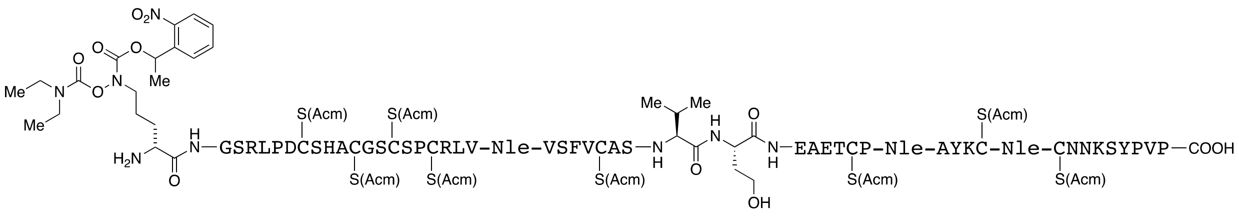

5-Oxaproline peptide **9** (23.4 mg, 8.8 μmol, 1.5 equiv) and α-ketoacid peptide **8b** (20 mg, 5.3 μmol, 1.0 equiv) were weighed into a glass vial and dissolved in a mixture of 9:1 DMSO/H_2O (20 mM peptide concentration of **8b**) with 0.1 M oxalic acid. The mixture was heated to 60 °C for 30 h. After 30 h, the reaction was subjected to *O* to *N* acyl shift by dilution to 10-fold volume with 6.0 M Gdn•HCl solution set to pH 9.6 and mixture was stirred at room temperature for 4 h. The reaction mixture was purified by preparative RP-HPLC using Phenomenex Jupiter® C18 column (5 μm, 300 Å pore size, 30 mm I.D × 250 mm) with a gradient of 20–70% CH_3CN (with 0.1% TFA) in 30 min with 20 mL/min flow rate. The fractions containing the desired product were pooled and lyophilized to give pure **11b** (21 mg, 62% yield). m/z calculated for **11b** $C_{271}H_{437}N_{75}O_{86}S_8$ $[M+6H]^{6+}$: 1062.3310 Da, measured: 1062.3295 Da.

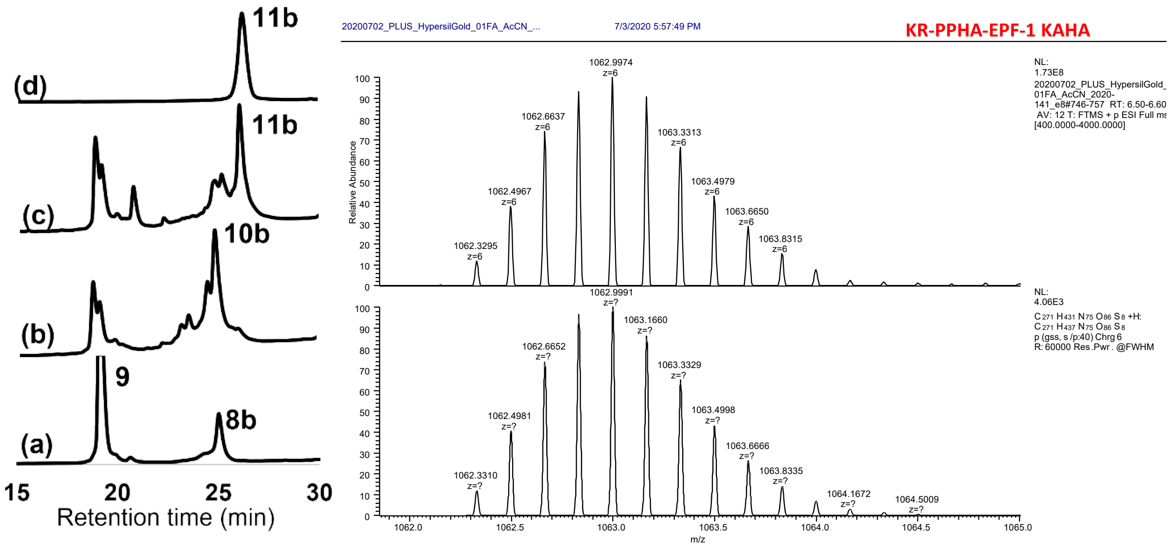

a) KAHA Ligation at 0 h; b) KAHA Ligation at 30 h; c) *O* to *N* acyl shift at 4 h; d) Purified linear protein **11b**

5. Deprotection of Acm group and protein folding

5.1 General procedure of Acm deprotection

Cysteine Acm protected proteins were dissolved in 50% aq. acetic acid (v/v) containing 1% (w/v) AgOAc (1 mM of the linear protein concentration) and the mixture was stirred at 45 °C for 2 h. The mixture was quenched with 10% DTT in 50% aq. acetic acid (w/v/v), and the precipitation was separated by centrifugation. The precipitate was washed with 50% aq. acetic acid solution (v/v) and the combined supernatant was purified by preparative RP-HPLC.

5.2 Synthesis of reduced EPF2 protein 6a

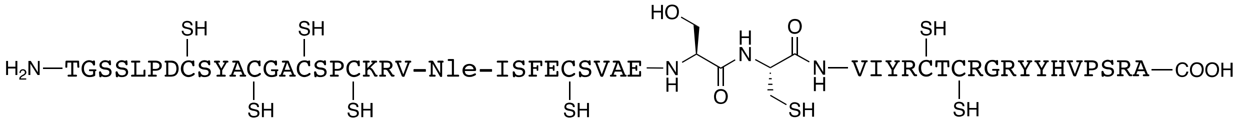

The reduced peptide **6a** was synthesized according to the general procedure **5.1** using Cys(Acm) Protected linear protein **5a** (10 mg, 1.63 μM, 1.0 equiv). at 45 °C. The obtained solution was purified by preparative HPLC using a Phenomenex Jupiter® C18 column (5 μm, 300 Å pore size, 21.2 mm I.D. × 250 mm) preheated to 60 ºC, with a gradient of 30–80% CH_3CN with 0.1% TFA in 30 min, flow rate 10 mL/min. The fractions containing the desired peptide were pooled and lyophilized to give pure **6a** (6.5 mg, 70% yield). The m/z calculated for **6a** $C_{237}H_{379}N_{70}O_{74}S_8[M+5H]^{5+}$: 1129. 1157 Da; measured: 1129.1156 Da.

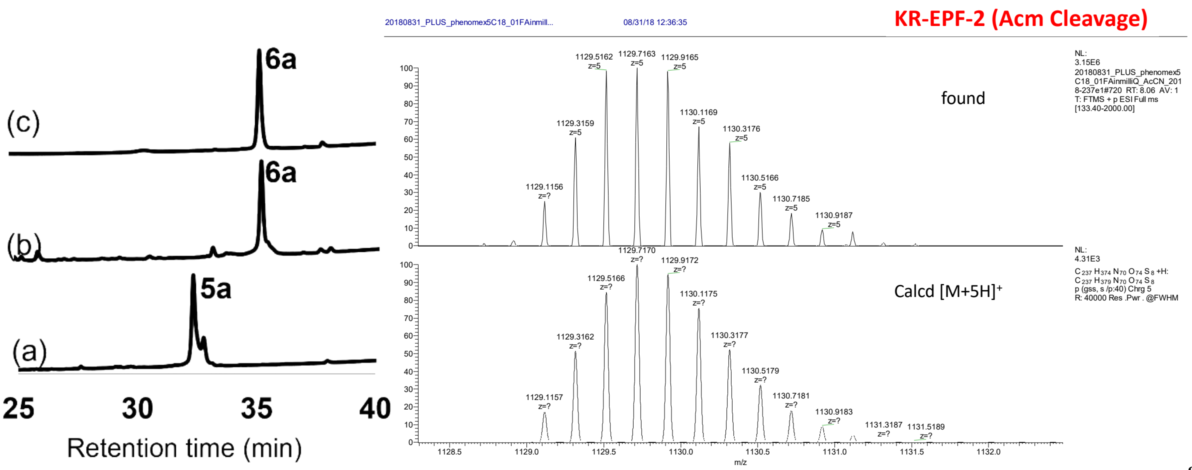

a) Acm deprotection at 0 h; b) Acm deprotection at 2 h; c) Purified reduced linear protein **6a**

5.3 Synthesis of reduced EPF2 protein 6b

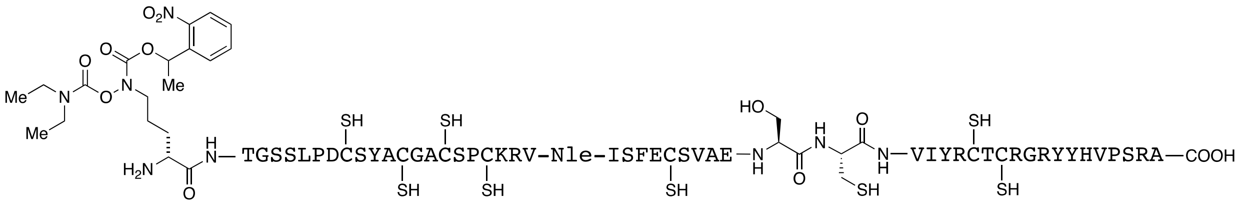

The reduced peptide **6b** was synthesized according to the general procedure **5.1** using Cys(Acm) Protected linear protein **5b** (10 mg, 1.0 equiv) at 45 °C. The obtained solution was purified by preparative HPLC using a Phenomenex Jupiter® C18 column (5 µm, 300 Å pore size, 21.2 mm I.D. × 250 mm) preheated to 60 °C, with a gradient of 30–80% CH_3CN with 0.1% TFA in 30 min, flow rate 10 mL/min. The fractions containing the desired peptide were pooled and lyophilized to give pure **6b** (6.6 mg, 72% yield). The m/z calculated for **6b** $C_{256}H_{404}N_{74}O_{81}S_8[M+4H]^{4+}$: 1516.6878 Da; measured: 1516.6868 Da.

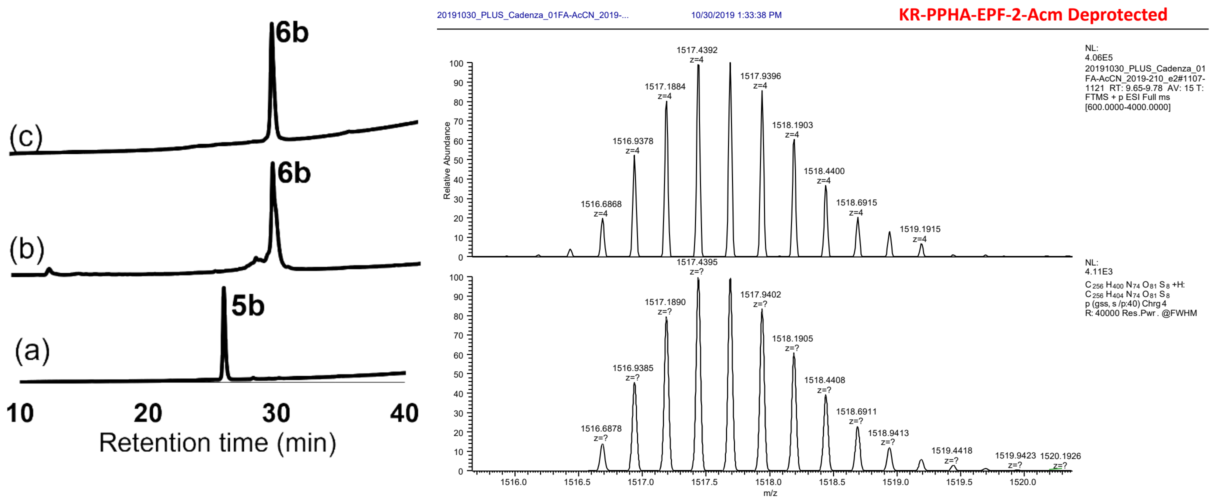

a) Acm deprotection at 0 h; b) Acm deprotection at 2 h; c) Purified product **6b**

5.4 Synthesis of reduced EPF1 protein 12a

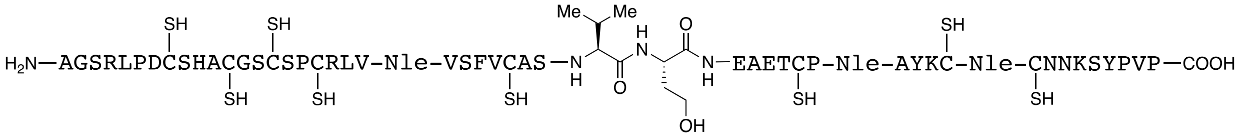

The reduced protein **12a** was synthesized according to the general procedure **5.1** using Cys(Acm) protected linear peptide **11a** (10 mg, 1.6 µmol, 1.0 equiv). The obtained mixture was purified by preparative HPLC using a Phenomenex Jupiter® C18 column (5 µm, 300 Å pore size, 21.2 mm I.D. × 250 mm) with a gradient of 30–80% CH_3CN with 0.1% TFA in 30 min, flow rate 10 mL/min. The fractions containing the desired peptide were pooled and lyophilized to give pure peptide **12a** (5.9 mg, 65% yield). The m/z calculated for **12a** $C_{231}H_{376}N_{64}O_{72}S_8[M+6H]^{6+}$: 909.0910 Da; measured: 909.0894 Da.

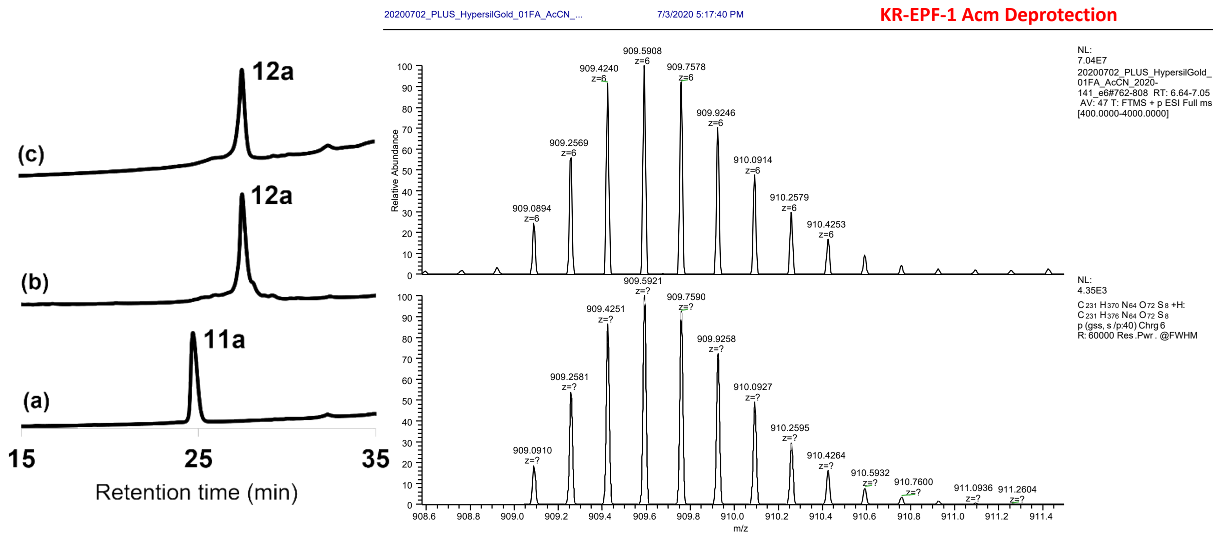

a) Acm deprotection at 0 h; b) Acm deprotection at 2 h; c) Purified reduced linear protein **5a**

5.5 Synthesis of reduced EPF1 protein 12b

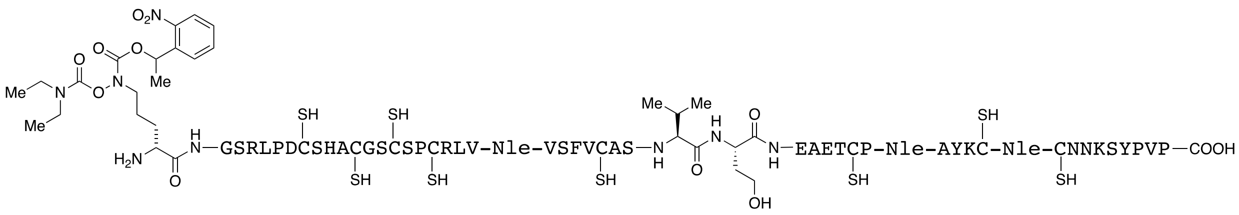

The reduced protein **12b** was synthesized according to the general procedure **5.1** using Cys(Acm) protected linear peptide **11b** (10 mg, 1.72 μmol, 1.0 equiv). The obtained mixture was purified by preparative HPLC using a Phenomenex Jupiter® C18 column (5 μm, 300 Å pore size, 21.2 mm I.D. × 250 mm) with a gradient of 30–80% CH_3CN with 0.1% TFA in 30 min, flow rate 10 mL/min. The fractions containing the desired peptide were pooled and lyophilized to give pure peptide **12b** (6.6 mg, 70% yield). The m/z calculated for **12b** $C_{247}H_{397}N_{67}O_{78}S_8[M+6H]^{6+}$: 967.6149 Da; measured: 967.6135 Da.

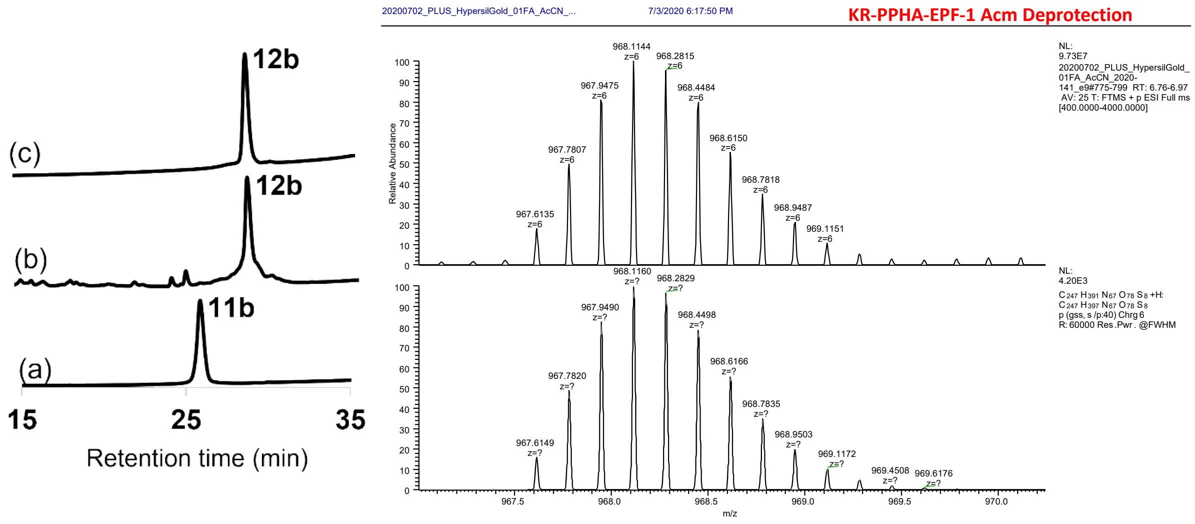

a) Acm deprotection at 0 h; b) Acm deprotection at 2 h; c) Purified reduced linear protein **12b**

5.6 General procedure of protein folding

The Acm-deprotected, reduced linear protein was denatured using denatured buffer containing 6 M Gdn•HCl + 0.1 M Tris•HCl buffer, pH 6.8 (0.5 mM peptide concentration) stirred at room temperature for 1 h open to air. After 1 h, the mixture was diluted with 8-fold volume of folding buffer containing 5.0 mM of reduced glutathione and 2.5 mM oxidized glutathione. The reaction was incubated in a shaker with slow movement at 4 °C for 30 h. The folding progress was

monitored by analytical RP-HPLC. The resulting solution was acidified with aqueous HCl adjust to pH 4–5 and purified by preparative HPLC.

5.7 Synthesis of EPFL9 protein 2a

The folded EPFL9 protein **2a** was synthesized according to general procedure **5.6** using reduced

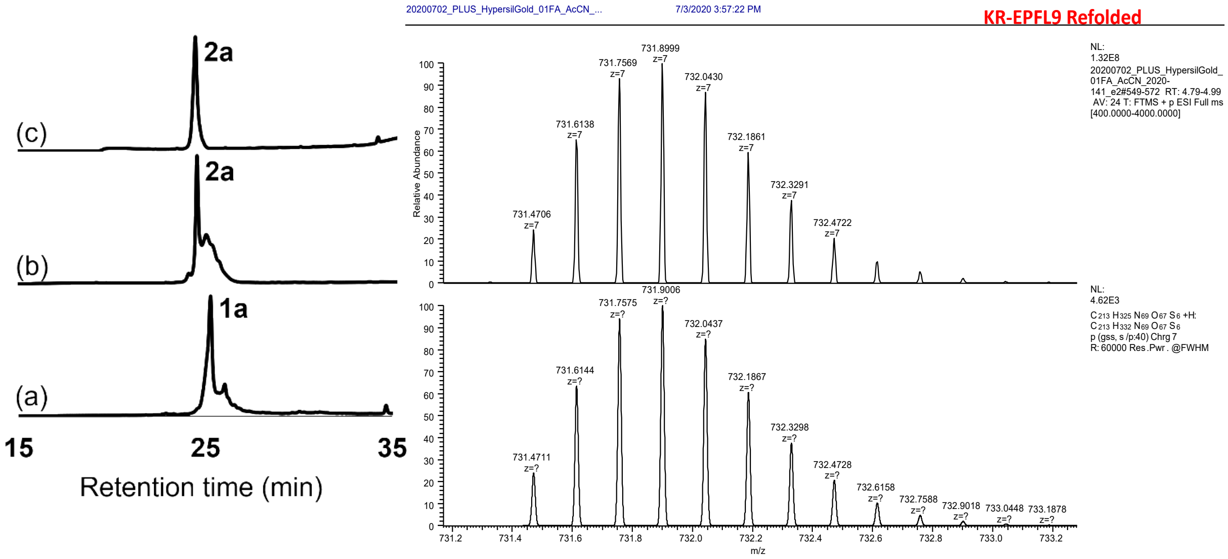

linear protein **1a** (10 mg, 1.9 µM). The folding buffer pH 8.0 was used. The resulting reaction mixture was purified by preparative HPLC using a Phenomenex Jupiter® C18 column (5 µm, 300 Å pore size, 21.2 mm I.D. × 250 mm) with the gradient 10–60% CH_3CN with 0.1% TFA in 30 min, flow rate 10 mL/min. The fractions containing desired product were pooled and lyophilized to give pure folded EPFL9 **2a** (5.8 mg, 58% yield). The m/z calculated for **2a** $C_{213}H_{332}N_{69}O_{67}S_6$ $[M+7H]^{7+}$: 731.4711 Da, measured: 731.4706 Da.

a) Folding at 0 h; b) Folding at 30 h; c) Purified Folded EPFL9 Protein **2a**

5.8 Synthesis of EPFL9 protein 2b

The folded EPFL9 protein **2b** was synthesized according to general procedure **5.6** using reduced linear protein **1b** (10 mg, 1.8 µM). The folding buffer pH 8.0 was used. The resulting reaction mixture was purified by preparative HPLC using a Phenomenex Jupiter® C18 column (5 µm, 300 Å pore size, 21.2 mm I.D. × 250 mm) with the gradient 10–60% CH_3CN with 0.1% TFA in 30 min, flow rate 10 mL/min. The fractions containing desired product were pooled and lyophilized to give pure folded EPFL9, **2b** (4.7 mg, 47% yield). The m/z calculated for **2b** $C_{232}H_{358}N_{73}O_{74}S_6$ $[M+7H]^{7+}$: 791.7826 Da, measured 791.7817 Da.

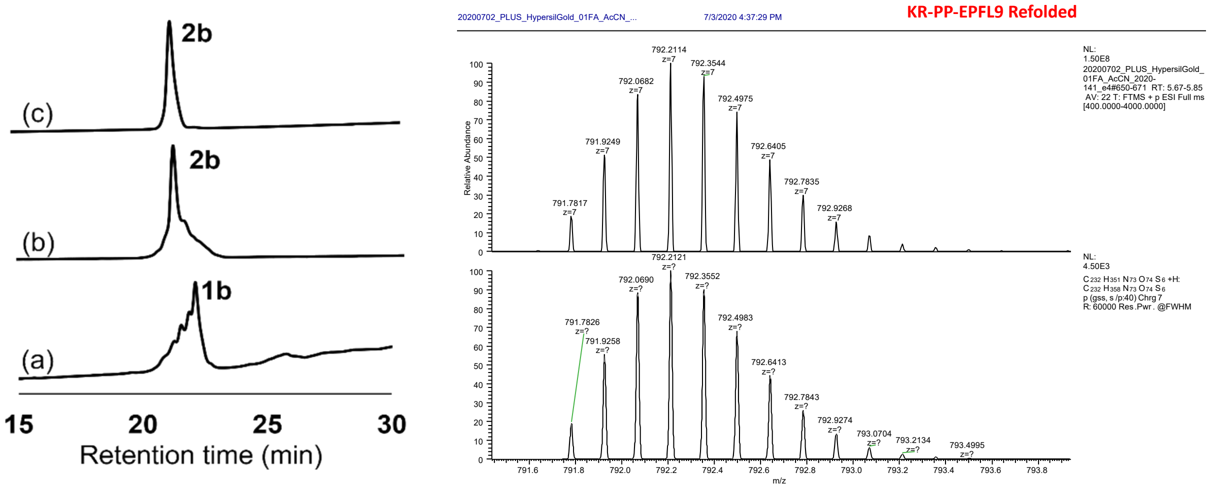

a) Folding at 0 h; b) Folding at 30 h; c) Purified protein **2b**

5.9 Synthesis of EPF2 protein 7a

Folded EPF2 protein **7a** was synthesized according to the general procedure **5.6** using reduced protein **6a** (2 mg, 0.35 µM). The folding buffer pH 7.0 was used. The resulting solution was purified by preparative RP-HPLC using a Phenomenex Jupiter® C18 column (5 µm, 300 Å pore size, 21.2 mm I.D. × 250 mm) CH_3CN with 0.1% TFA in 30 min, flow rate 10 mL/min. The fractions containing desired product were pooled and lyophilized to give pure folded EPF2 **7a** (1.3 mg, 55% yield). The m/z calculated for folded EPF2 protein **7a** $C_{237}H_{371}N_{70}O_{74}S_8$ $[M+5H]^{5+}$: 1127.5032 Da; measured: 1127.5030 Da.

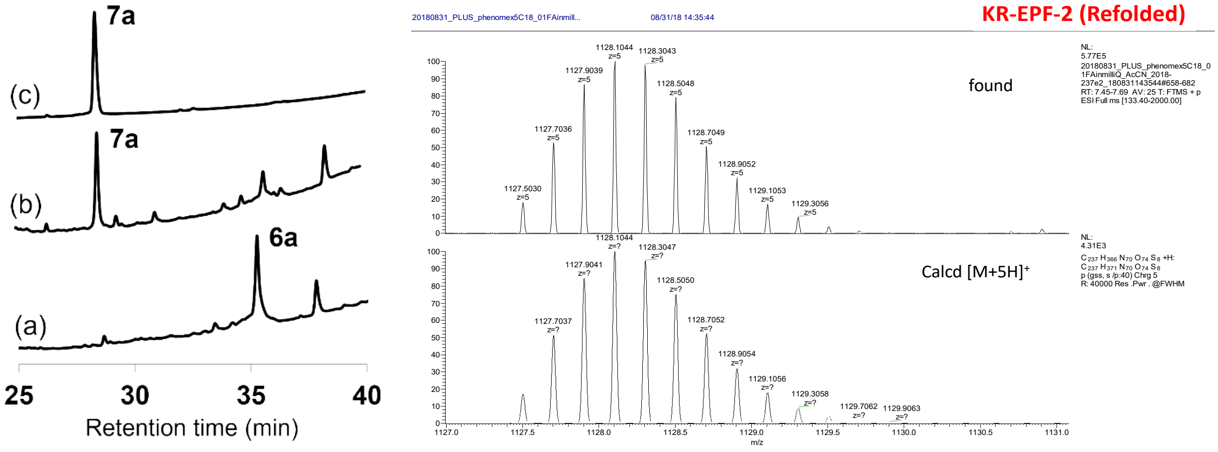

a) Folding at 0 h; b) Folding at 30 h; c) Purified EPF2 Protein **7a**

5.10 Synthesis of EPF2 protein 7b

Folded EPF2 protein **7b** was synthesized according to the general procedure **5.6** using reduced protein **6b** (2 mg, 0.33 μm). The folding buffer pH 7.0 was used. The resulting solution was purified by preparative RP-HPLC using a Phenomenex Jupiter® C18 column (5 μm, 300 Å pore size, 21.2 mm I.D. × 250 mm) CH_3CN with 0.1% TFA in 30 min, flow rate 10 mL/min. The fractions containing desired product were pooled and lyophilized to give pure folded EPF2 **7a** (1.2 mg, 60% yield). The m/z calculated for folded EPF2 protein **7b** $C_{256}H_{396}N_{74}O_{81}S_8$ $[M+4H]^{4+}$: 1514.6722 Da; measured: 1514.6682 Da.

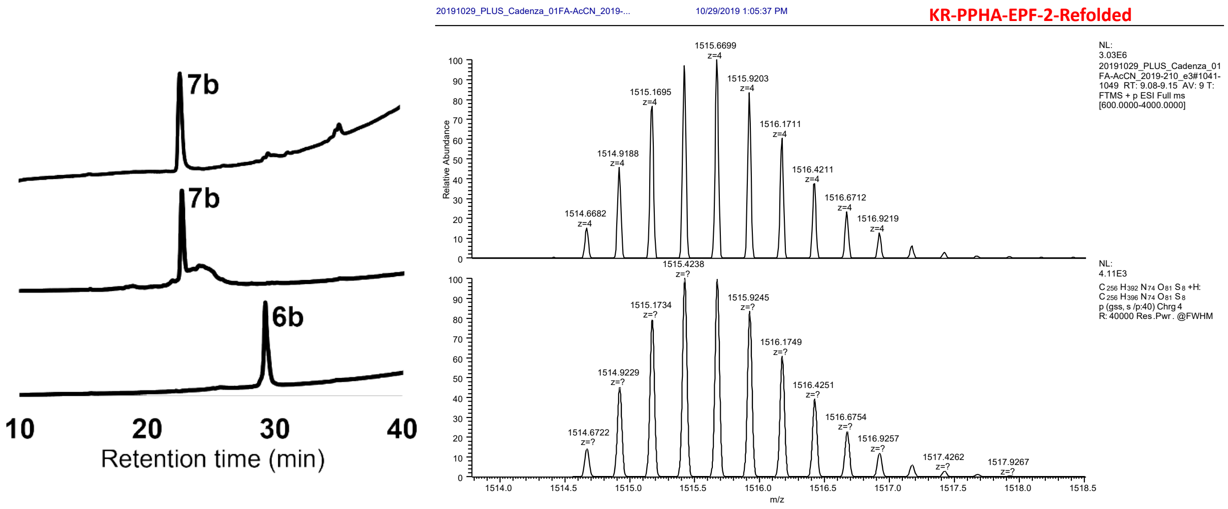

a) Folding at 0 h; b) Folding at 30 h; c) Purified EPF2 Protein **7b**

5.11 Synthesis of EPF1 protein 13a

Folded EPF1 protein **13a** was synthesized according to the general procedure **5.6** with 1 mg of reduced linear protein **12a**. The folding buffer pH 8.0 was used. The resulting mixture was purified by preparative HPLC using a Phenomenex Jupiter® C18 column (5 μm, 300 Å pore size, 21.2 mm I.D. × 250 mm) CH_3CN with 0.1% TFA in 30 min, flow rate 10 mL/min. The fractions containing the folded protein were pooled and lyophilized to give pure folded EPF1 **13a** (0.55 mg, 55% yield). The m/z calculated for folded EPF1 protein **13a** $C_{231}H_{368}N_{64}O_{72}S_8$ $[M+6H]^{6+}$: 907.7472 Da; measured: 907.7461 Da.

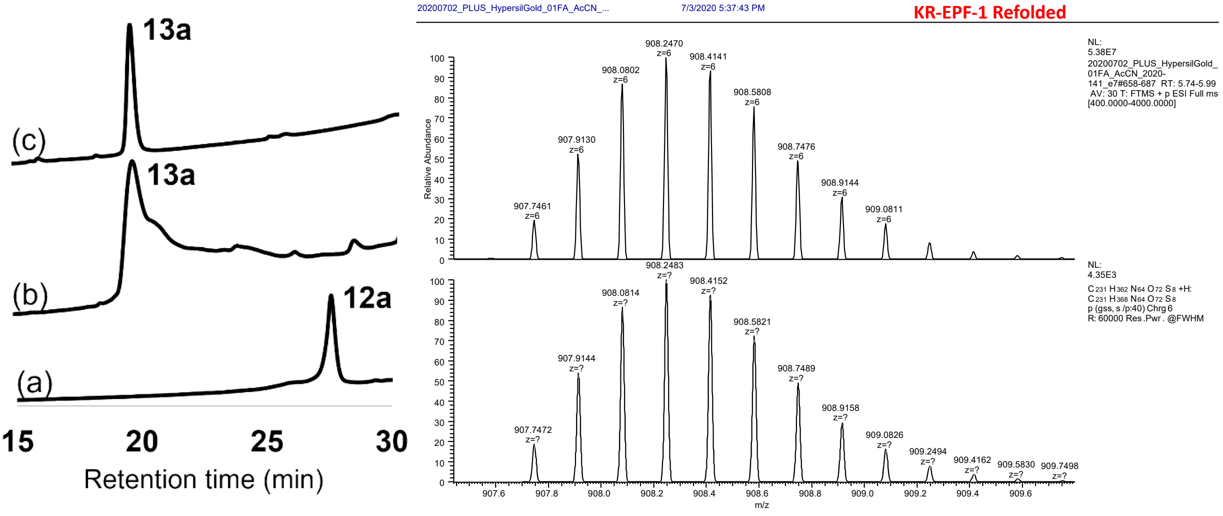

a) Folding at 0 h; b) Folding at 30 h; c) Purified folded EPF1 Protein **13a**

5.12 Synthesis of EPF1 protein 13b

Folded EPF1 protein **13b** was synthesized according to the general procedure **5.6** with 1 mg of reduced linear protein **12b**. The folding buffer pH 8.0 was used. The resulting mixture was purified by preparative HPLC using a Phenomenex Jupiter® C18 column (5 µm, 300 Å pore size, 21.2 mm I.D. × 250 mm) CH_3CN with 0.1% TFA in 30 min, flow rate 10 mL/min. The fractions containing the folded protein were pooled and lyophilized to give pure folded EPF1, **13b** (0.6 mg, 62% yield). The m/z calculated for folded EPF1 protein **13b** $C_{247}H_{389}N_{67}O_{78}S_8$ $[M+6H]^{6+}$: 966.2711 Da; measured: 966.2699 Da.

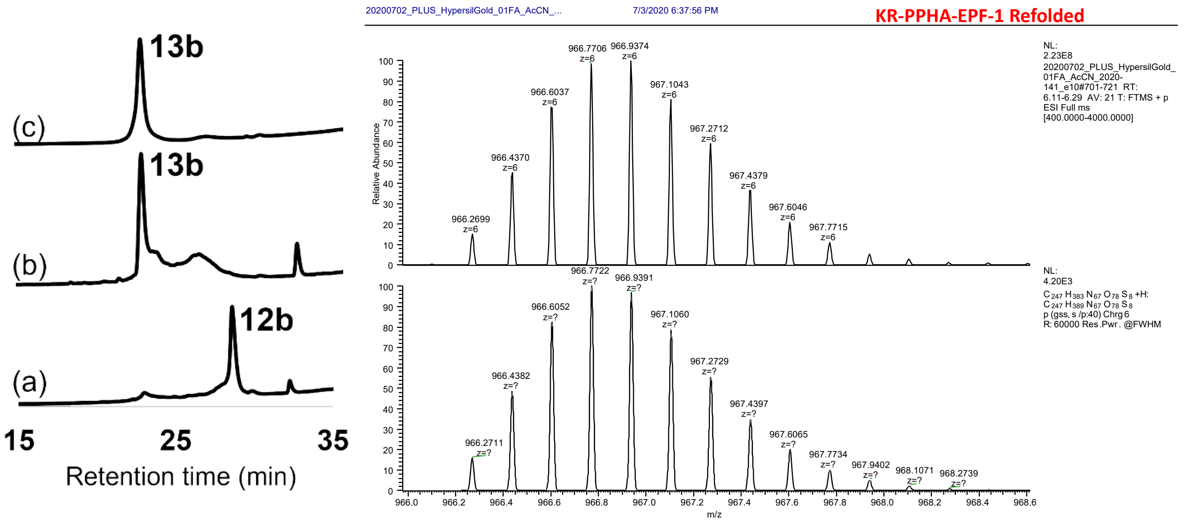

a) Folding at 0 h; b) Folding at 30 h; c) Purified folded PPHA-EPF1 protein **13b**

6. Synthesis of potassium acyltrifluoroborates (KATs) compounds

6.1 Synthesis of Azido-KAT 15

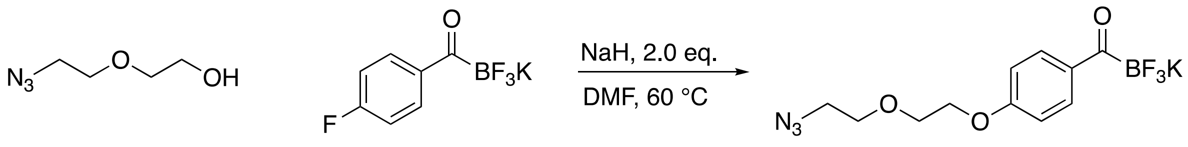

15

The sodium hydride (60% disperse in mineral oil, 9.6 mmol, 2.0 equiv) was added to the solution of 2-(2-azidoethoxy)ethan-1-ol (4.8 mmol, 1.1 equiv) in anhydrous DMF (15.0 mL) stirred at room temperature for 30 minutes. The 4-fluoro Phenyl KAT (4.34 mmol, 1.0 equiv) was added to the reaction mixture and stirred at 60 °C. After 16 h, saturated aqueous KF (0.5 mL) was added dropwise to the reaction mixture and stirred for 30 min at room temperature. The reaction mixture was filtered and washed with CH_2Cl_2 (3 × 50 mL) and acetone (3 × 50 mL). The remaining pale-yellow precipitate (containing **15** and inorganic salts) was washed multiple times with DMF (typically 200 – 250 mL) until the precipitate become colorless. The filtrate containing CH_2Cl_2, acetone and DMF was concentrated under reduced pressure to yield the product **15** as a yellow solid. (1.35 g, 91 %yield). ^{1}H NMR (400 MHz, acetone-*d6*): δ = 8.07 (d, J = 8.4 Hz, 2H), 6.93 (d, J = 9.2 Hz, 2H), 4.22 (t, J = 4.8 Hz, 2H), 3.88 (t, J = 5.2 Hz, 2H), 3.76 (t, J = 5.2 Hz, 2H), 3.42 (t, J = 4.8 Hz, 2H). ^{13}C NMR (100 MHz, acetone-*d6*): δ = 161.4, 130.6, 113.4, 70.0, 69.3, 67.5, 50.5. ^{19}F NMR (376 MHz, acetone-*d6*): δ = –144.5. HRMS (ESI neg.): Exact mass calcd for $C_{11}H_{12}BF_3N_3O_3$ $[M–K]^-$: 302.0929, found: 302.0926.

^{1}H NMR (400 MHz, acetone-*d6*)

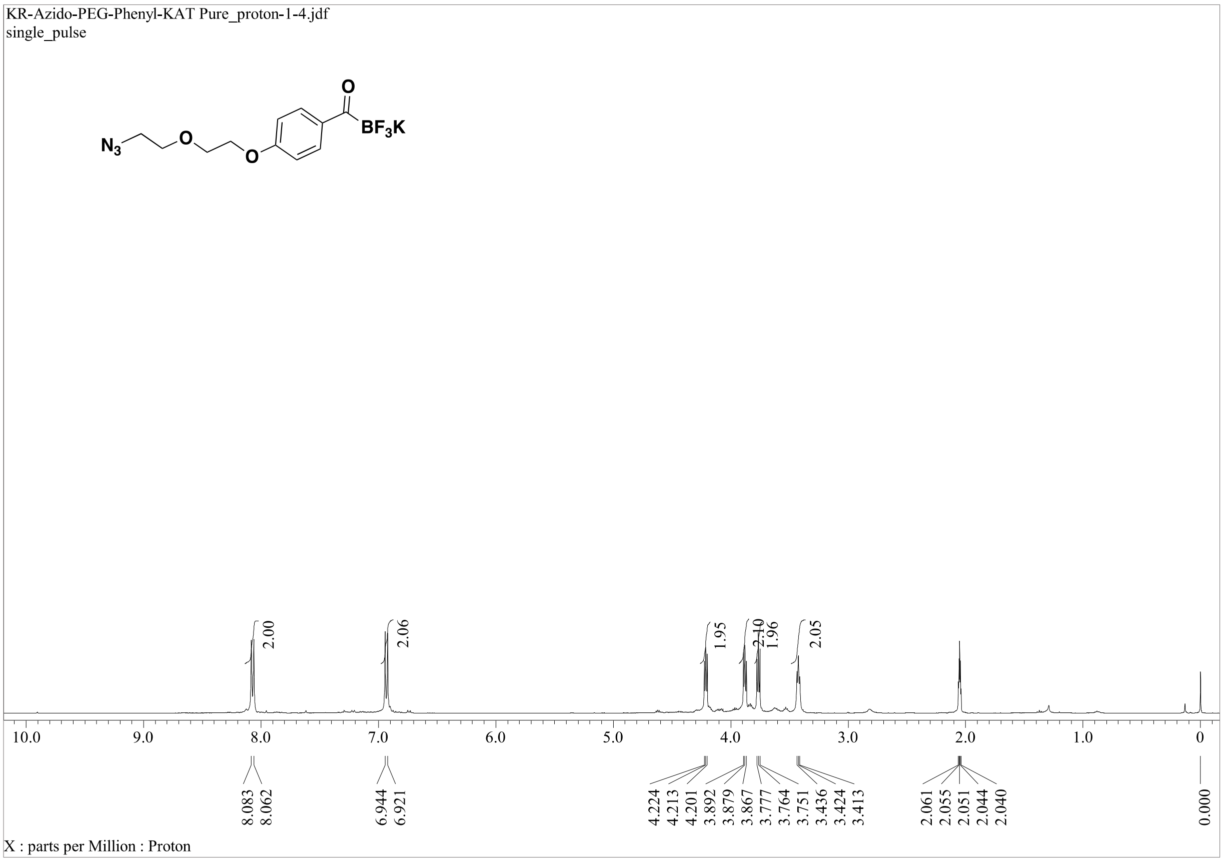

^{13}C NMR (100 MHz, acetone-*d6*)

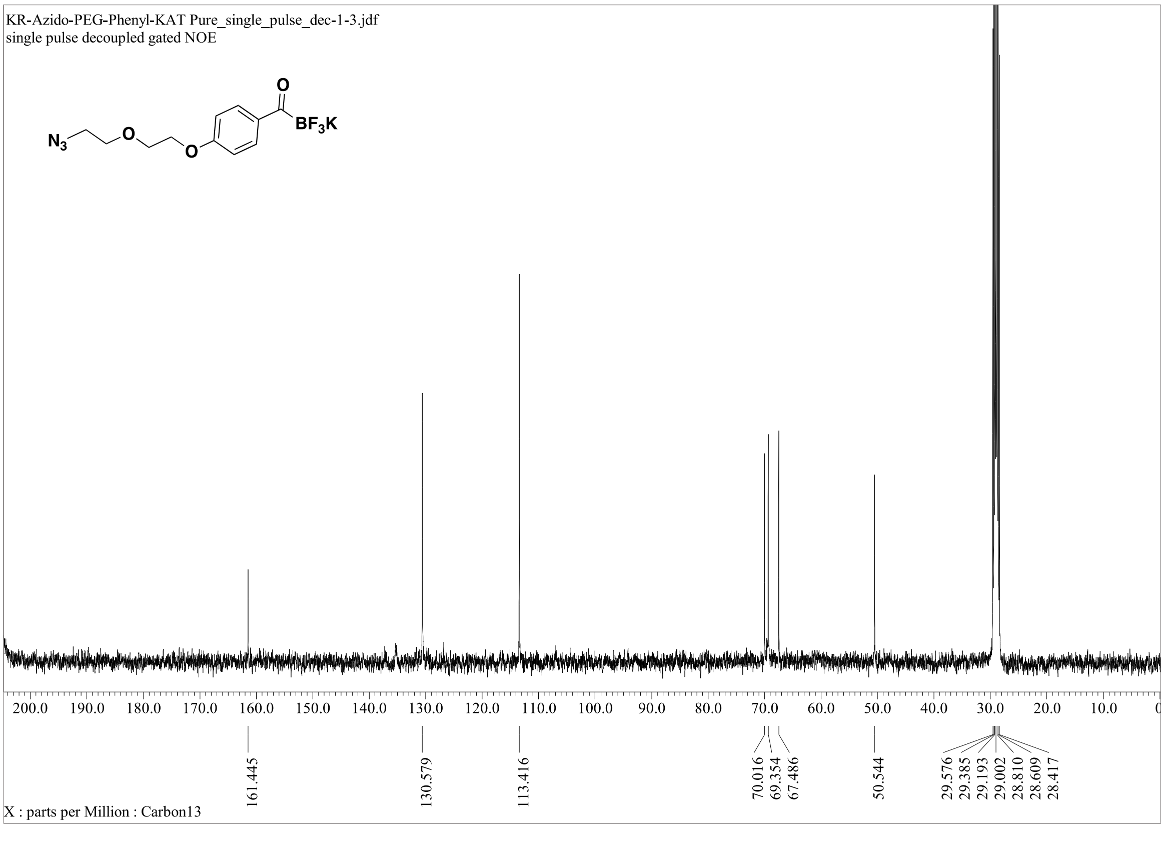

^{19}F NMR (376 MHz, acetone-*d6*)

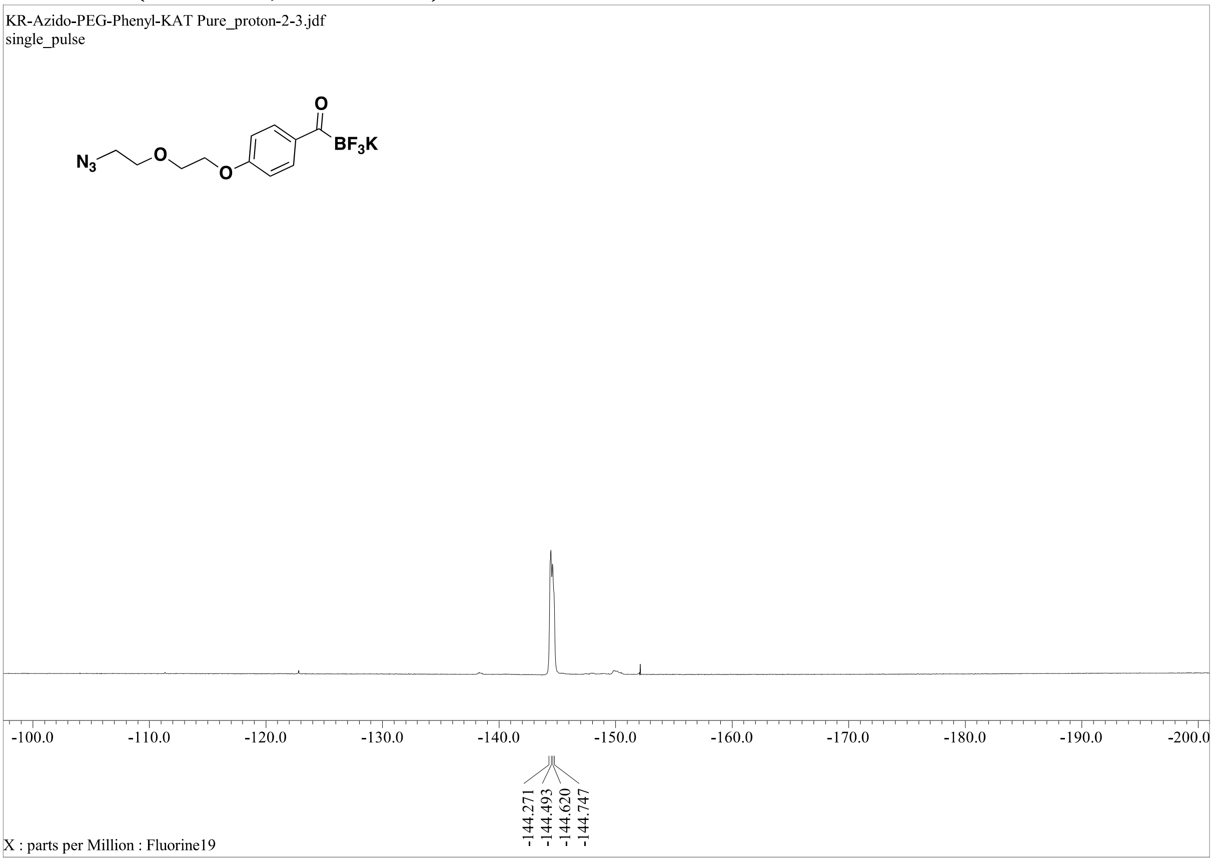

ESI-HRMS

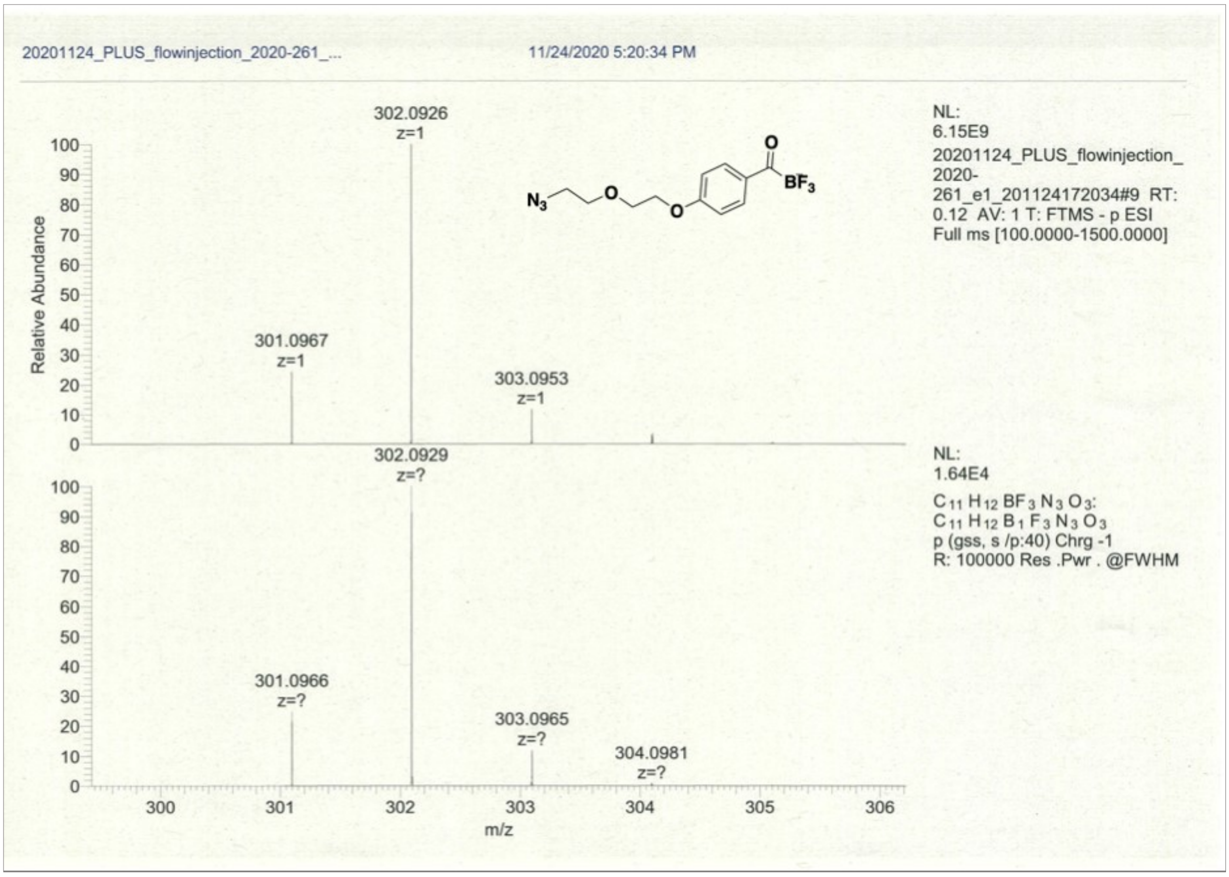

6.2 General procedure of Dye-KAT synthesis by Huisgen cycloaddition

Alkyne functionalized dyes **14a–e** (1.0 equiv), azido ethoxy ethyl KAT **15** (1.0 equiv), copper iodide (1.0 equiv) was dissolved in 50% aqueous CH_3CN and triethylamine (3.0 equiv) was added, stirred at 65 °C. After 16 h, the reaction mixture cooled to room temperature and aqueous KF solution (0.5 mL) was added, stirred another 15 min at room temperature. The crude mixture was diluted with brine solution and extracted with CH_2Cl_2 or ethyl acetate. The organic extracts were collected, dried with Na_2SO_4, and evaporated in vacuo. The crude residue was purified by column chromatography on silica gel (eluting with acetone/CH_2Cl_2 or CH_3CN/water) to give **16a–e** as solid.

6.3 Synthesis of dansyl-KAT 16a

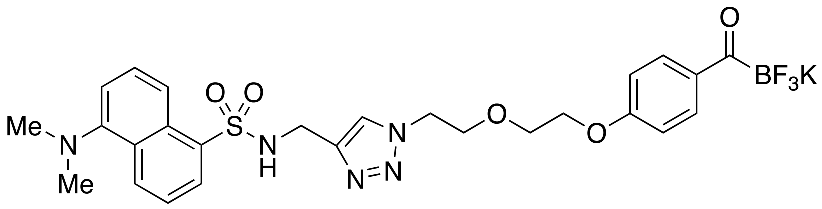

The product **16a** was synthesized according to the general procedure **6.2** using 5-(dimethylamino)-*N*-(prop-2-yn-1-yl)naphthalene-1-sulfonamide[6,7] **14a** (100 mg, 0.35 mmol, 1.0 equiv) and (4-(2-(2-azidoethoxy)ethoxy)phenyl)(trifluoro-λ4-boraneyl)methanone, potassium salt **15** (118 mg, 0.35 mmol, 1.0 equiv), CuI (66 mg, 0.35 mmol, 1.0 equiv) and Et_3N (97.5 μL, 0.7 mmol, 2.0 equiv) dissolved in 3.5 mL of 50% aqueous CH_3CN. White solid, isolated yield 120 mg (0.19 mmol, 55%). ^{1}H NMR (400 MHz, acetone-*d6*): δ = 8.52 (d, *J* = 8.8 Hz, 1H), 8.36 (d, *J* = 8.8 Hz, 1H), 8.19 (dd, *J* = 7.6, 1.2 Hz, 1H), 8.06 (d, *J* = 9.2 Hz, 2H), 7.57–7.46 (m, 3H), 7.24 (d, *J* = 1.5 Hz, 1H), 6.91 (d, *J* = 9.2 Hz, 2H), 4.37 (t, *J* = 5.6 Hz, 2H), 4.18–4.12 (m, 4H), 3.81–3.75 (m, 4H), 2.85 (s, 6H). ^{13}C NMR (100 MHz, acetone-*d6*): δ = 161.8, 151.7, 143.7, 136.3, 131.7, 130.9, 129.8, 129.8, 129.7, 129.1, 127.9, 123.4, 123.1, 119.7, 115.3, 114.3, 113.6, 69.3, 69.2, 67.3, 49.6, 44.8, 38.5. ^{19}F NMR (376 MHz, acetone-*d6*): δ = 145.2. HRMS (ESI neg.): Exact mass calcd for $C_{26}H_{28}BF_3N_5O_5S$ $[M–K]^-$: 509.1867, found 590.1863.

^{1}H NMR (400 MHz, acetone-*d6*)

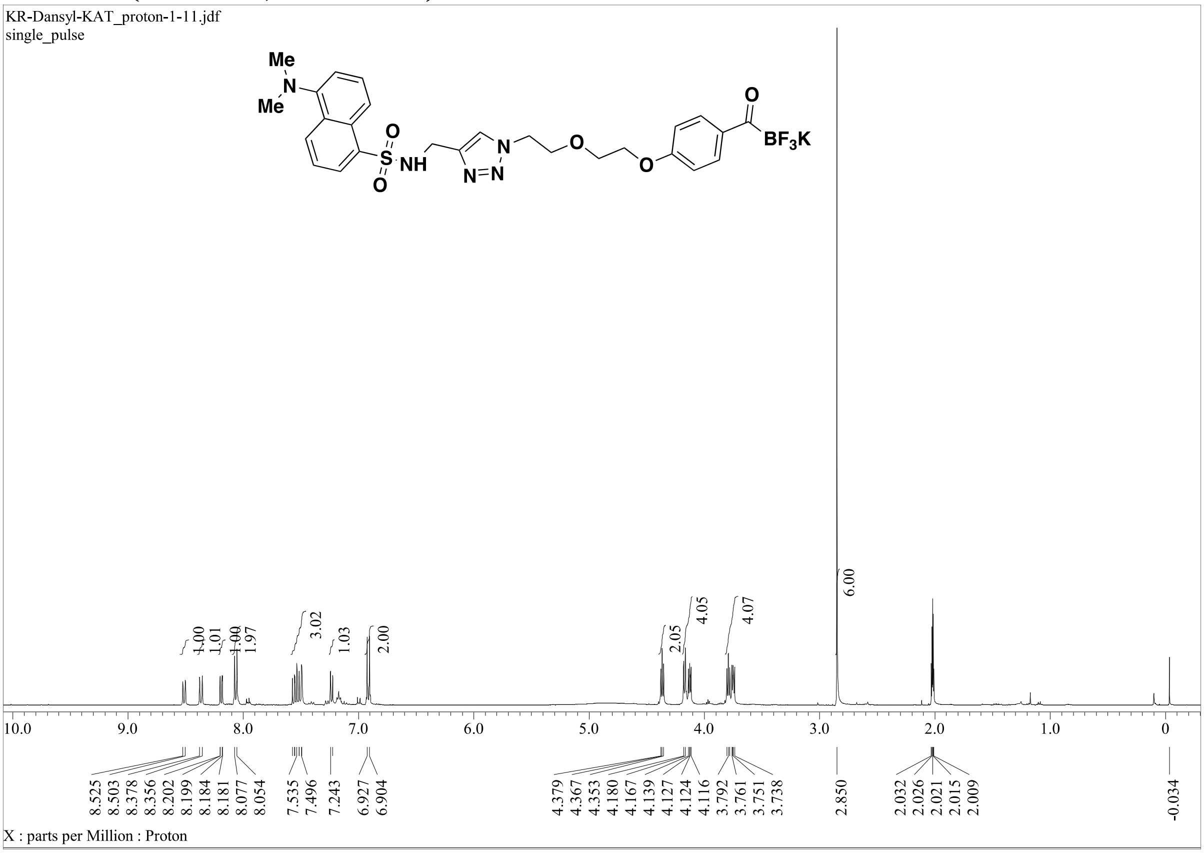

^{13}C NMR (100 MHz, acetone-*d6*)

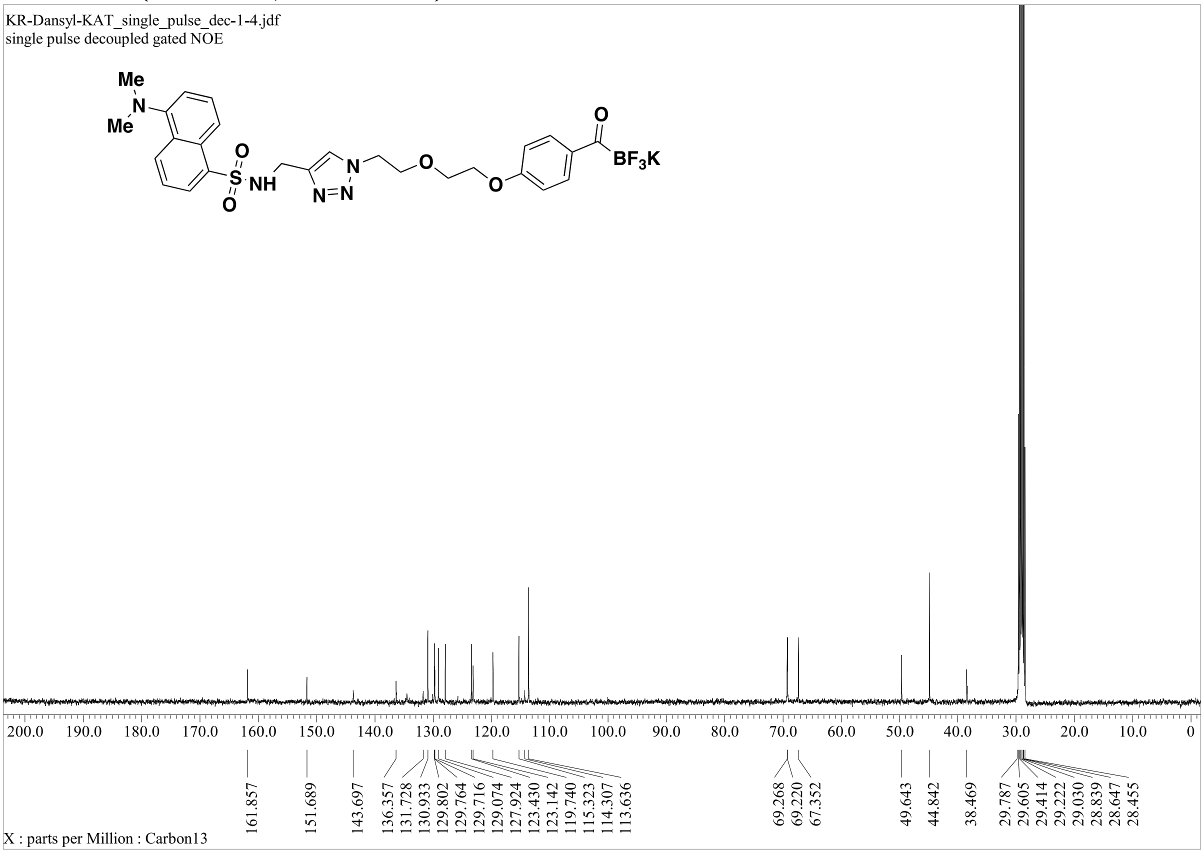

^{19}F NMR (376 MHz, acetone-*d6*)

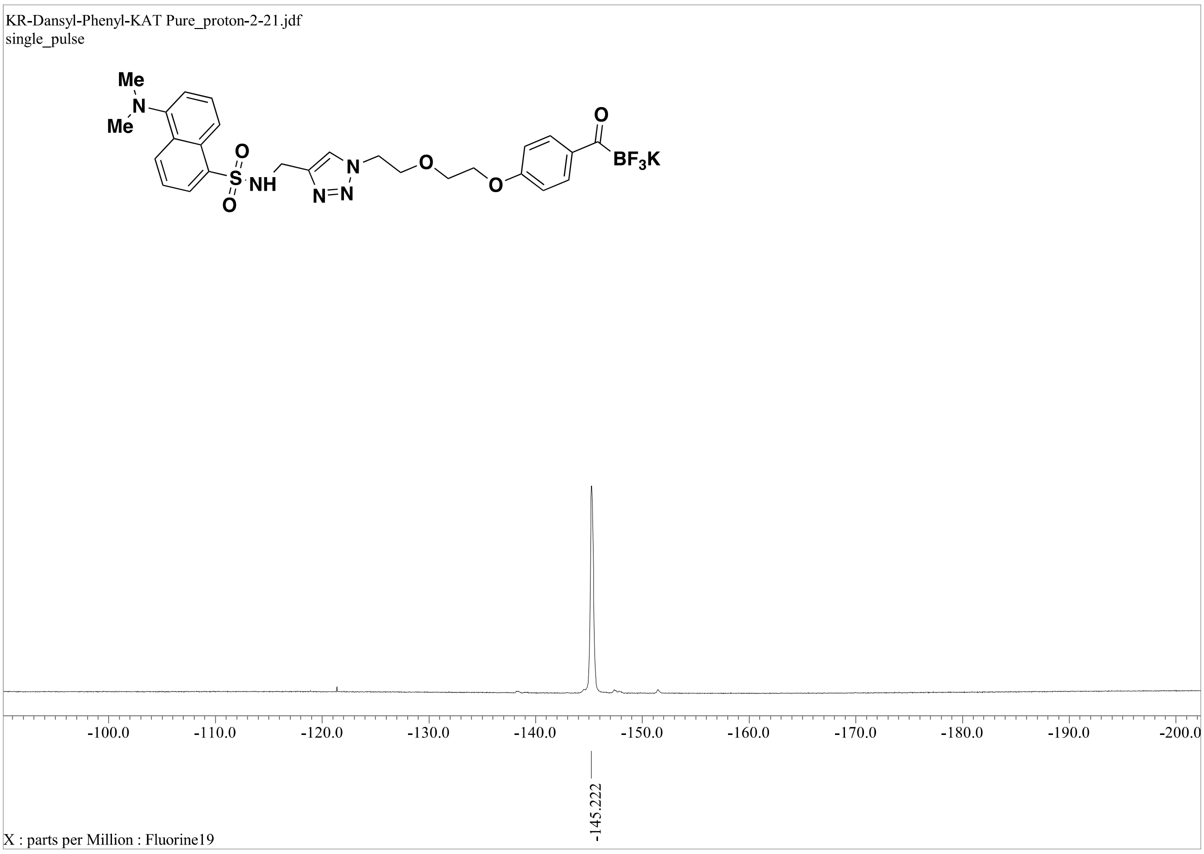

ESI-HRMS

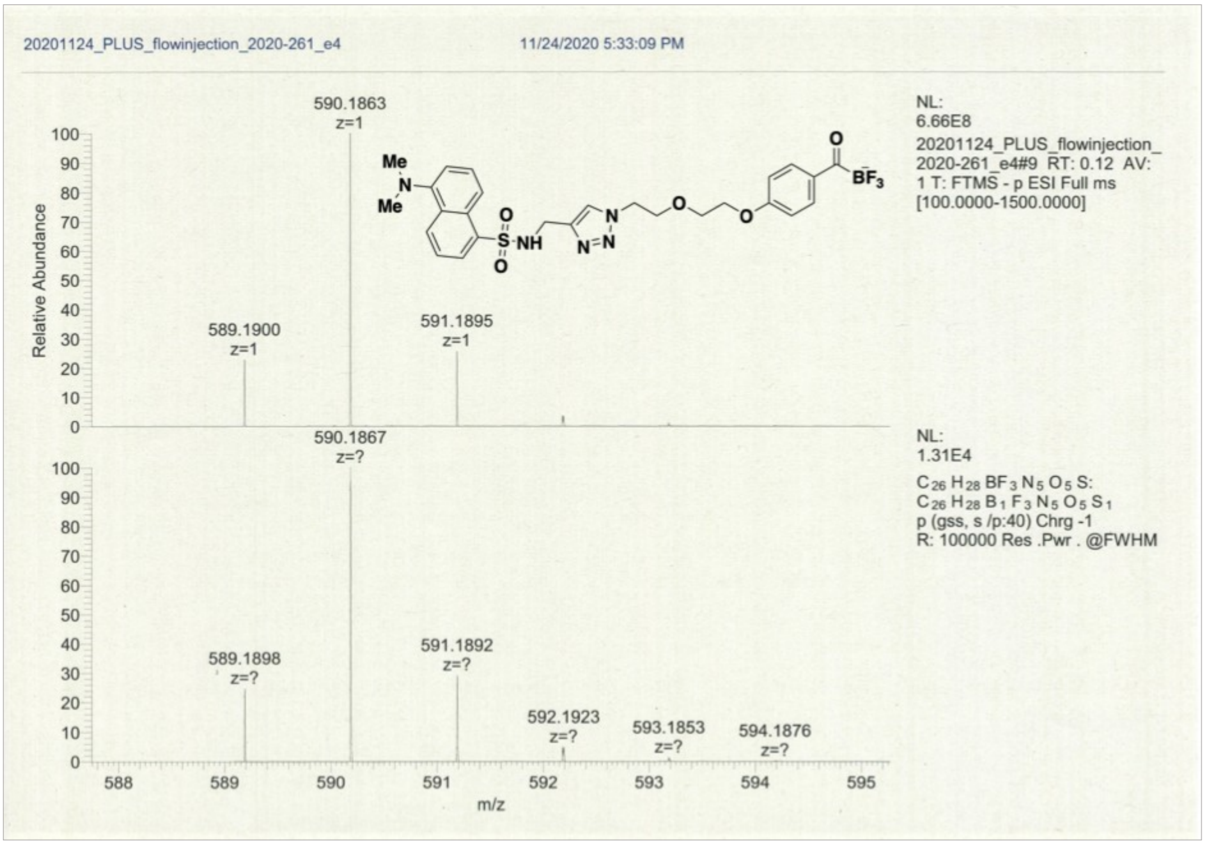

6.4 Synthesis of coumarin-KAT 16b

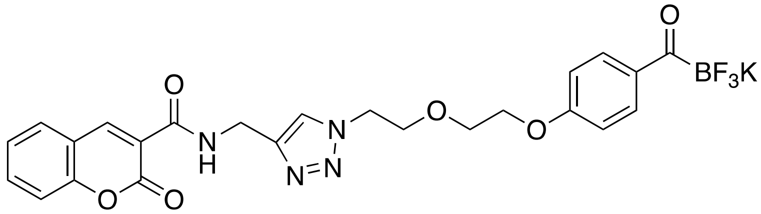

The product **16b** was synthesized according to the general procedure **6.2** using 2-oxo-*N*-(prop-2-yn-1-yl)-2*H*-chromene-3-carboxamide[8] **14b** (79.5 mg, 0.35 mmol, 1.0 equiv) and (4-(2-(2-azidoethoxy)ethoxy)phenyl)(trifluoro-λ4-boraneyl)methanone, potassium salt **15** (118 mg, 0.35 mmol, 1.0 equiv), CuI (66 mg, 0.35 mmol, 1.0 equiv) and Et_3N (97.5 μL, 0.7 mmol, 2.0 equiv) dissolved in 3.5 mL of 50% aqueous CH_3CN. White solid, isolated yield 160 mg (0.28 mmol, 81%). ^1H NMR (400 MHz, acetone-*d6*): δ = 9.13 (s, 1H), 8.92 (s, 1H), 8.07 (d, *J* = 8.8 Hz, 2H), 7.95–7.93 (m, 2H), 7.73 (dd, *J* = 8.8, 1.4 Hz, 2H), 7.45 (t, *J* = 8.0 Hz, 2H), 6.89 (d, *J* = 9.2 Hz, 2H), 4.66 (d, *J* = 5.6 Hz, 2H), 4.59 (t, *J* = 4.8 Hz, 2H), 4.17 (t, *J* = 4.8 Hz, 2H), 3.96 (t, *J* = 5.6 Hz, 2H), 3.84 (t, *J* = 4.8 Hz, 2H). ^{13}C NMR (100 MHz, acetone-*d6*): δ = 161.3, 154.6, 148.1, 134.1, 130.5, 130.3, 125.2, 116.3, 113.4, 69.5, 69.4, 67.2, 49.9, 35.2. ^{19}F NMR (376 MHz, acetone-*d6*): δ = –144.1. HRMS (ESI neg.): Exact mass calcd $C_{24}H_{21}BF_3N_4O_6$[M–K]$^-$: 529.1516, Found 529.1505.

^1H NMR (400 MHz, acetone-*d6*)

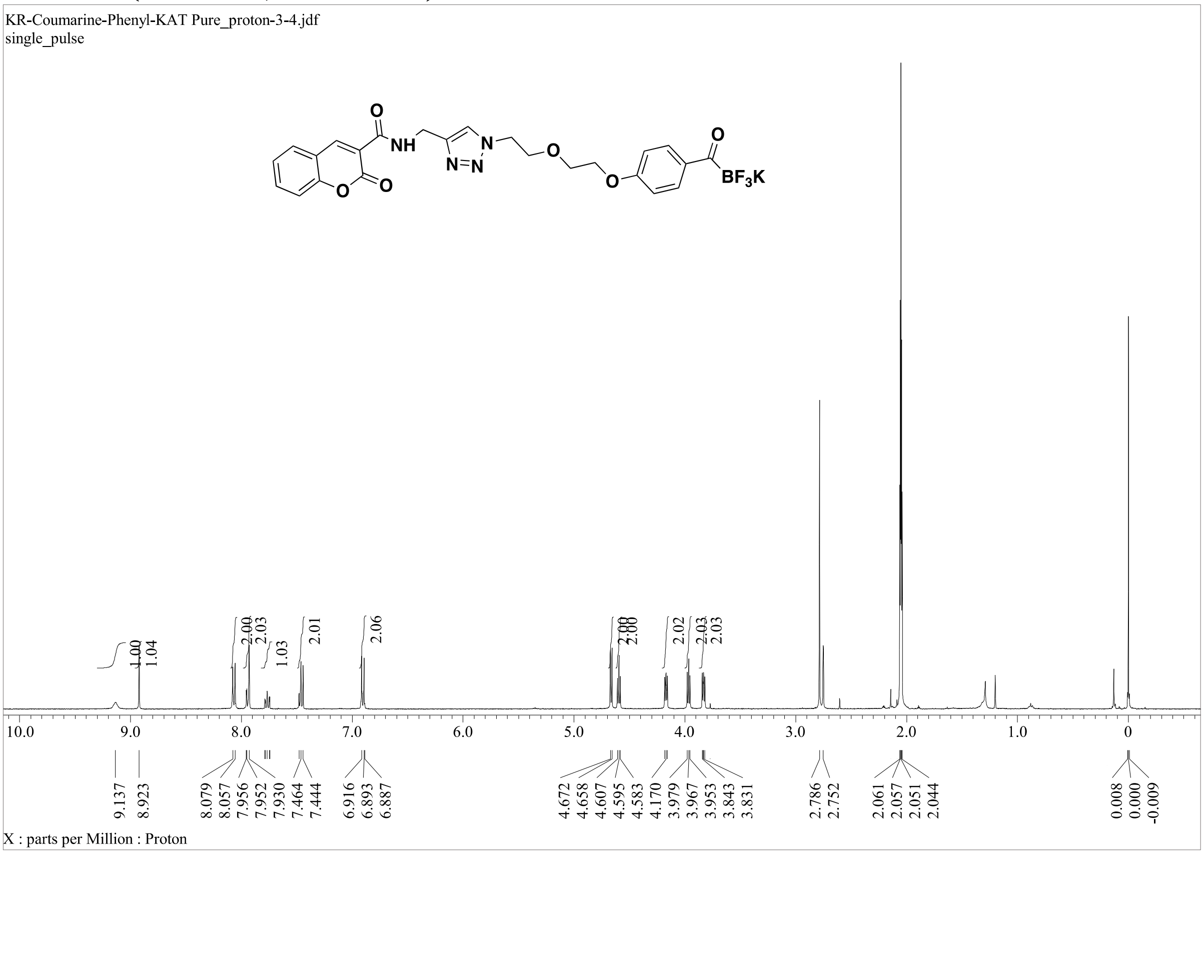

^{13}C NMR (100 MHz, acetone-*d6*)

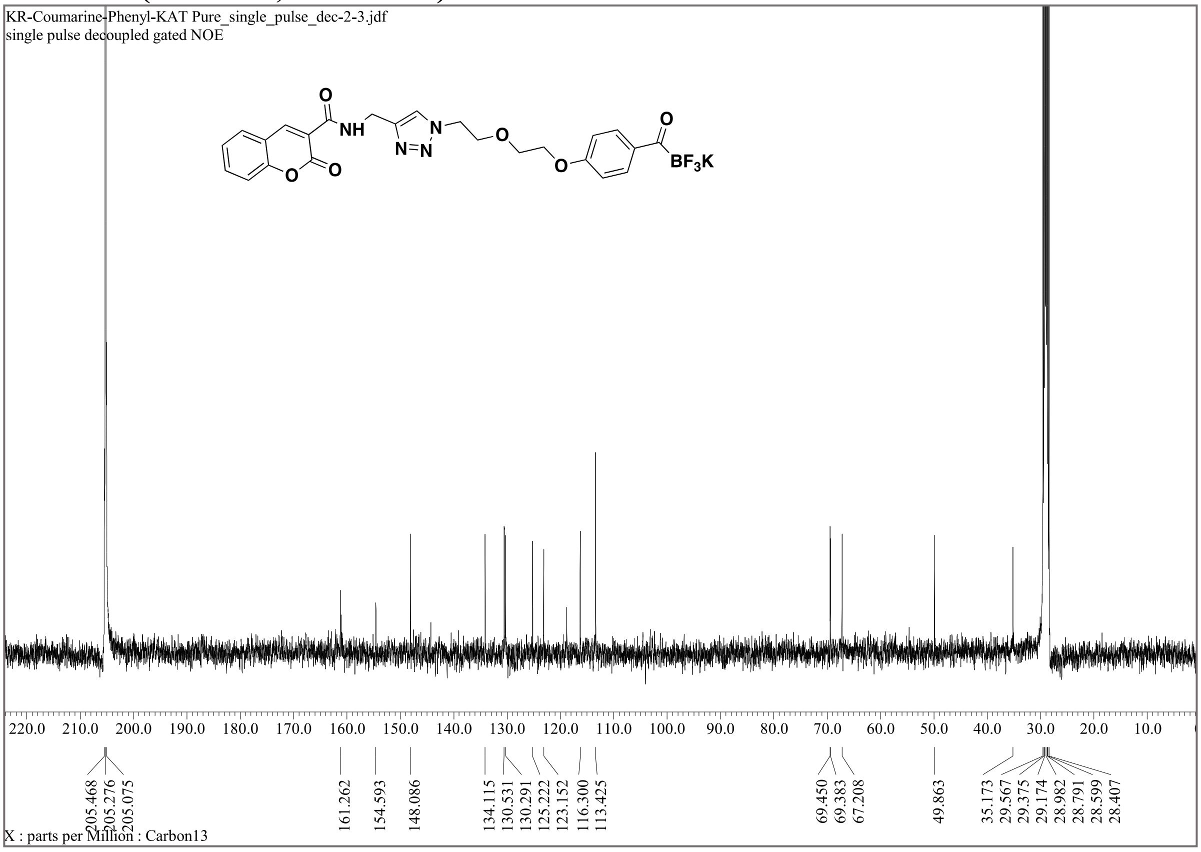

^{19}F NMR (376 MHz, acetone-*d6*)

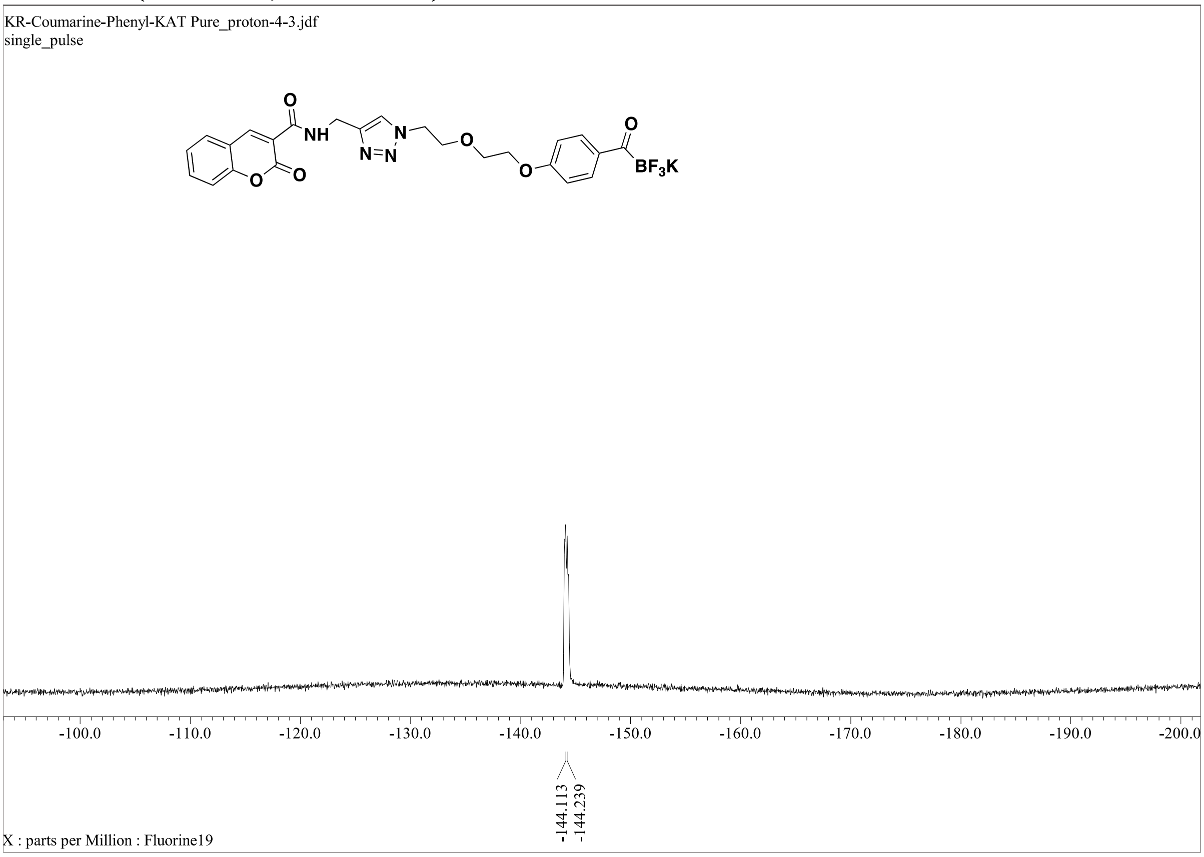

ESI-HRMS

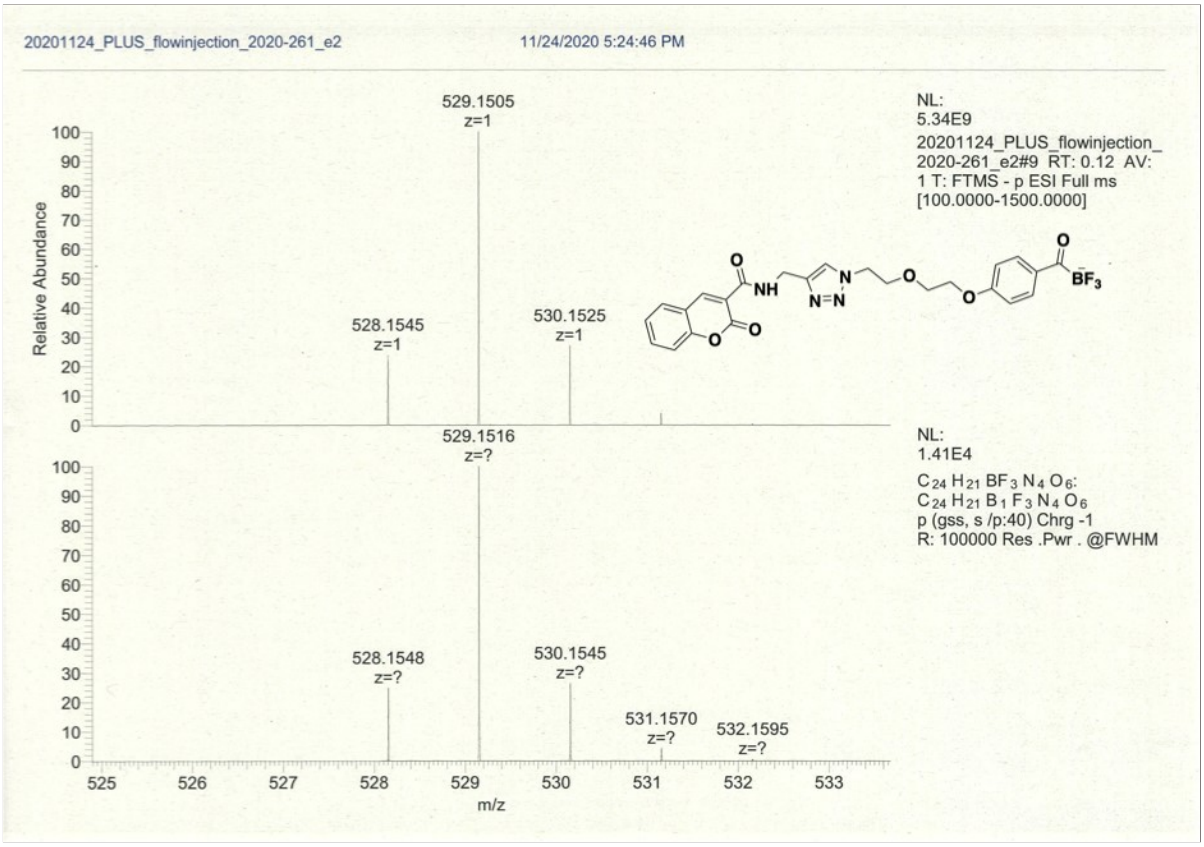

6.5 Synthesis of pyrene-KAT 16c

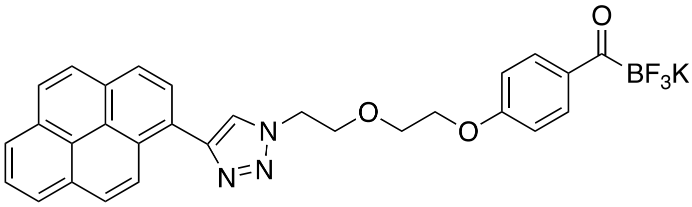

The product **16c** was synthesized according to the general procedure **6.2** using 1-ethynylpyrene **14c** (79.0 mg, 0.35 mmol, 1.0 equiv) and (4-(2-(2-azidoethoxy)ethoxy)phenyl)(trifluoro-λ4-boraneyl)methanone, potassium salt **15** (118 mg, 0.35 mmol, 1.0 equiv), CuI (66 mg, 0.35 mmol, 1.0 equiv) and Et_3N (97.5 µL, 0.7 mmol, 2.0 equiv) dissolved in 3.5 mL of 50% aqueous CH_3CN. White solid, isolated yield 142 mg (0.25 mmol, 72%). ^{1}H NMR (400 MHz, acetone-*d6*): δ = 8.93 (d, *J* = 7.6 Hz, 1H), 8.53 (s, 1H), 8.29–8.24 (m, 5H), 8.17–8.13 (m, 2H), 8.06–8.02 (m, 3H), 6.89 (*J* = 7.6 Hz. 2H), 4.80 (t, *J* = 4.0 Hz, 2H), 4.23 (t, *J* = 4.0 Hz, 2H), 4.11 (t, *J* = 4.8 Hz, 2H), 3.93 (t, *J* = 4.0 Hz, 2H). ^{13}C NMR (100 MHz, DMSO-*d6*): δ = 160.9, 146.6, 130.4, 127.9, 127.0, 125.7, 125.6, 125.4, 125.3, 113.9, 69.3, 67.6, 67.4, 50.2. ^{19}F NMR (376 MHz, acetone-*d6*): δ = 143.5. HRMS (ESI neg.): Exact mass calcd for $C_{29}H_{22}BF_3N_3O_3$ $[M–K]^-$: 528.1717, found 528.1707.

^{1}H NMR (400 MHz, acetone-*d6*)

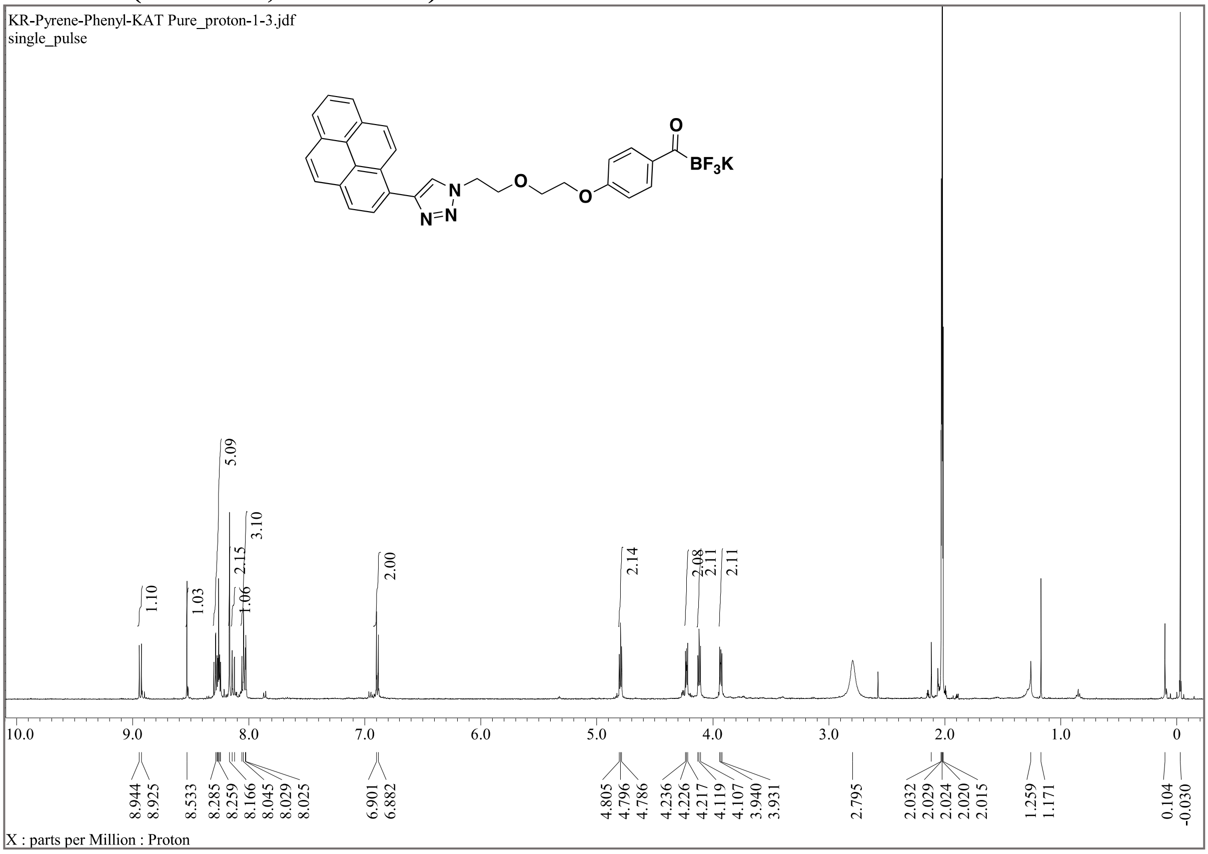

^{13}C NMR (100 MHz, DMSO-*d6*)

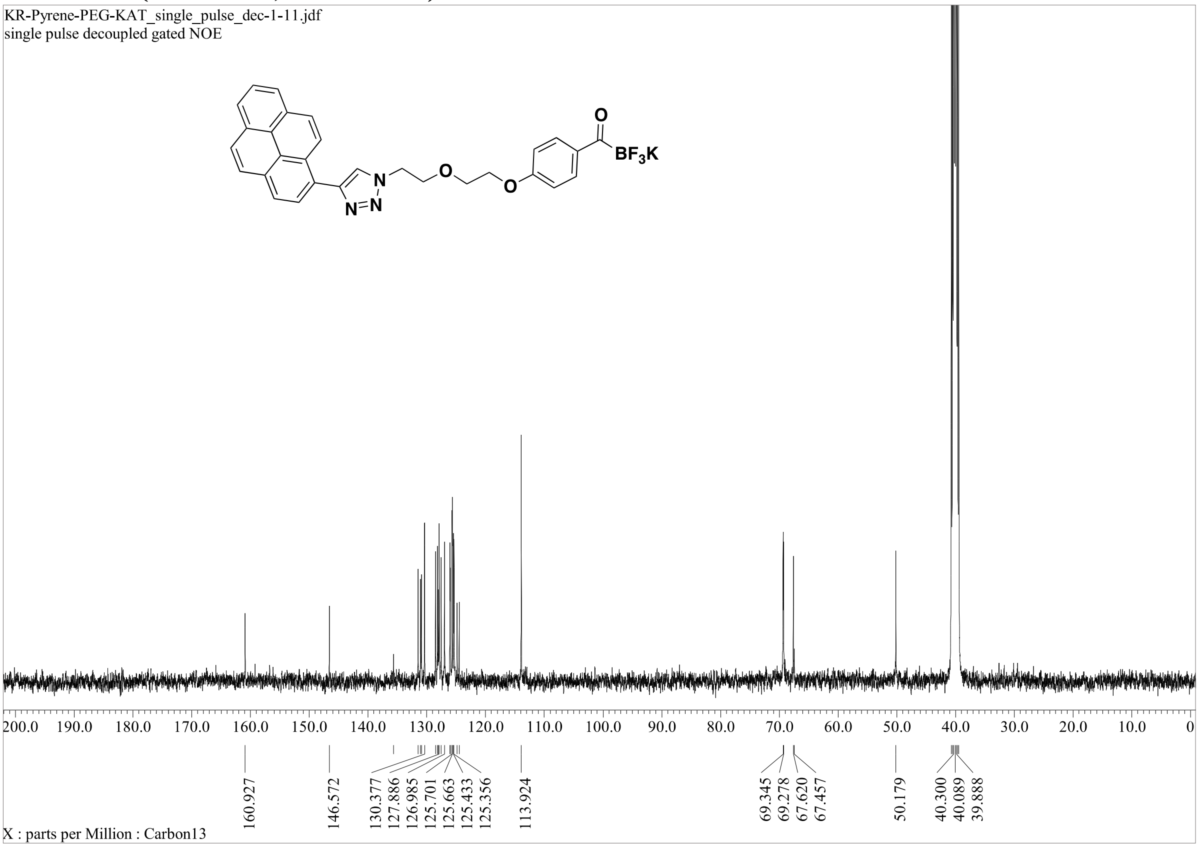

^{19}F NMR (376 MHz, acetone-*d6*)

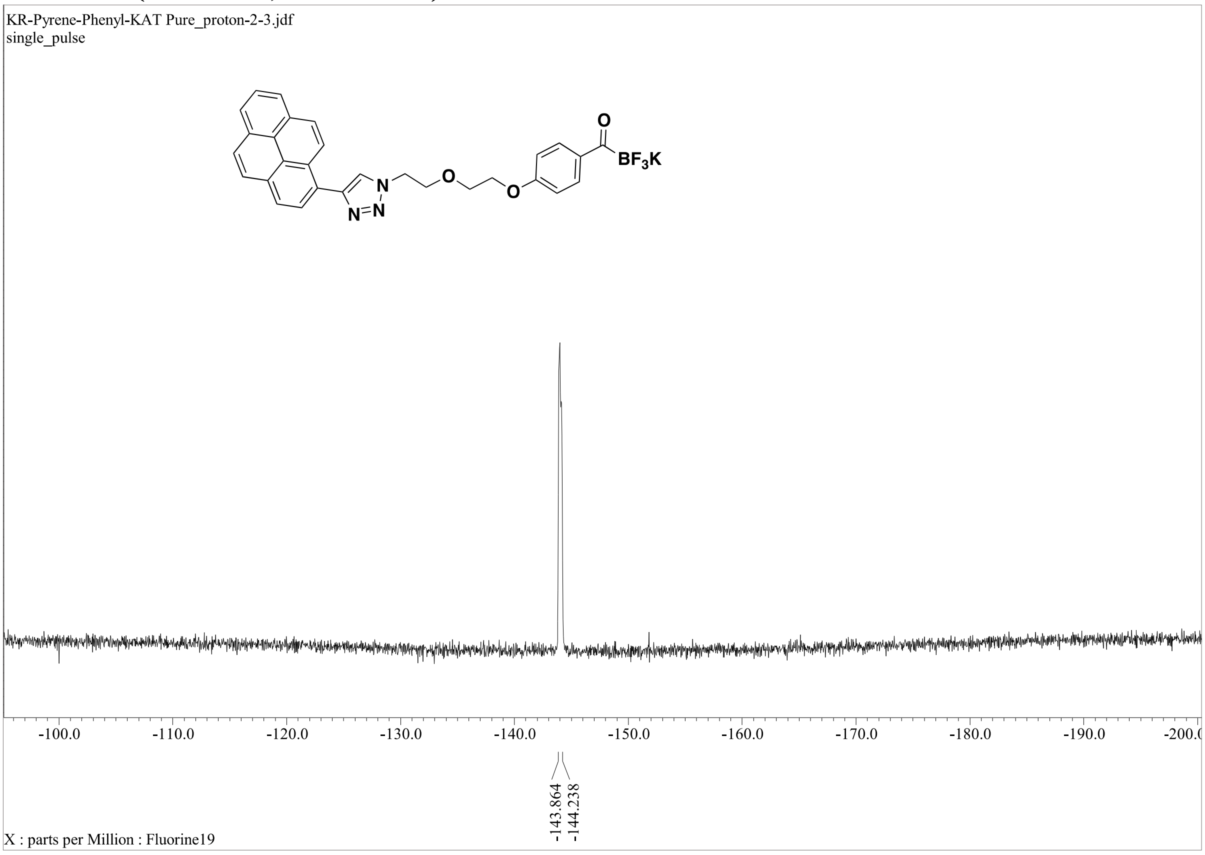

ESI-HRMS

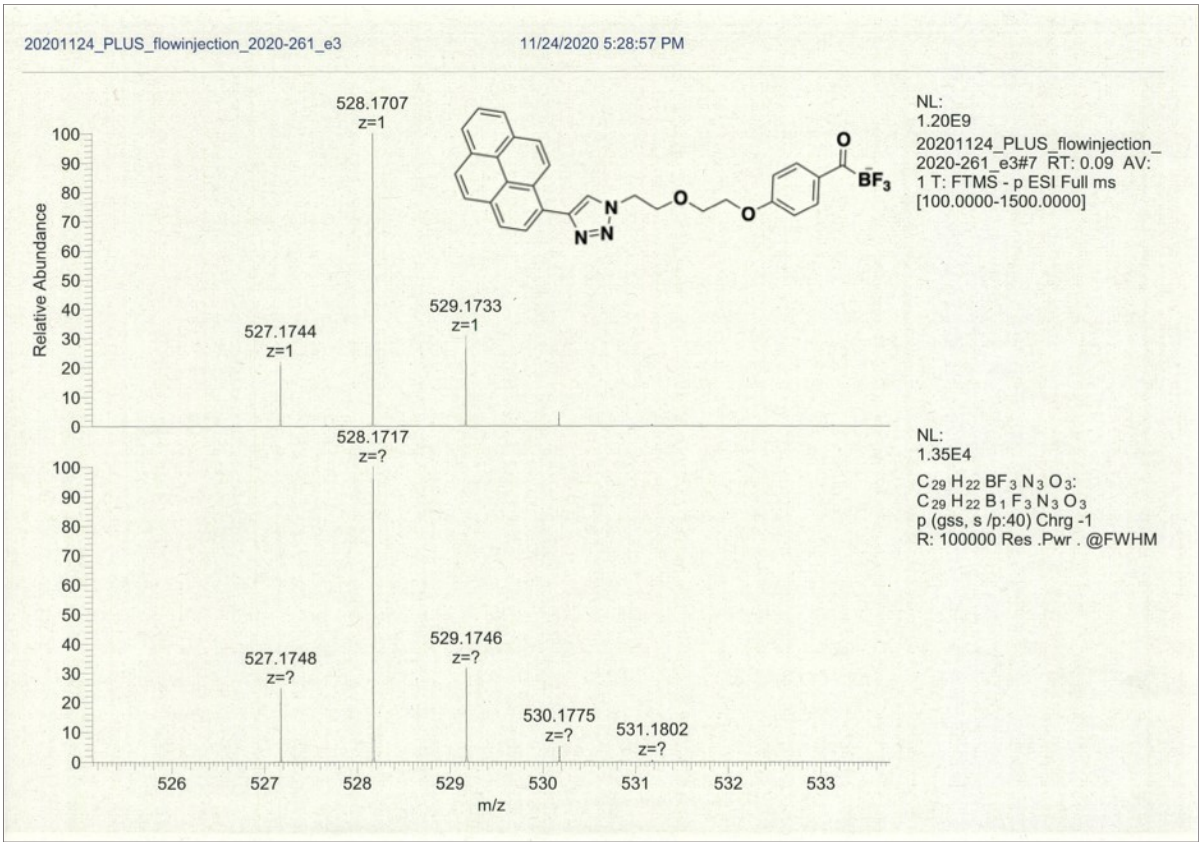

6.6 Synthesis of BODIPY-KAT 16d

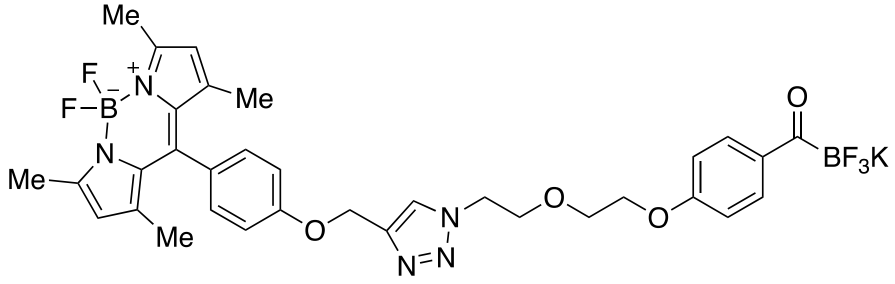

The product **16d** was synthesized according to the general procedure **6.2** using 5,5-difluoro-1,3,7,9-tetramethyl-10-(4-(prop-2-yn-1-yloxy)phenyl)-5*H*-4λ^4,5λ^4-dipyrrolo[1,2-*c*:2',1'-*f*][1,3,2]diazaborinine[9] **14d** (132.0 mg, 0.35 mmol, 1.0 equiv) and (4-(2-(2-azidoethoxy)ethoxy)phenyl)(trifluoro-λ4-boraneyl)methanone, potassium salt **15** (118 mg, 0.35 mmol, 1.0 equiv), CuI (66 mg, 0.35 mmol, 1.0 equiv) and Et_3N (97.5 µL, 0.7 mmol, 2.0 equiv) dissolved in 3.5 mL of 50% aqueous CH_3CN. Green solid, isolated yield 175 mg (0.26 mmol, 70%). ^{1}H NMR (400 MHz, DMSO-*d6*): δ = 8.2 (s, 1H), 7.83 (d, *J* = 8.8 Hz, 1H), 7.23 (d, *J* = 8.4 Hz, 2H), 7.16 (d, *J* = 8.4 Hz, 2H), 6.85 (d, *J* = 9.2 Hz, 1H), 6.13 (s, 2H), 5.12 (s, 2H), 4.55 (t, *J* = 5.2 Hz, 2H), 4.06 (t, *J* = 4.8 Hz, 2H), 3.86 (t, *J* = 4.8 Hz, 2H), 3.73 (t, *J* = 4.8 Hz, 2H), 2.41 (s, 6H), 1.36 (s, 6H). ^{13}C NMR (100 MHz, DMSO-*d6*): δ = 160.9, 159.3, 155.2, 143.3, 142.7, 131.6, 130.4, 129.7, 126.8, 125.7, 121.8, 116.0, 113.9, 69.3, 69.2, 67.4, 61.7, 49.9, 14.7. ^{19}F NMR (376 MHz, DMSO-*d6*): δ = –141.2, –143.6; HRMS (ESI neg.): Exact mass calcd for $C_{33}H_{33}B_2F_5N_5O_4$ $[M–K]^-$: 680.2655, found 680.2648.

^{1}H NMR (400 MHz, DMSO-*d6*)

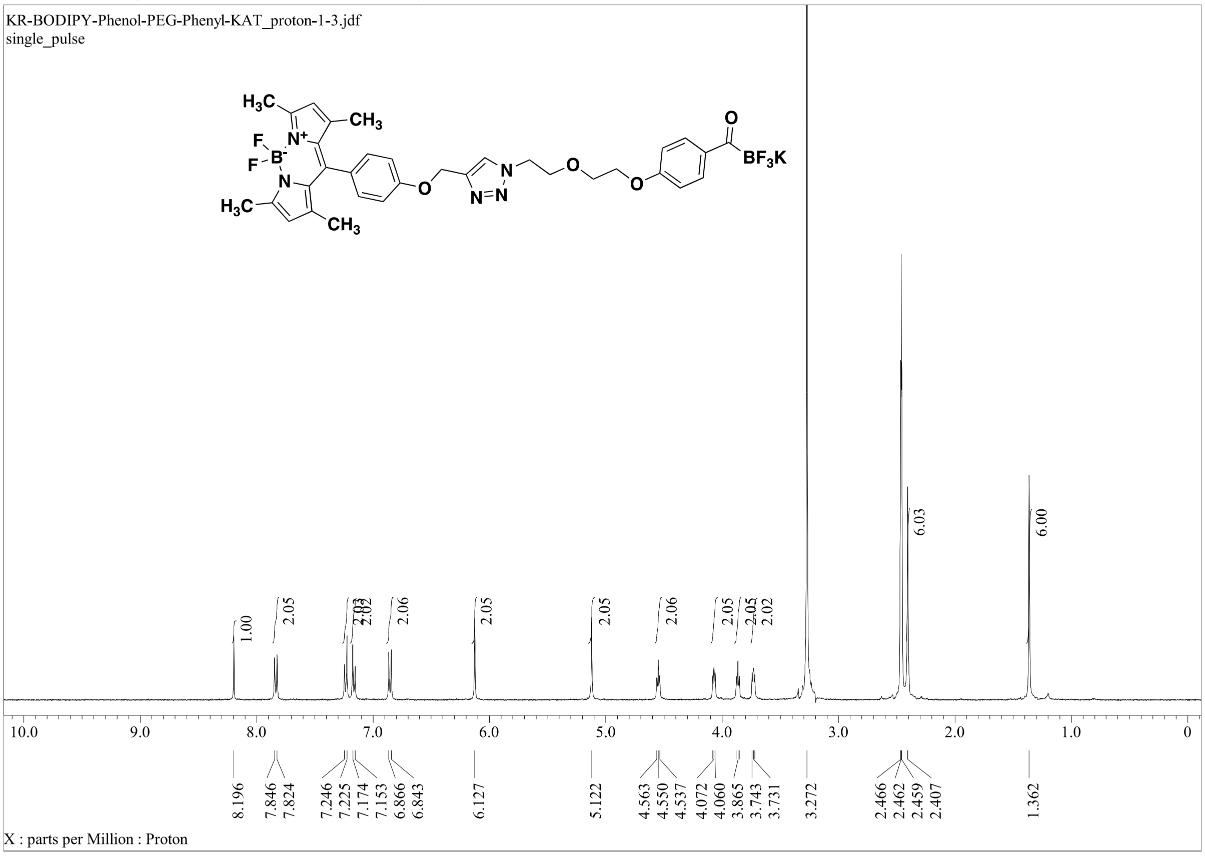

^{13}C NMR (100 MHz, DMSO-*d6*)

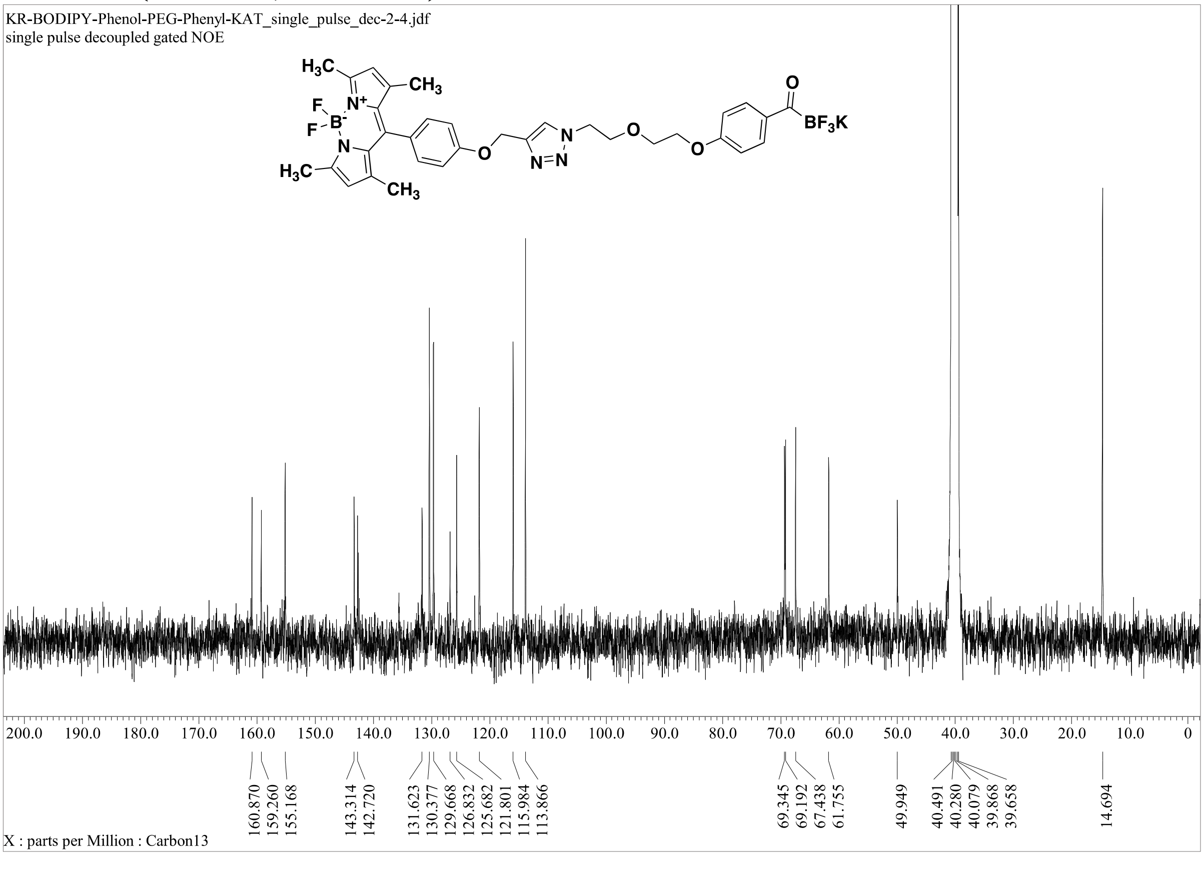

^{19}F NMR (376 MHz, DMSO-*d6*)

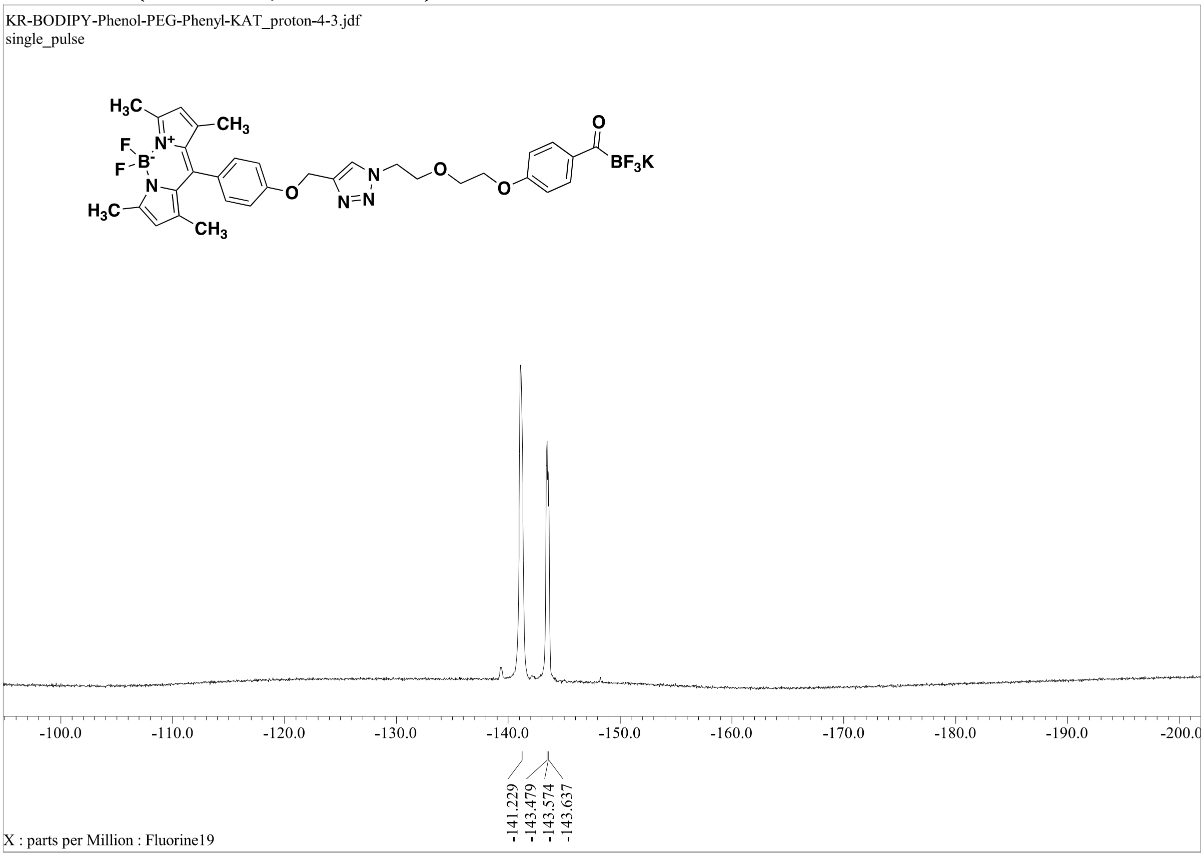

ESI-HRMS

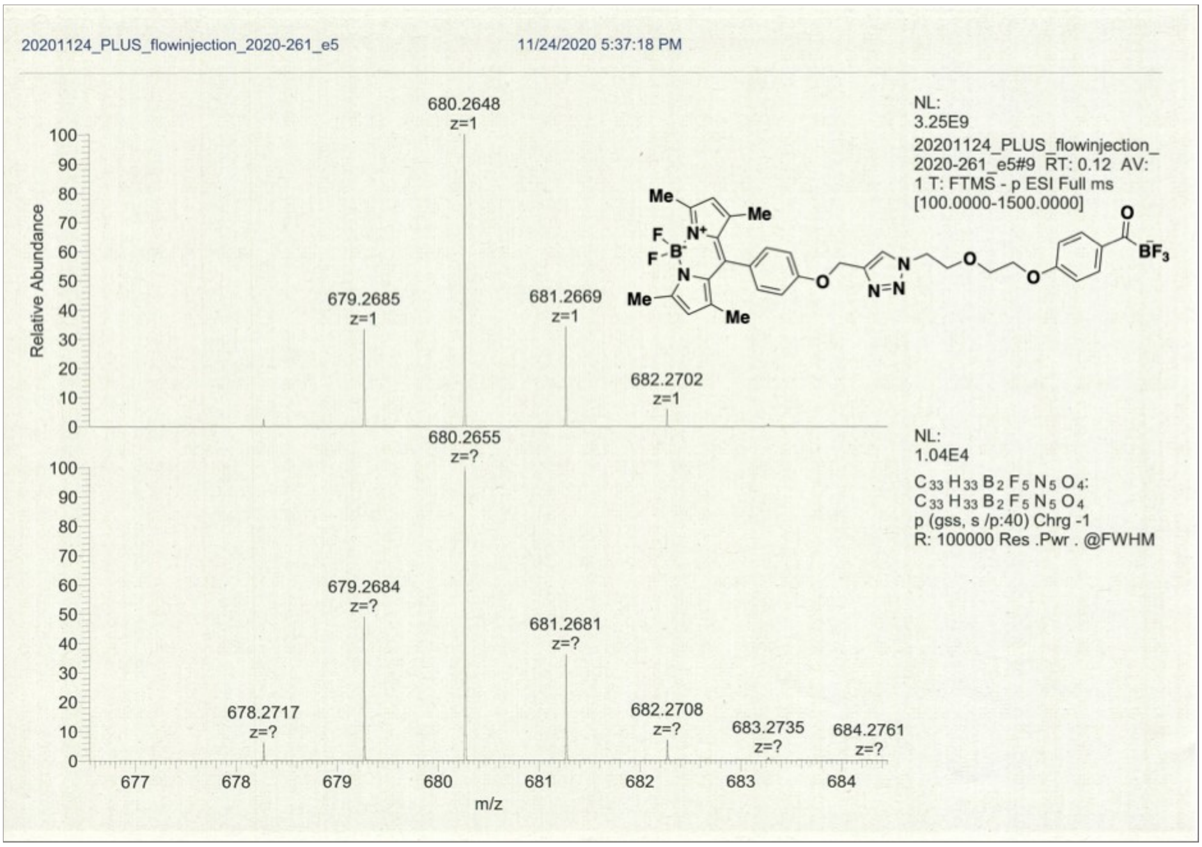

6.7 Synthesis of Rhodamine-KAT 16e

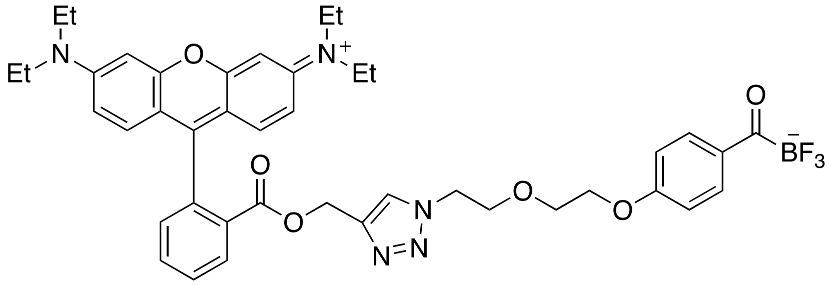

The product **16e** was synthesized according to the general procedure **6.2** using prop-2-yn-1-yl 2-(3-(diethyl-λ4-azaneylidene)-6-(diethylamino)-3*H*-xanthen-9-yl)benzoate[10] **14e** (168.0 mg, 0.35 mmol, 1.0 equiv) and (4-(2-(2-azidoethoxy)ethoxy)phenyl)(trifluoro-λ4-boraneyl)methanone, potassium salt **15** (118 mg, 0.35 mmol, 1.0 equiv), CuI (66 mg, 0.35 mmol, 1.0 equiv) and Et_3N (97.5 µL, 0.7 mmol, 2.0 equiv) dissolved in 3.5 mL of 50% aqueous CH_3CN. Red solid, isolated yield 183 mg (0.26 mmol, 64%). ^1H NMR (400 MHz, DMSO-*d6*): δ = 8.21 (dd, *J* = 8.0, 1.2 Hz, 1H), 7.92–7.72 (m, 5H), 7.48 (d, *J* = 8.8 Hz, 1H), 7.00 (dd, *J* = 8.4, 1.6 Hz, 2H), 6.95–6.90 (m, 4H), 6.84–6.81 (m, 2H), 5.04 (s, 2H), 4.48 (t, *J* = 5.2 Hz, 2H), 4.07 (t, *J* = 4.4 Hz, 2H), 3.83 (t, *J* = 4.8 Hz, 2H), 3.72 (t, *J* = 4.8 Hz, 2H), 3.63 (q, *J* = 7.6 Hz, 8H), 1.21 (t, *J* = 7.6 Hz, 12H). ^{13}C NMR (100 MHz, DMSO-*d6*): δ = 165.0, 160.8, 157.9, 157.6, 155.6, 141.2, 135.7, 133.8, 131.3, 131.0, 130.3, 129.7, 125.4, 115.0, 114.7, 113.8, 113.4, 96.4, 69.1, 67.4, 58.7, 49.8, 45.8 12.9; ^{19}F NMR (376 MHz, DMSO-*d6*): δ = –145.19. HRMS (ESI neg.): Exact mass calcd for $C_{42}H_{45}BF_3KN_5O_6[M]^+$: 822.3054, found 822.3036.

^{1}H NMR (400 MHz, DMSO-*d6*)

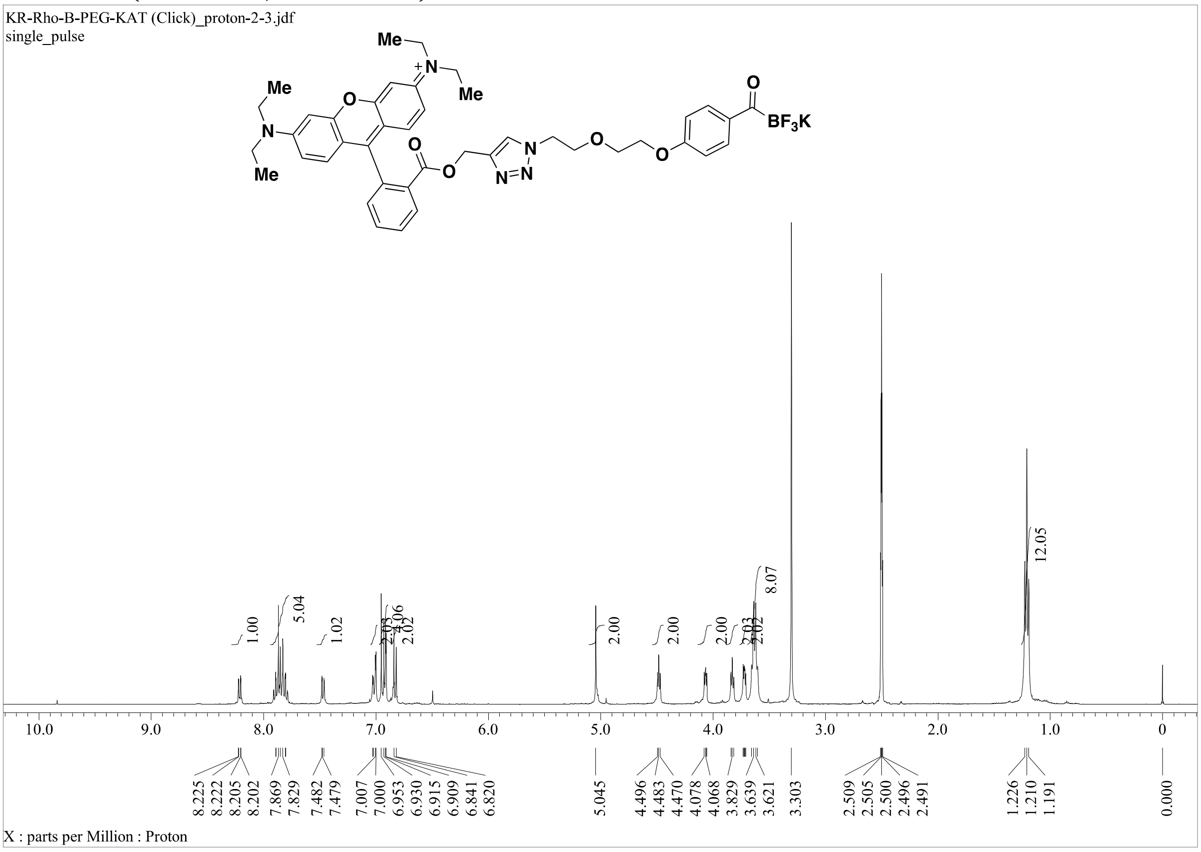

^{13}C NMR (100 MHz, DMSO-*d6*)

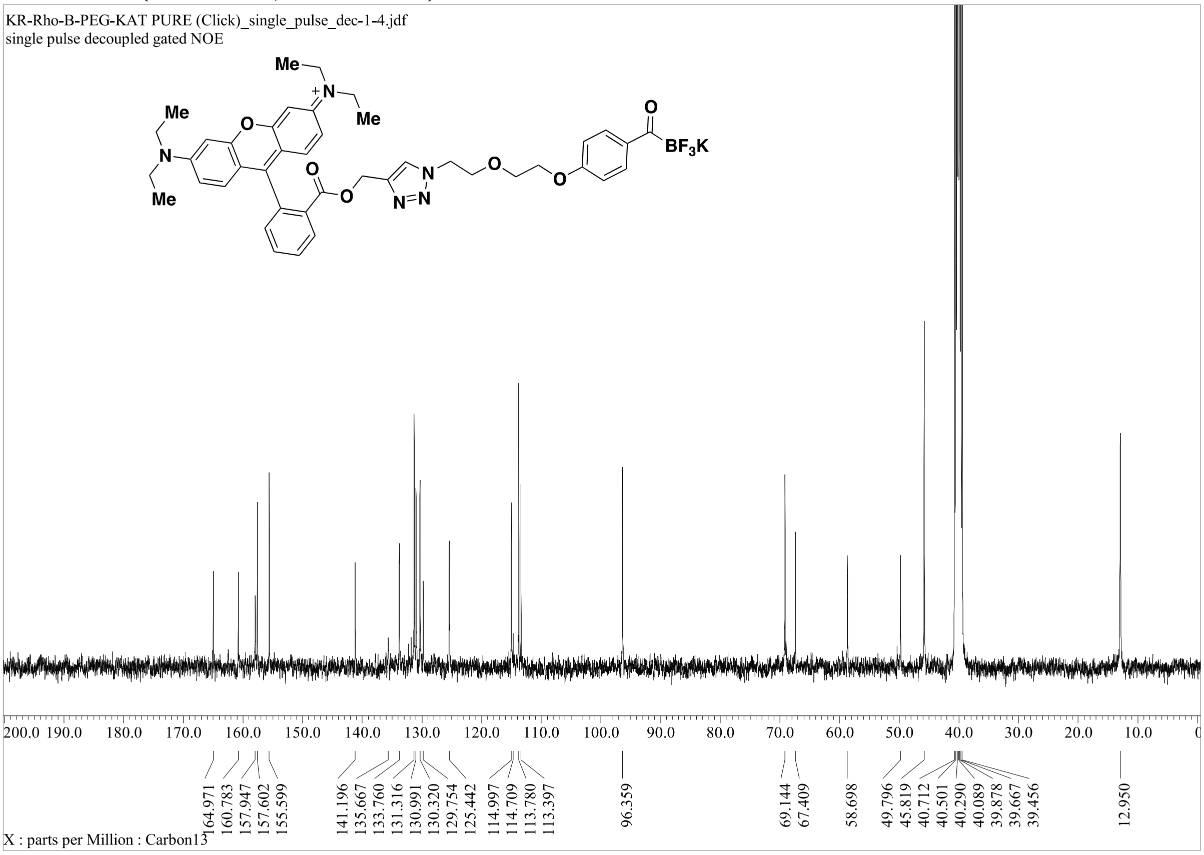

^{19}F NMR (376 MHz, DMSO-*d6*)

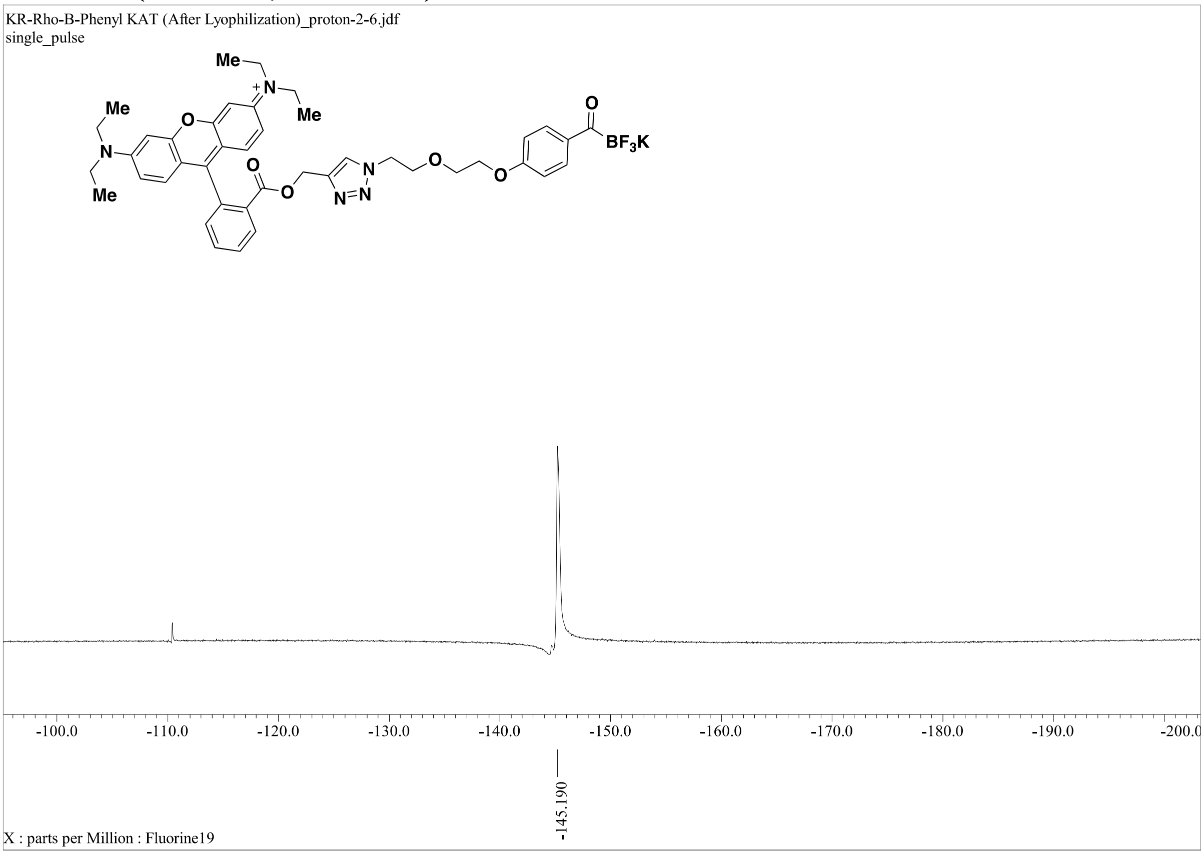

ESI-HRMS

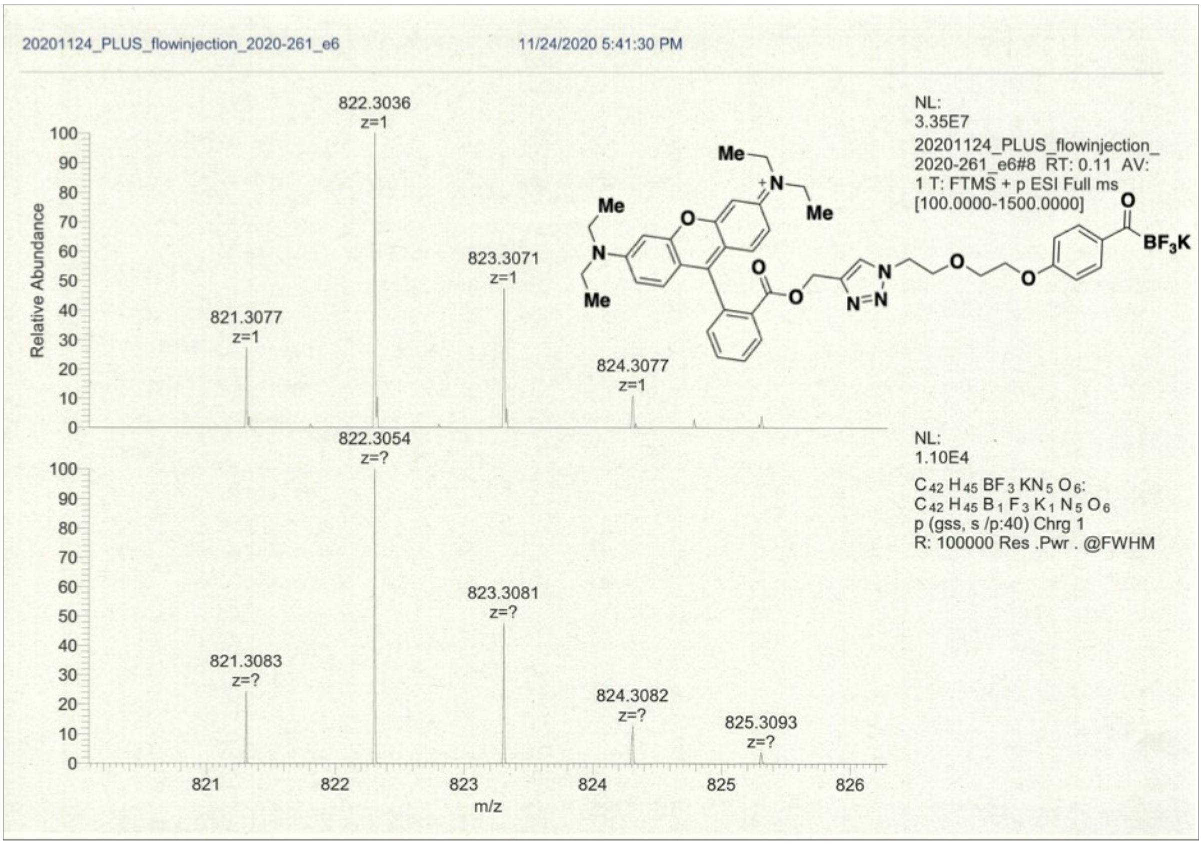

6.8 UV absorption spectra of Dye-KATs

The UV absorption was measured in 50% aqueous CH_3CN at room temperature with 50 μM potassium acyltrifluoroborates **15, 16a–e**. The observed intense absorption for compound **15** at λ (max) = 275 nm, **16a** at λ (max) = 266 nm, **16b** at λ (max) = 286 nm, **16c** at λ (max) = 350 nm, **16d** at λ (max) = 499 nm, **16e** at λ (max) = 562 nm.

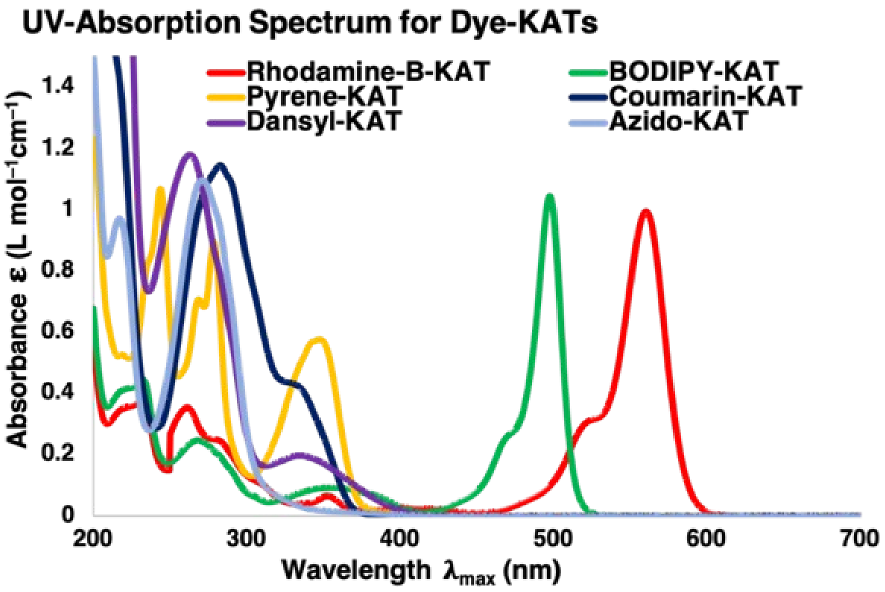

7. Protein functionalization by KAT ligation

7.1 General Experimental Procedure for Fluorescent Labelling of EPF Proteins by KAT Ligation

Functionalized EPF proteins **2b**, **7b**, and **13b** (1.0 equiv) was dissolved in 50% aqueous CH_3CN mixture with 0.1% TFA. The reaction mixture was placed in a closed UV chamber and irradiated with 365 nm UV light at room temperature under dark to deprotect the photo labile group. The photo deprotection was monitored by analytical RP-HPLC. After completion of the deprotection, the dye potassium acyltrifluoroborate (KATs) **15**, **16a–e** (1.2 equiv) was directly added to the reaction mixture stirred at room temperature for 20 min. The dye conjugated folded EPFs **17a–f**, **18a–f** and **19a–f** were identified in analytical RP-HPLC, and MALDI-TOF. The crude mixture was purified by RP-HPLC.

7.2 Synthesis of dansyl-EPFL9 17a

The dansyl conjugated EPFL9 **17a** was synthesized according to general procedure **7.1** using folded photo-protected hydroxylamine EPFL9 **2b** (1 mg, 0.18 μM) and dansyl KAT **16a** (0.14 mg, 0.22 μM). The crude mixture was purified by preparative RP-HPLC using Phenomenex

Jupiter® C18 column (5 µm, 300 Å pore size, 21.2 mm I.D. × 250 mm) with a gradient of 20–80% CH_3CN with 0.1% TFA in 30 min, flow rate 10 mL/min. The fractions containing compounds pooled and lyophilized to give pure **17a** (0.54 mg, 52% yield). The purity and identity of the **17a** was confirmed using analytical RP-HPLC and ESI-HRMS. The m/z calculated for **17a** $C_{244}H_{366}N_{76}O_{73}S_7$ $[M+4H]^{4+}$: 1438.1322 Da, measured: 1438.1313 Da.

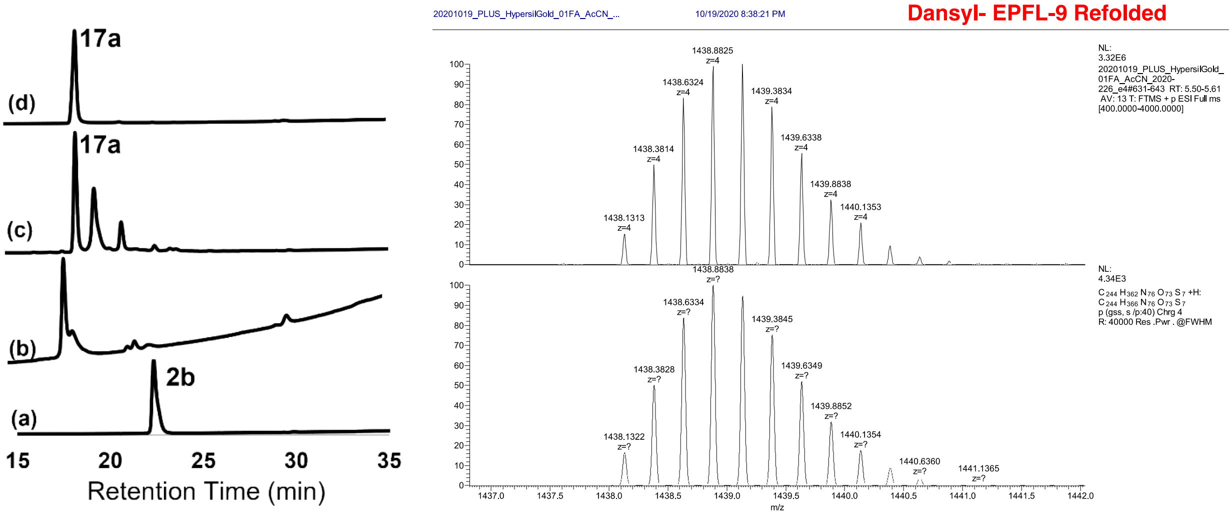

a) Photo-deprotection at 0 min; b) Photo-deprotection at 30 min; c) KAT Ligation at 20 min; d) Purified **17a**

7.3 Synthesis of coumarin-EPFL9 17b

The coumarin conjugated EPFL9 **17b** was synthesized according to general procedure **7.1** using folded photo-protected hydroxylamine EPFL9 **2b** (1 mg, 0.18 µM) and coumarin KAT **16b** (0.12 mg, 0.22 µM). The crude mixture was purified by preparative RP-HPLC using Phenomenex Jupiter® C18 column (5 µm, 300 Å pore size, 21.2 mm I.D. × 250 mm) with a gradient of 20–80% CH_3CN with 0.1% TFA in 30 min, flow rate 10 mL/min. The fractions containing compounds pooled and lyophilized to give pure **17b** (0.62 mg, 60% yield). The purity and identity of the **17b** was confirmed using analytical RP-HPLC and ESI-HRMS. The m/z calculated for **17b** $C_{242}H_{359}N_{75}O_{74}S_6$ $[M+4H]^{4+}$: 1422.8734 Da, measured 1422.8705 Da.

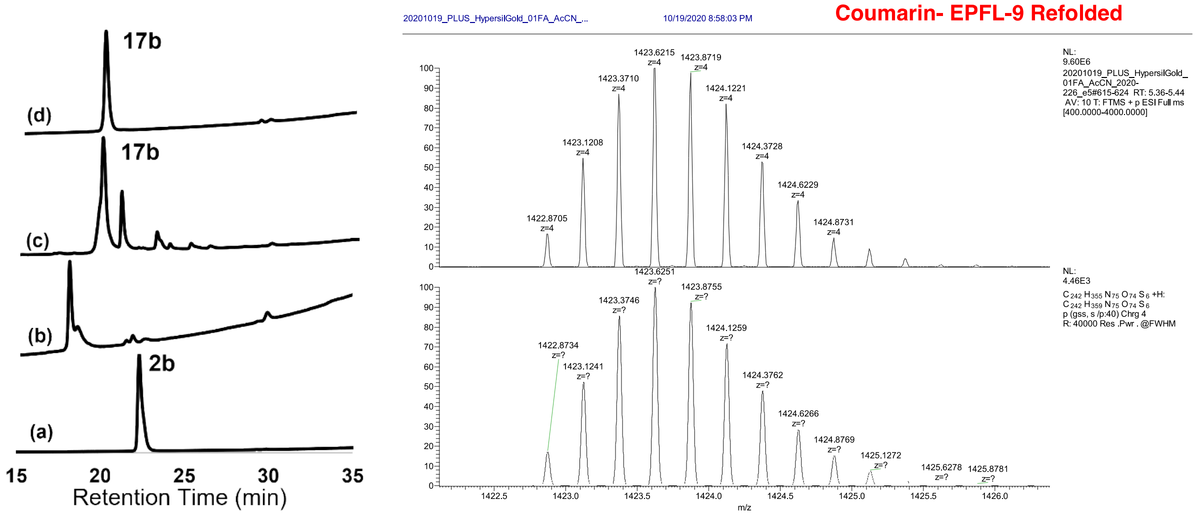

a) Photo-deprotection at 0 min; b) Photo-deprotection at 30 min; c) KAT Ligation at 20 min; d) Purified **17b**

7.4 Synthesis of pyrene-EPFL9 17c

The pyrene conjugated EPFL9 **17c** was synthesized according to general procedure **7.1** using folded photo-protected hydroxylamine EPFL9 **2b** (1 mg, 0.18 µM) and pyrene KAT **16c** (0.12 mg, 0.22 µM). The crude mixture was purified by preparative RP-HPLC using Phenomenex Jupiter® C18 column (5 µm, 300 Å pore size, 21.2 mm I.D. × 250 mm) with a gradient of 20–80% CH_3CN with 0.1% TFA in 30 min, flow rate 10 mL/min. The fractions containing compounds pooled and lyophilized to give pure **17c** (0.72 mg, 70% yield). The purity and identity of the **17c** was confirmed using analytical RP-HPLC and ESI-HRMS. The m/z calculated for **17c** $C_{247}H_{360}N_{74}O_{71}S_6$ $[M+4H]^{4+}$: 1422.6284 Da, measured: 1422.6269 Da.

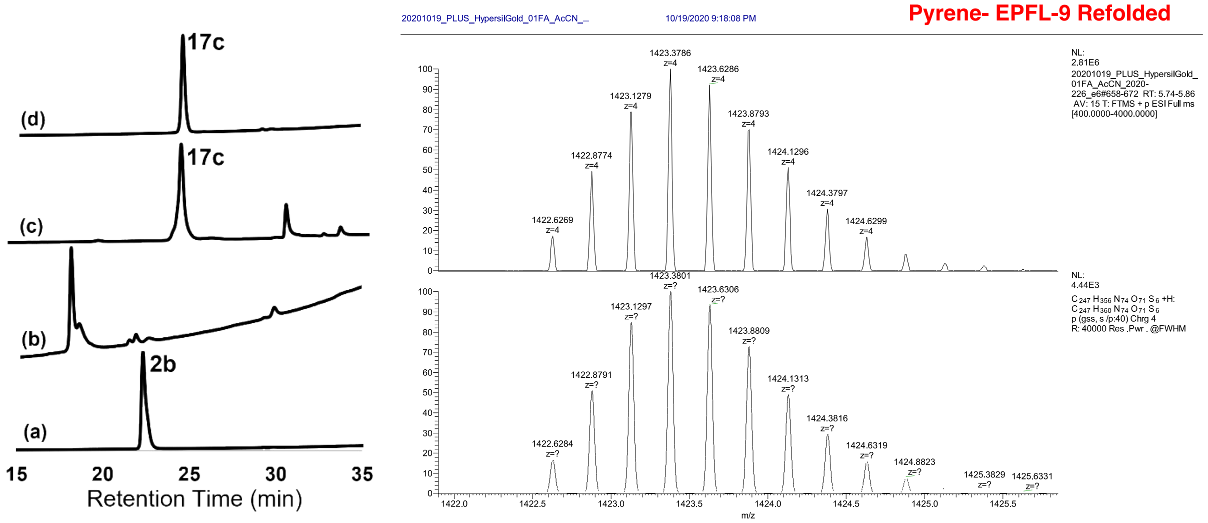

a) Photo-deprotection at 0 min; b) Photo-deprotection at 30 min; c) KAT Ligation at 20 min; d) Purified **17c**

7.5 Synthesis of BODIPY-EPFL9 17d

The BODIPY conjugated EPFL9 **17d** was synthesized according to general procedure **7.1** using folded photo-protected hydroxylamine EPFL9 **2b** (1 mg, 0.18 µM) and BODIPY KAT **16d** (0.16 mg, 0.22 µM). The crude mixture was purified by preparative RP-HPLC using Phenomenex Jupiter® C18 column (5 µm, 300 Å pore size, 21.2 mm I.D. × 250 mm) with a gradient of 20–80% CH_3CN with 0.1% TFA in 30 min, flow rate 10 mL/min. The fractions containing compounds pooled and lyophilized to give pure **17d** (0.8 mg, 85% yield). The purity and identity of the **17d** was confirmed using analytical RP-HPLC and ESI-HRMS. The m/z calculated for **17d** $C_{251}H_{371}BF_2N_{76}O_{72}S_6$ $[M+4H]^{4+}$: 1460.6524 Da, measured 1460.6505 Da.

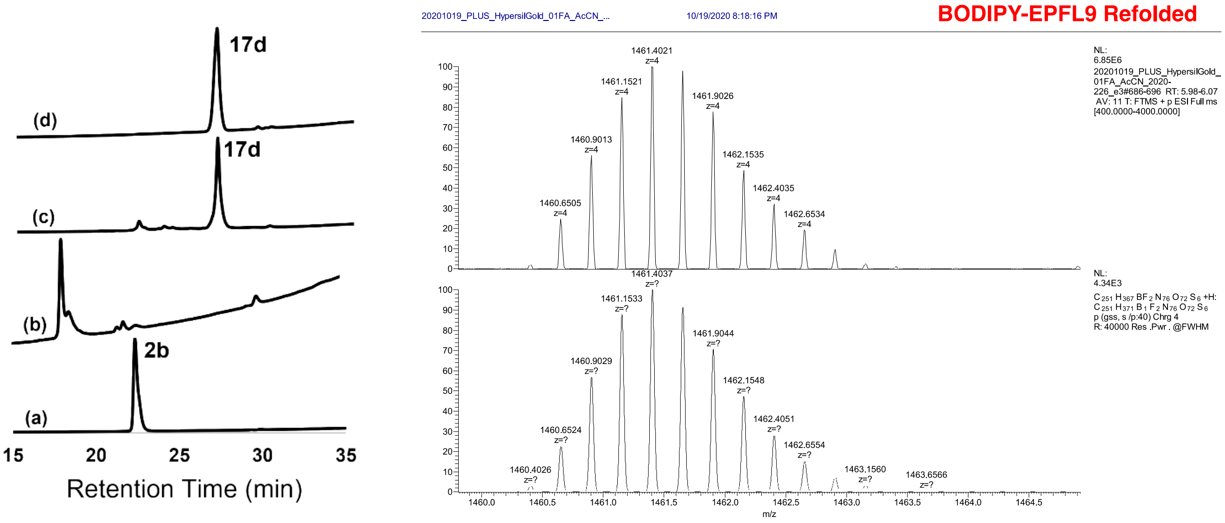

a) Photo-deprotection at 0 min; b) Photo-deprotection at 30 min; c) KAT Ligation at 20 min; d) Purified **17d**

7.6 Synthesis of Rhodamine-EPFL9 17e

The rhodamine-B conjugated EPFL9 **17e** was synthesized according to general procedure **7.1** using folded photo-protected hydroxylamine EPFL9 **2b** (1 mg, 0.18 µM) and rhodamine-B KAT **16e** (0.18 mg, 0.22 µM). The crude mixture was purified by preparative RP-HPLC using Phenomenex Jupiter® C18 column (5 µm, 300 Å pore size, 21.2 mm I.D. × 250 mm) with a gradient of 20–80% CH_3CN with 0.1% TFA in 30 min, flow rate 10 mL/min. The fractions containing compounds pooled and lyophilized to give pure **17e** (0.90 mg, 84% yield). The purity and identity of the **17e** was confirmed using analytical RP-HPLC and ESI-HRMS. The m/z calculated for **17e** $C_{260}H_{383}N_{76}O_{74}S_6$ $[M+4H]^{5+}$: 1189.1368 Da, measured: 1189.1352 Da.

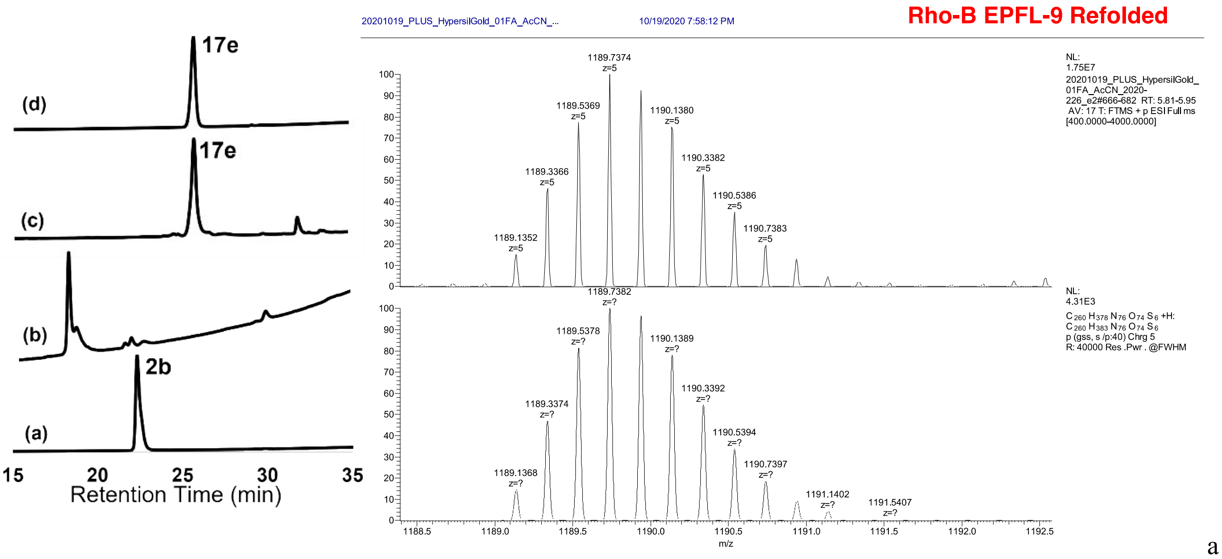

a) Photo-deprotection at 0 min; b) Photo-deprotection at 30 min; c) KAT Ligation at 20 min; d) Purified **17e**

7.7 Synthesis of Azido-EPFL9 17f

The azido conjugated EPFL9 **17f** was synthesized according to general procedure **7.1** using folded photo-protected hydroxylamine EPFL9 **2b** (1 mg, 0.18 μM) and azido KAT **15** (0.07 mg, 0.22 μM). The crude mixture was preparative RP-HPLC using Phenomenex Jupiter® C18 column (5 μm, 300 Å pore size, 21.2 mm I.D. × 250 mm) with a gradient of 20–80% CH_3CN with 0.1% TFA in 30 min, flow rate 10 mL/min. The fractions containing compounds pooled and lyophilized to give pure **17f** (0.74 mg, 75% yield). The purity and identity of the **17f** was confirmed using analytical RP-HPLC and ESI-HRMS. The m/z calculated for **17f** $C_{229}H_{352}N_{74}O_{71}S_6$ $[M+6H]^{6+}$: 911.0750 Da, measured 911.0786 Da.

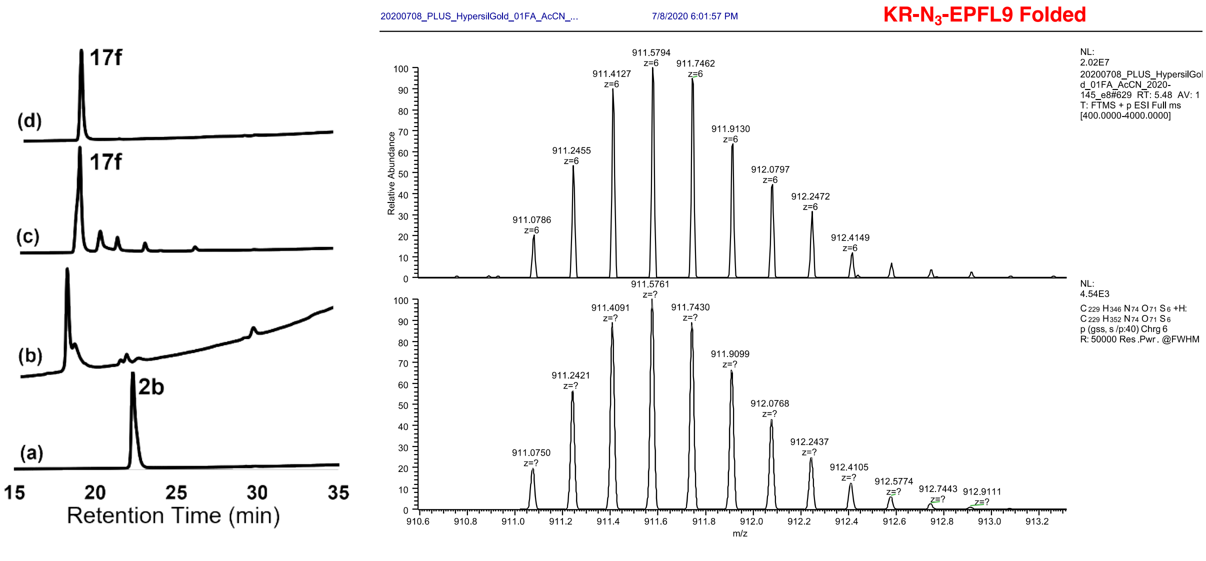

a) Photo-deprotection at 0 min; b) Photo-deprotection at 30 min; c) KAT Ligation at 20 min; d) Purified **17f**

7.8 Synthesis of dansyl-EPF2 18a

The dansyl conjugated EPF2 **18a** was synthesized according to general procedure **7.1** using folded photo-protected hydroxylamine EPF2 **7b** (1 mg, 0.165 μM) and danysl KAT **16a** (0.12 mg, 0.198 μM). The crude mixture was purified by preparative RP-HPLC using Phenomenex Jupiter® C18 column (5 μm, 300 Å pore size, 21.2 mm I.D. × 250 mm) with a gradient of 20–80% CH_3CN with 0.1% TFA in 30 min, flow rate 10 mL/min. The fractions containing compounds pooled and lyophilized to give pure **18a** (0.71 mg, 68% yield). The purity and identity of **18a** was confirmed using analytical RP-HPLC and ESI-HRMS. The m/z calculated for **18a** $C_{268}H_{404}N_{77}O_{80}S_9$ $[M+4H]^{4+}$: 1567.9403 Da, measured 1567.9398 Da.

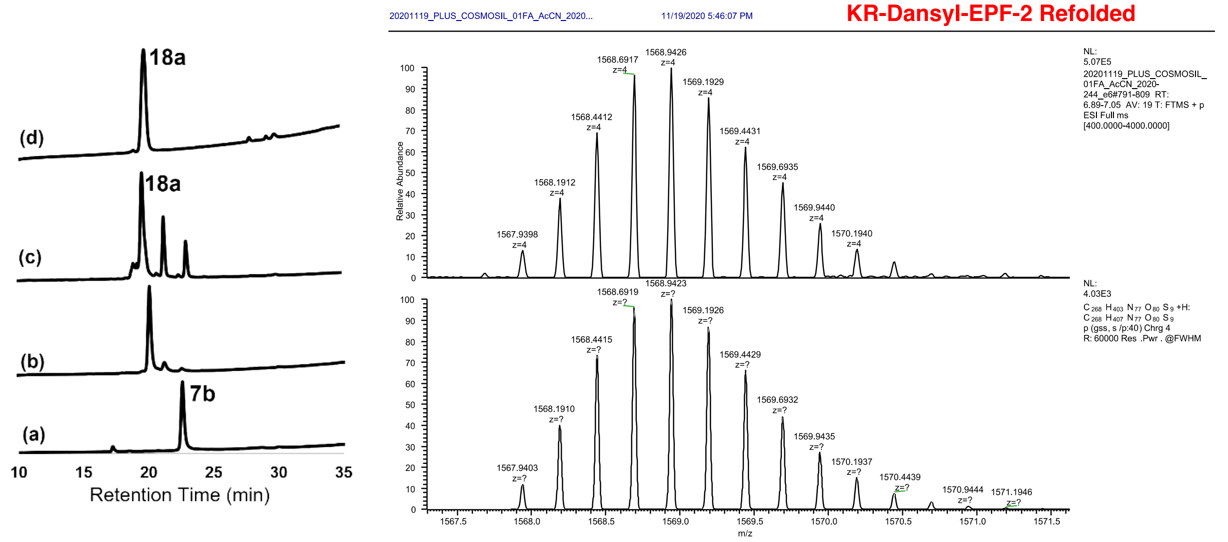

a) Photo-deprotection at 0 min; b) Photo-deprotection at 30 min; c) KAT Ligation at 20 min; d) Purified **18a**

7.9 Synthesis of coumarin-EPF2 18b

The coumarin conjugated EPF2 **18b** was synthesized according to general procedure **7.1** using folded photo-protected hydroxylamine EPF2 **7b** (1 mg, 0.165 μM) and coumarin KAT **16b** (0.11 mg, 0.198 μM). The crude mixture was purified by preparative RP-HPLC using Phenomenex Jupiter® C18 column (5 μm, 300 Å pore size, 21.2 mm I.D. × 250 mm) with a gradient of 20–80% CH_3CN with 0.1% TFA in 30 min, flow rate 10 mL/min. The fractions containing compounds pooled and lyophilized to give pure **18b** (0.74 mg, 72% yield). The purity and identity of **18b** was confirmed using analytical RP-HPLC and ESI-HRMS. The m/z calculated for **18b** $C_{266}H_{400}N_{76}O_{81}S_8$ $[M+4H]^{4+}$: 1552.6815 Da, measured 1552.6817 Da.

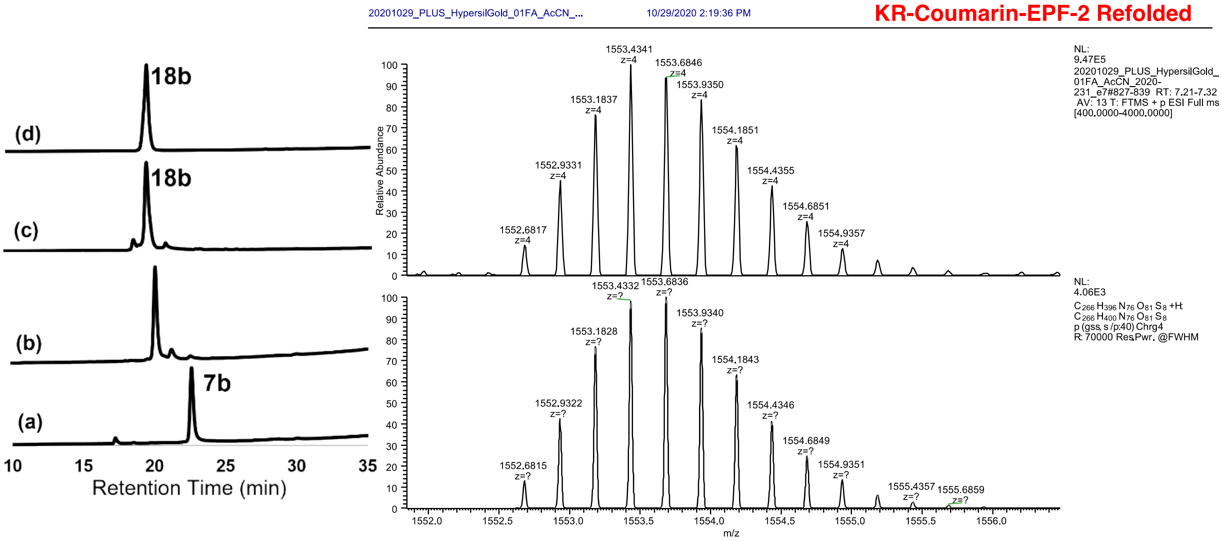

a) Photo-deprotection at 0 min; b) Photo-deprotection at 30 min; c) KAT Ligation at 20 min; d) Purified **18b**

7.10 Synthesis of pyrene-EPF2 18c

The pyrene conjugated EPF2 **18c** was synthesized according to general procedure **7.1** using folded photo-protected hydroxylamine EPF2 **7b** (1 mg, 0.165 μM) and pyrene KAT **16c** (0.11 mg, 0.198 μM). The crude mixture was purified by preparative RP-HPLC using Phenomenex Jupiter® C18 column (5 μm, 300 Å pore size, 21.2 mm I.D. × 250 mm) with a gradient of 20–80% CH_3CN with 0.1% TFA in 30 min, flow rate 10 mL/min. The fractions containing compounds pooled and lyophilized to give pure **18c** (0.61 mg, 60% yield). The purity and identity of **18c** was confirmed using analytical RP-HPLC and ESI-HRMS. The m/z calculated for **18c** $C_{271}H_{402}N_{75}O_{78}S_8$ $[M+4H]^{4+}$: 1552.6885 Da, measured 1552.6838 Da.

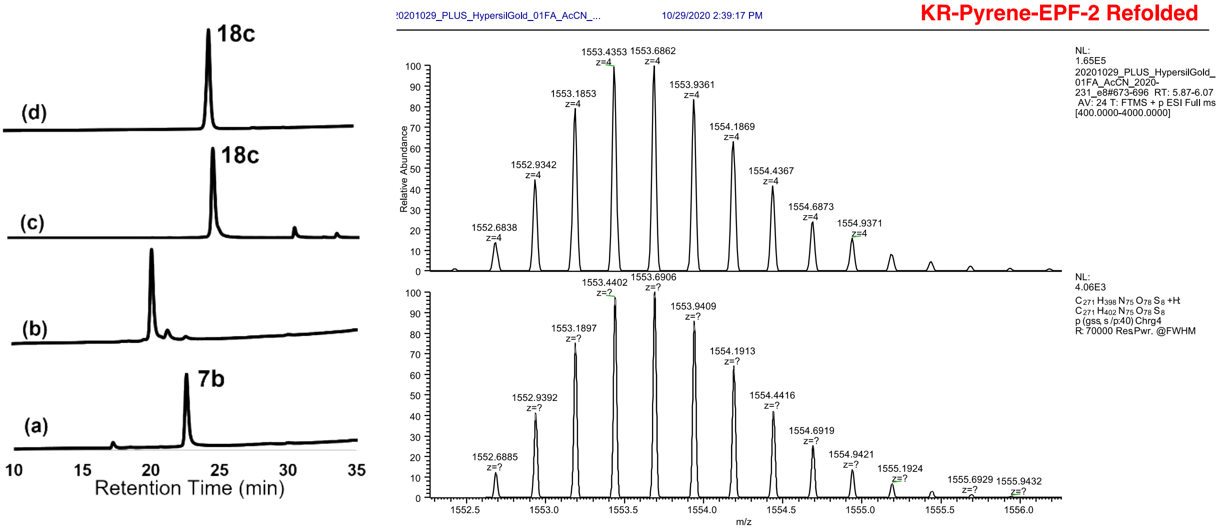

a) Photo-deprotection at 0 min; b) Photo-deprotection at 30 min; c) KAT Ligation at 20 min; d) Purified **18c**

7.11 Synthesis of BODIPY-EPF2 18d

The BODIPY conjugated EPF2 **18d** was synthesized according to general procedure **7.1** using folded photo-protected hydroxylamine EPF2 **7b** (1 mg, 0.165 µM) and BODIPY KAT **16d** (0.14 mg, 0.198 µM). The crude mixture was purified by preparative RP-HPLC using Phenomenex Jupiter® C18 column (5 µm, 300 Å pore size, 21.2 mm I.D. × 250 mm) with a gradient of 20–80% CH_3CN with 0.1% TFA in 30 min, flow rate 10 mL/min. The fractions containing compounds pooled and lyophilized to give pure **18d** (0.76 mg, 72% yield). The purity and identity of **18d** was confirmed using analytical RP-HPLC and ESI-HRMS. The m/z calculated for **18d** $C_{275}H_{412}BF_2N_{77}O_{79}S_8$ $[M+4H]^{4+}$: 1590.4606 Da, measured: 1590.4617 Da.

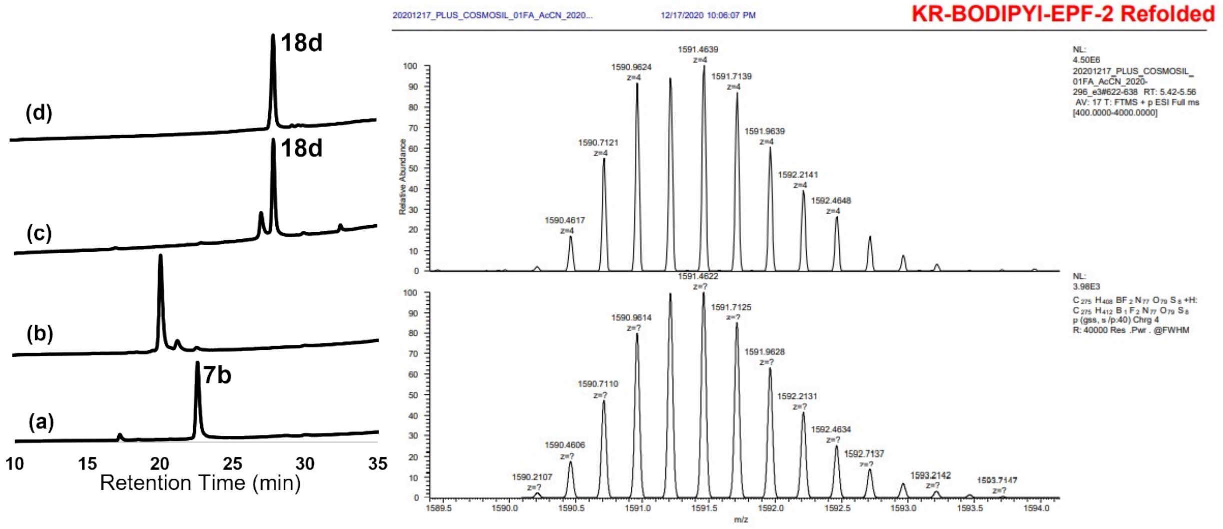

a) Photo-deprotection at 0 min; b) Photo-deprotection at 30 min; c) KAT Ligation at 20 min; d) Purified **18d**

7.12 Synthesis of Rhodamine-EPF2 18e

The rhodamine-B conjugated EPF2 **18e** was synthesized according to general procedure **7.1** using folded photo-protected hydroxylamine EPF2 **7b** (1 mg, 0.165 µM) and rhodamine-B KAT **16e** (0.16 mg, 0.198 µM). The crude mixture was purified by preparative RP-HPLC using Phenomenex Jupiter® C18 column (5 µm, 300 Å pore size, 21.2 mm I.D. × 250 mm) with a gradient of 20–80% CH_3CN with 0.1% TFA in 30 min, flow rate 10 mL/min. The fractions containing compounds pooled and lyophilized to give pure **18e** (0.8 mg, 75% yield). The purity and identity of the **18e** was confirmed using analytical RP-HPLC and ESI-HRMS. m/z calculated for **18e** $C_{284}H_{423}N_{77}O_{81}S_8$ $[M+3H]^{4+}$: 1615.9773 Da, measured: 1615.9709 Da.

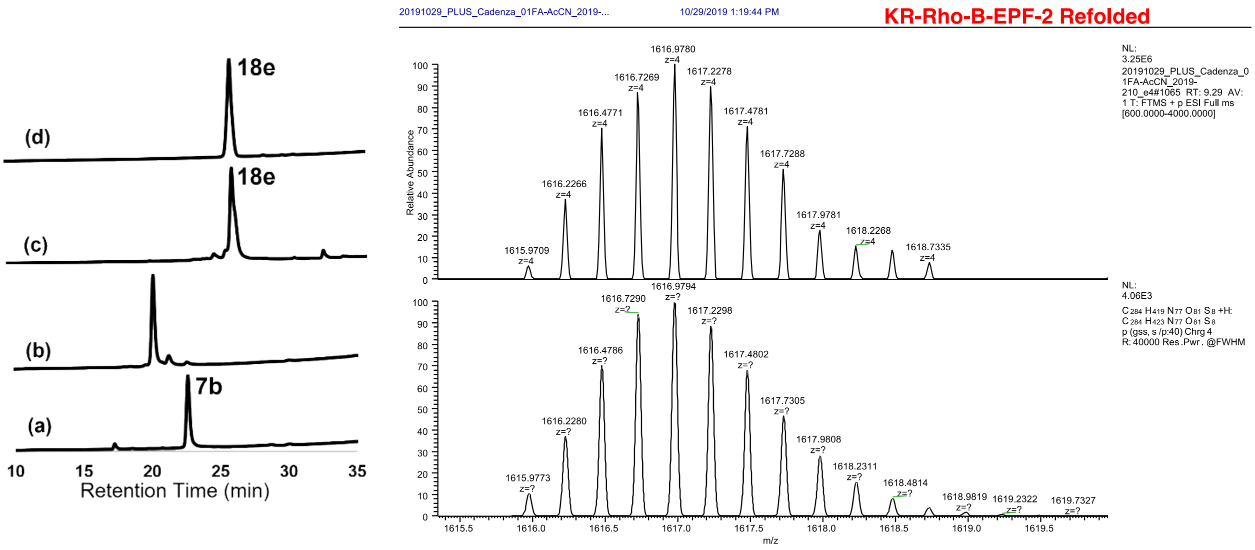

a) Photo-deprotection at 0 min; b) Photo-deprotection at 30 min; c) KAT Ligation at 20 min; d) Purified **18e**

7.13 Synthesis of Azido-EPF2 18f

The azido conjugated EPF2 **18f** was synthesized according to general procedure **7.1** using folded photo-protected hydroxylamine EPF2 **7b** (1 mg, 0.165 µM). The crude mixture was purified by preparative RP-HPLC using Phenomenex Jupiter® C18 column (5 µm, 300 Å pore size, 21.2 mm I.D. × 250 mm) with a gradient of 20–80% CH_3CN with 0.1% TFA in 30 min, flow rate 10 mL/min. The fractions containing compounds pooled and lyophilized to give pure **18f** (0.7 mg, 67% yield). The purity and identity of the **18f** was confirmed using analytical RP-HPLC and ESI-HRMS. The m/z calculated for **18f** $C_{253}H_{393}N_{75}O_{78}S_8$ $[M+6H]^{6+}$: 997.6137 Da, measured: 997.6176 Da.

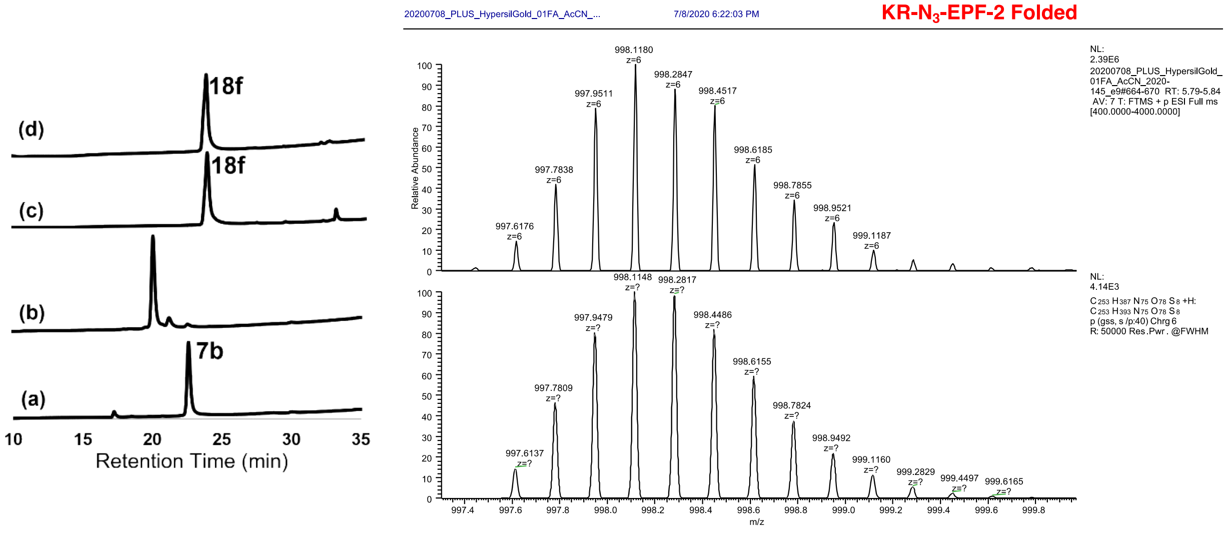

a) Photo-deprotection at 0 min; b) Photo-deprotection at 30 min; c) KAT Ligation at 20 min; d) Purified **18f**

7.14 Synthesis of dansyl-EPF1 19a

The dansyl dye conjugated EPF1 **19a** was synthesized according to general procedure **7.1** using folded photo-protected hydroxylamine EPF1 **13b** (1 mg, 0.173 µM) and dansyl KAT **16a** (0.13 mg, 0.207 µM). The crude reaction mixture was purified by preparative RP-HPLC using Phenomenex Jupiter® C18 column (5 µm, 300 Å pore size, 21.2 mm I.D. × 250 mm) with a gradient of 20–80% CH_3CN with 0.1% TFA in 30 min, flow rate 10 mL/min. The fractions containing compounds pooled and lyophilized to give pure **19a** (0.68 mg, 65% yield). The purity and identity of **19a** was confirmed using analytical RP-HPLC and ESI-HRMS. The m/z calculated for **19a** $C_{259}H_{398}N_{70}O_{77}S_9$ $[M+4H]^{4+}$: 1502.1711Da, measured: 1502.1710 Da.

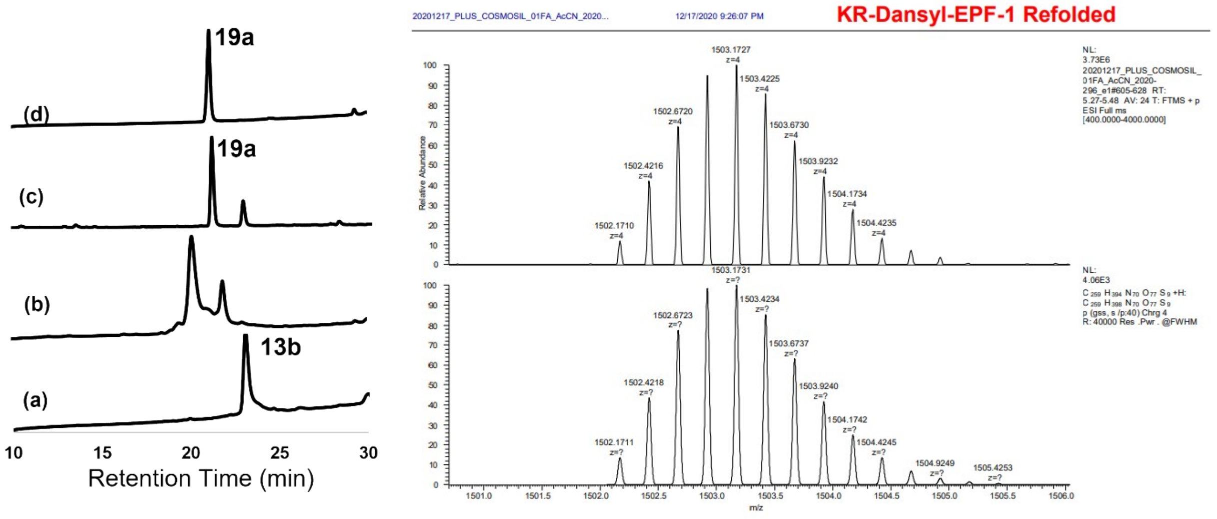

a) Photo-deprotection at 0 min; b) Photo-deprotection at 30 min; c) KAT Ligation at 20 min; d) Purified **19a**

7.15 Synthesis of coumarin-EPF1 19b

The coumarin dye conjugated EPF1 **19b** was synthesized according to general procedure **7.1** using folded photo-protected hydroxylamine EPF1 **13b** (1 mg, 0.173 µM) and coumarin dye KAT **16b** (0.12 mg, 0.207 µM). The crude reaction mixture was purified by preparative RP-HPLC using Phenomenex Jupiter® C18 column (5 µm, 300 Å pore size, 21.2 mm I.D. × 250 mm) with a gradient of 20–80% CH_3CN with 0.1% TFA in 30 min, flow rate 10 mL/min. The fractions containing compounds pooled and lyophilized to give pure **19b** (0.58 mg, 56% yield). The purity and identity of **19b** was confirmed using analytical RP-HPLC and ESI-HRMS. The m/z calculated for **19b** $C_{257}H_{391}N_{69}O_{78}S_8$ $[M+4H]^{4+}$: 1486.9124 Da, measured 1486.9123 Da.

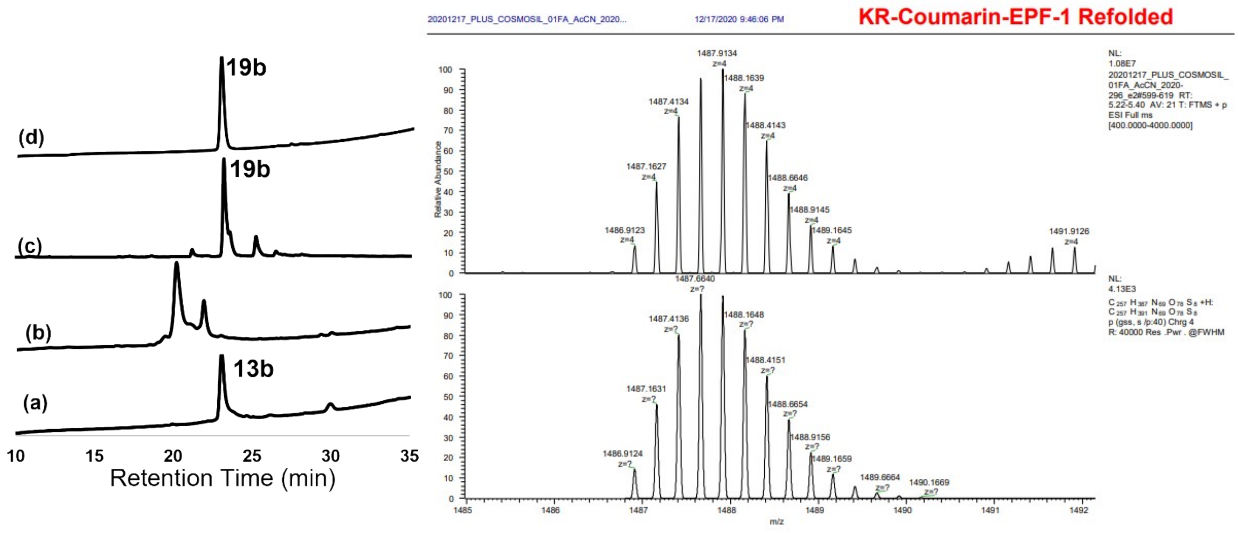

a) Photo-deprotection at 0 min; b) Photo-deprotection at 30 min; c) KAT Ligation at 20 min; d) Purified **19b**

7.16 Synthesis of pyrene-EPF1 19c

The pyrene conjugated EPF1 **19c** was synthesized according to general procedure **7.1** using folded photo-protected hydroxylamine EPF1 **13b** (1 mg, 0.173 μM) and pyrene dye KAT **16c** (0.12 mg, 0.207 μM). The crude mixture was purified by preparative RP-HPLC using Phenomenex Jupiter® C18 column (5 μm, 300 Å pore size, 21.2 mm I.D. × 250 mm) with a gradient of 20–80% CH_3CN with 0.1% TFA in 30 min, flow rate 10 mL/min. The fractions containing compounds pooled and lyophilized to give pure **19c** (0.59 mg, 57% yield). The purity and identity of **19c** was confirmed using analytical RP-HPLC and ESI-HRMS. m/z calculated for **19c** $C_{262}H_{392}N_{68}O_{75}S_8$ $[M+4H]^{4+}$: 1486.6674 Da, measured: 1486.6658 Da.

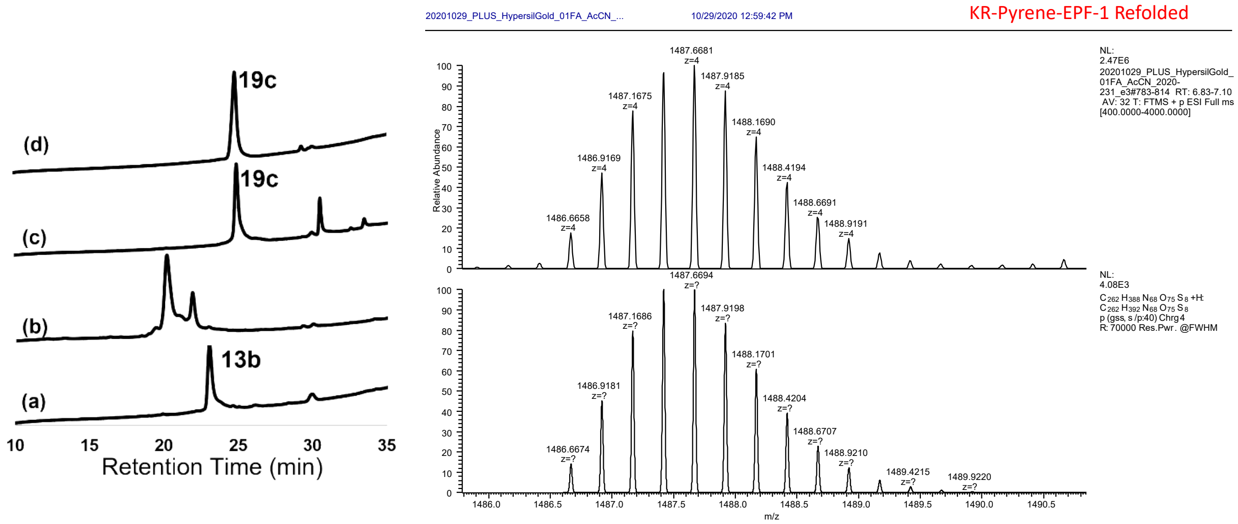

a) Photo-deprotection at 0 min; b) Photo-deprotection at 30 min; c) KAT Ligation at 20 min; d) Purified **19c**

7.17 Synthesis of BODIPY-EPF1 19d

The BODIPY conjugated EPF1 **19d** was synthesized according to general procedure **7.1** using folded photo-protected hydroxylamine EPF1 **13b** (1 mg, 0.173 μM) and BODIPY dye KAT **16d** (0.15 mg, 0.207 μM). The crude mixture was purified by preparative RP-HPLC using Phenomenex Jupiter® C18 column (5 μm, 300 Å pore size, 21.2 mm I.D. × 250 mm) with a gradient of 20–80% CH_3CN with 0.1% TFA in 30 min, flow rate 10 mL/min. The fractions containing compounds pooled and lyophilized to give pure **19d** (0.68 mg, 65% yield). The purity and identity of **19d** was confirmed using analytical RP-HPLC and ESI-HRMS. The m/z calculated for **19d** $C_{266}H_{403}BF_2N_{70}O_{76}S_8$ $[M+4H]^{4+}$: 1524.6914 Da, measured 1524.6903 Da.

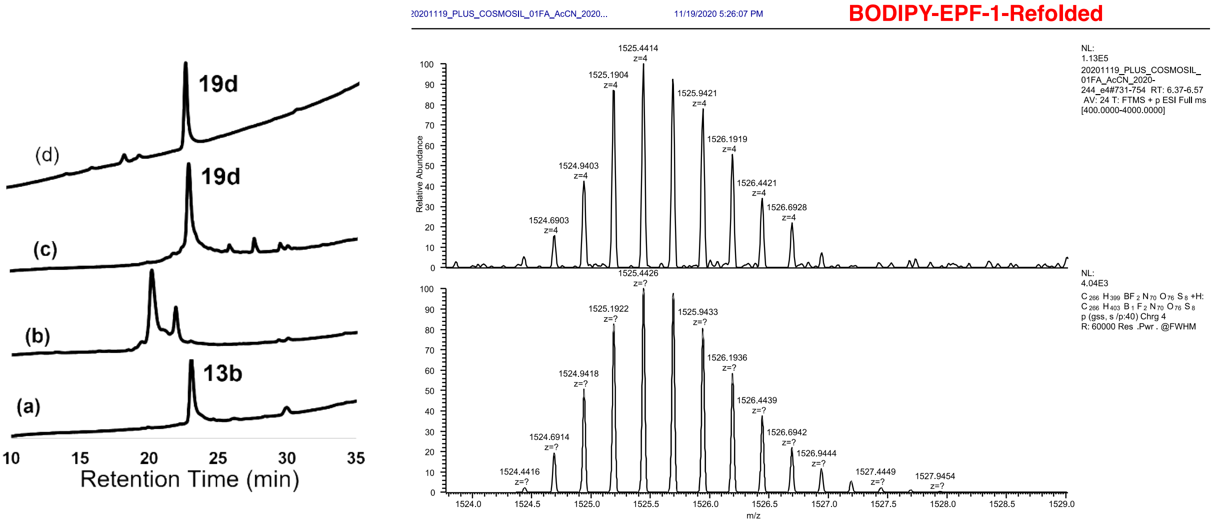

a) Photo-deprotection at 0 min; b) Photo-deprotection at 30 min; c) KAT Ligation at 20 min; d) Purified **19d**

7.18 Synthesis of Rhodamine-EPF1 19e

The rhodamine-B conjugated EPF1 **19e** was synthesized according to general procedure **7.1** using folded photo-protected hydroxylamine EPF1 **13b** (1 mg, 0.173 μM) and rhodamine-B dye KAT **16e** (0.17 mg, 0.207 μM). The crude mixture was purified by preparative RP-HPLC using Phenomenex Jupiter® C18 column (5 μm, 300 Å pore size, 21.2 mm I.D. × 250 mm) with a gradient of 20–80% CH_3CN with 0.1% TFA in 30 min, flow rate 10 mL/min. The fractions containing compounds pooled and lyophilized to give pure **19e** (0.64 mg, 60% yield). The purity and identity of **19e** was confirmed using analytical RP-HPLC and ESI-HRMS. The m/z calculated for **19e** $C_{275}H_{415}N_{70}O_{78}S_8$ $[M+4H]^{4+}$: 1550.4601 Da, measured: 1550.4518 Da.

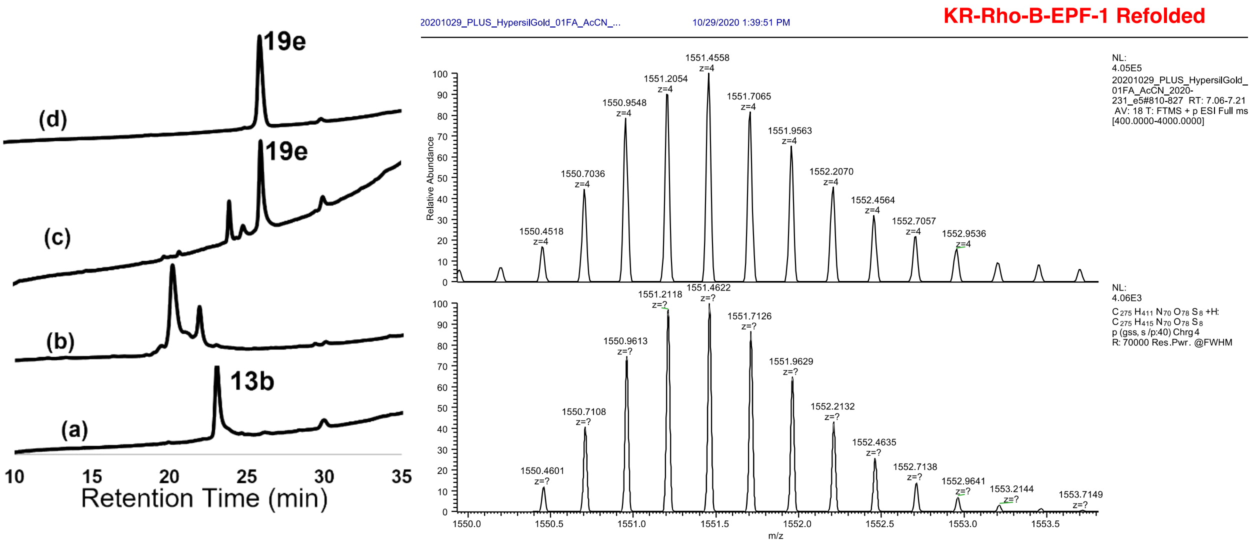

a) Photo-deprotection at 0 min; b) Photo-deprotection at 30 min; c) KAT Ligation at 20 min; d) Purified **19e**

7.19 Synthesis of Azido-EPF1 19f

The azido conjugated EPF1 **19f** was synthesized according to general procedure **7.1** using folded photo-protected hydroxylamine EPF1 **13b** (1 mg, 0.173 µM) and azido KAT **15** (0.07 mg, 0.207 µM). The crude mixture was purified by preparative RP-HPLC using Phenomenex Jupiter® C18 column (5 µm, 300 Å pore size, LC column 250 × 21.2 mm) with a gradient of 20–80% CH_3CN with 0.1% TFA in 30 min, flow rate 10 mL/min. The fractions containing compounds pooled and lyophilized to give pure **19f** (0.61 mg, 62% yield). The purity and identity of **19f** was confirmed using analytical RP-HPLC and ESI-HRMS. The m/z calculated for **19f** $C_{244}H_{383}N_{68}O_{75}S_8$ $[M+5H]^{5+}$: 1144.3197 Da, measured: 1144.3240 Da.

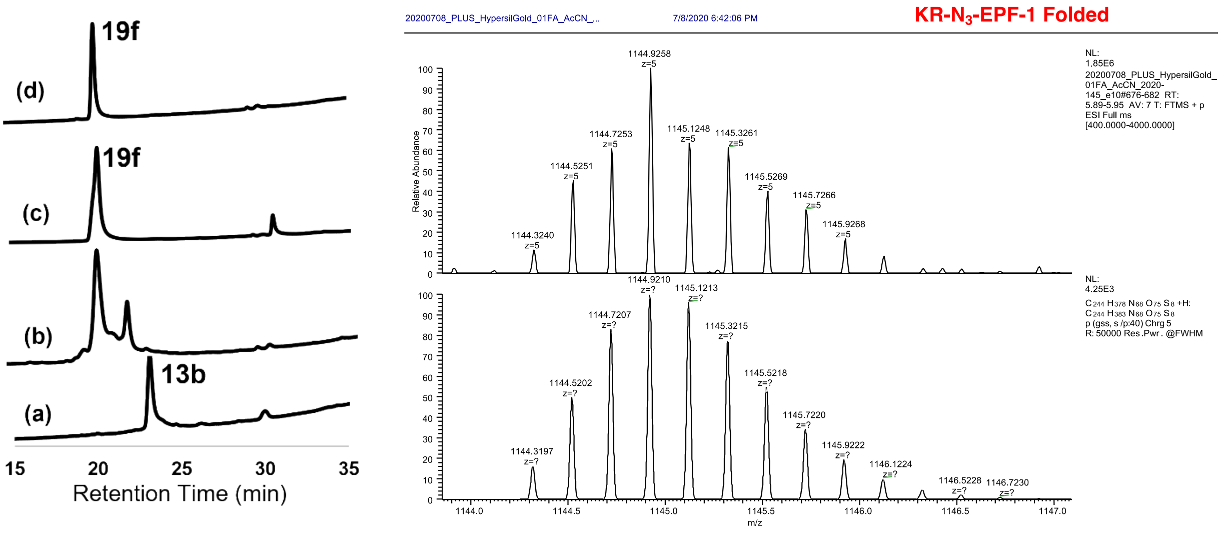

a) Photo-deprotection at 0 min; b) Photo-deprotection at 30 min; c) KAT Ligation at 20 min; d) Purified **19f**

8. Bioassay

8.1 Plant materials and growth conditions

Arabidopsis thaliana accession Columbia (Col) was used for bioassays. Plants were grown at 22 °C under continuous light (36 μmol^{-2} s^{-1}) for 7 days.

8.2 Peptide treatment

Evaluation of peptides was performed as previously described,[11] in which all peptides and fluorophores were dissolved in DMSO. Arabidopsis Col-0 seeds were sown in 96-well plates (TL5003; True Line) containing 95 μL of 1/2 Murashige and Skoog (MS) medium[12] with rotary shaking at 140 rpm under continuous light at 22 °C. Five μL of peptides (or fluorophores) dissolved in DMSO at 1 mM were diluted with liquid 1/2 MS media to 100 μM, and dropped on 1-day-old seedlings (final concentration, 5 μM). The abaxial epidermis cotyledons of 7-day-old seedling was imaged using confocal microscopy (see below). For visualizing BODIPY-EPFL9 **17d**, Col-0 seeds were grown for 7 days in liquid 1/2 MS medium, and transferred into new 1/2 MS medium containing 0.6 μM FM4-64 and 6 nM BODIPY-EPFL9 **17d** and with or without 6 nM folded-EPFL9 **2a** for 10 min. with rotary shaking at 40 rpm.

8.3 Confocal microscopy

The Zeiss LSM800 inverted confocal microscope (Oberkochen, Germany) was used for imaging. Cell peripheries were visualized by staining with propidium iodide (PI) (P4170; Sigma-Aldrich) using the following settings: 561 nm laser was used to excite PI. The emission filter was 582–617 nm for PI. For qualitative image presentation, Adobe Photoshop 2021 was used to trim and uniformly adjust the contrast/brightness. For visualization of BODIPY-EPFL9 and FM4-64 (T13320; ThermoFisher), 488 nm laser was used to excite BODIPY and 561 nm laser was used to excide FM4-64. The emission filter was 410–546 nm for BODIPY and 579–617 nm for FM4-64.

8.4 Quantitative analysis of stomatal density

Abaxial cotyledons from 7-day-old seedlings of respected genotypes or treatments were subjected to clearing solution. Specifically, samples were fixed a mixture of ethanol and acetic acid (9:1, v/v) at 4 °C and rehydrated in a graded ethanol series (70%, 50% and 30%) for 30 min in each solution, and transferred to clearing solution (a mixture of 8 g of chloral hydrate, 1 mL of glycerol, 2 mL of water) for at least overnight at 4 °C. The cleared samples were spread onto the slide glass

and observed under the microscope (Carl Zeiss AXIO Imager A2.) equipped with differential interference contrast (DIC) optics and the ZEN imaging software (ZEN2.3 Lite). The central regions overlying the distal vascular loop were imaged and numbers of stomata were quantified. For each genotype or chemical treatment, sample size of 7 to 10 was used and over thousand epidermal cells were counted to provide statistical robustness. Statistical analysis (ANOVA after Tukey's HSD test) was performed using RStudio (www.rstudio.com) version 1.4.1717 for stomatal density.

8.5 Quantitative analysis of bioactivity of fluorophores on stomatal formation

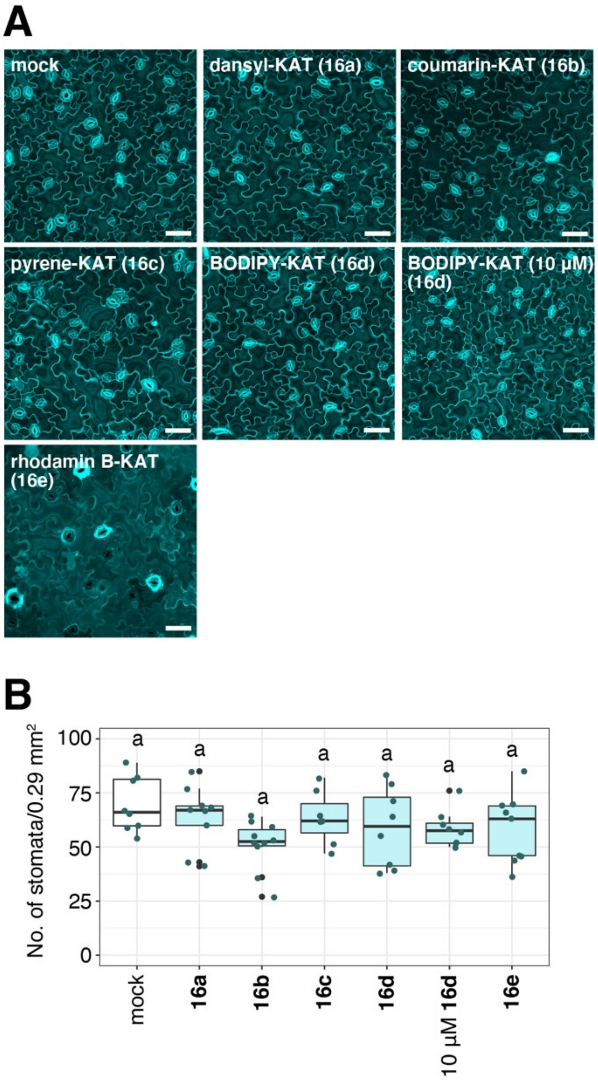

Figure S1. Fluorophores do not affect stomatal development. (A) representative confocal images of cotyledon abaxial epidermis from the 7-day-old Arabidopsis wild type Col-0 seedlings treated with mock, 5 µM fluorophore-KAT **16a–e**, For the BODIPY-KAT **16d** treatment, the image for 10 µM treatment is also shown. Scale bar = 50 µm. (B) quantitative analysis of the number of stomata shown as a box plot. Dots, individual data points. Median values are shown as lines in

the boxplot. ANOVA after Tukey's HSD test was performed for comparison of samples treated with the mock and each fluorophore. Number of leaves analyzed, n=8, 9, 10, 8, 8, 8, 9 for treatment with mock, 5 μM dansyl-KAT **16a**, 5 μM coumarin-KAT **16b**, 5 μM pyrene-KAT **16c**, 5 μM BODIPY-KAT **16d**, 10 μM BODIPY-KAT **16d**, 5 μM rhodamine B-KAT **16e**, respectively. All the same letters indicate no significant difference ($P > 0.05$).

9. References

[1] F. Thuaud, F. Rohrbacher, A. Zwicky, J. W. Bode, *Org. Lett.* **2016**, *18*, 3670–3673.
[2] C. E. Murar, T. J. Harmand, J. W. Bode, *Bioorg. Med. Chem.* **2017**, *25*, 4996–5001.
[3] G. N. Boross, D. Schauenburg, J. W. Bode, *Helv. Chim. Acta* **2018**, DOI 10.1002/hlca.201800214.
[4] T. Yoshiya, A. Taniguchi, Y. Sohma, F. Fukao, S. Nakamura, N. Abe, N. Ito, M. Skwarczynski, T. Kimura, Y. Hayashi, Y. Kiso, *Org. Biomol. Chem.* **2007**, *5*, 1720–1730.
[5] J.-S. Zheng, S. Tang, Y.-K. Qi, Z.-P. Wang, L. Liu, *Nat. Protoc.* **2013**, *8*, 2483–2495.
[6] B. Akgun, D. G. Hall, *Angew. Chem. Int. Ed Engl.* **2016**, *55*, 3909–3913.
[7] L. Guo, A. I. Suarez, M. R. Braden, J. M. Gerdes, C. M. Thompson, *Bioorg. Med. Chem. Lett.* **2010**, *20*, 1194–1197.
[8] J. Sun, X. Xu, G. Yu, W. Li, J. Shi, *Tetrahedron* **2018**, *74*, 987–991.
[9] N. G. Patil, N. B. Basutkar, A. V. Ambade, *Chem. Commun.* **2015**, *51*, 17708–17711.
[10] A. Schachtschneider, M. Wessig, M. Spitzbarth, A. Donner, C. Fischer, M. Drescher, S. Polarz, *Angew. Chem. Int. Ed Engl.* **2015**, *54*, 10465–10469.
[11] A. Ziadi, N. Uchida, H. Kato, R. Hisamatsu, A. Sato, S. Hagihara, K. Itami, K. U. Torii, *Chem. Commun.* **2017**, *53*, 9632–9635.
[12] T. Murashige, F. Skoog, *Physiol. Plant.* **1962**, *15*, 473–497.
